# Intercepting Methanimine for the Synthesis of Piperidine-Based *N*‑Heterocycles in an Aqueous Medium

**DOI:** 10.1021/acs.joc.5c01213

**Published:** 2025-07-26

**Authors:** Emily Pocock, Martin Diefenbach, Thomas M. Hood, Michael Nunn, Vera Krewald, Simon E. Lewis, Ruth L. Webster

**Affiliations:** † Department of Chemistry, 1555 University of Bath, Claverton Down, Bath BA2 7AY, U.K.; ‡ Department of Chemistry, TU Darmstadt, Peter-Grünberg-Str. 4, Darmstadt 64287, Germany; § Early Chemical Development, 468087 Pharmaceutical Sciences, Biopharmaceuticals R&D, AstraZeneca, Macclesfield SK10 2NA, U.K.; ∥ Yusuf Hamied Department of Chemistry, 2152 University of Cambridge, Cambridge CB2 1EW, U.K.

## Abstract

We herein present an investigation into whether simple methodology could be used to intercept the highly reactive interstellar molecule methanimine. The use of an *in situ* aza-Diels–Alder reaction to trap out methanimine as simple piperidine-based *N*-heterocycles was explored. Subsequent investigations into alternative dienes revealed that the steric and electronic nature of the diene had a great effect on its effectiveness in trapping methanimine. While the yields of the resultant *N*-heterocycles are modest, the products formed are novel yet structurally simple and could be envisioned to be highly synthetically useful building blocks for further transformations. We also explored simple protecting groups that could be used to access a methanimine adduct as a discrete synthon, but density functional theory calculations indicated that cyclotrimerization, and thus deactivation, was likely.

## Graphical abstract

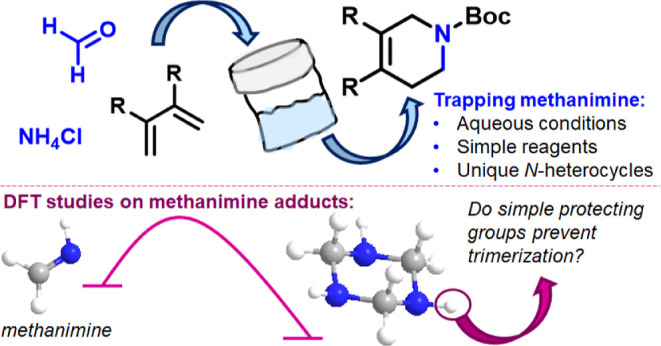

## Introduction

Methanimine, H_2_C=NH, is one of the simplest organic building blocks and has been postulated to have played a key role in prebiotic chemistry.^1–4^ This is largely due to its detection in Titan’s upper atmosphere, which has an organic haze layer that has been likened to Earth’s early atmosphere. Since its detection^5^ scientists have tried to elucidate its role in prebiotic synthesis. Early work suggested that it acted as a precursor for the formation of aminoacetonitrile, one of the key molecules in Strecker-type synthesis, which is a proposed prebiotic mechanism for the formation of glycine (Scheme 1a). More recently, Sutherland and co-workers have proposed several new methods for the prebiotic synthesis that all involve methanimine as a key intermediate.^4,6^

**Scheme 1:**
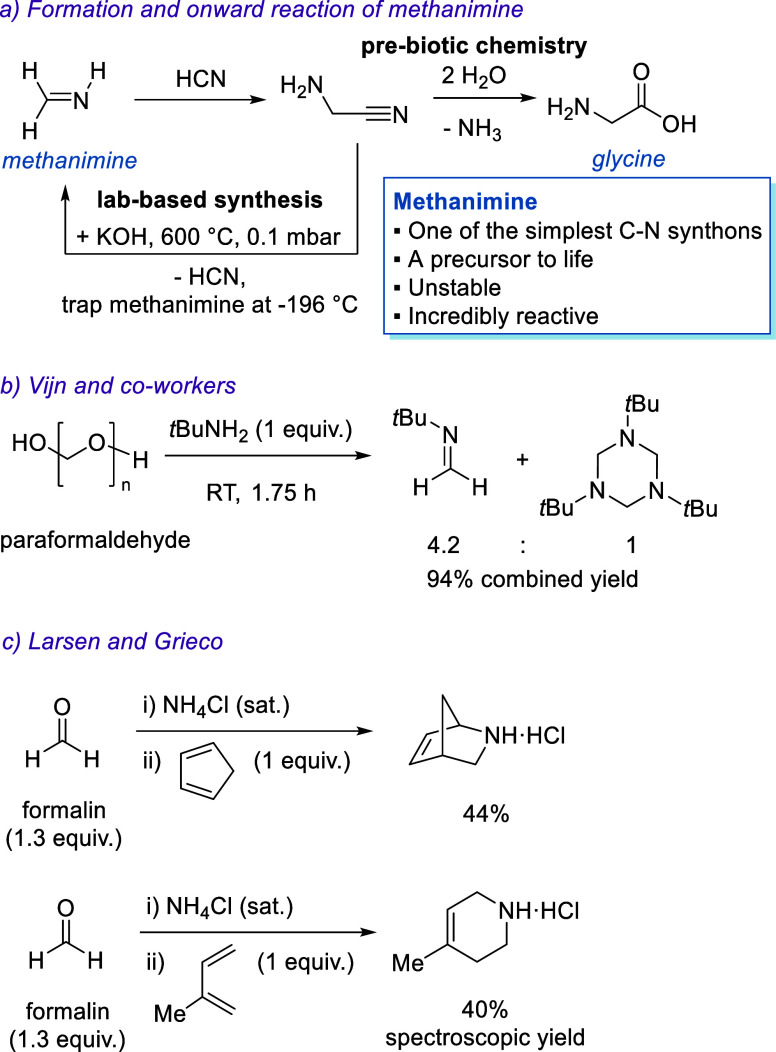
(a) Formation and Isolation of Methanimine in the Lab Requires Harsh Conditions, while Potential onward Reactions in the Intersteller Medium include the Formation of Glycine; (b) Formation of *N*-Methylene-*tert*-butylamine *via* the Reaction of *para*-Formaldehyde and *tert*-Butylamine; and (c) Cyclo-Condensation of Dienes with Simple Iminium Salts Generated under Mannich Conditions as Reported by Larsen and Grieco

Despite being a synthetically interesting molecule, methanimine has only been prepared and isolated a handful of times. A principal route to methanimine in the laboratory is to heat 2-aminoacetonitrile to 600 °C under a 0.1 mbar vacuum. This releases toxic hydrogen cyanide gas (which must be quenched by the reaction with KOH) and methanimine, which is trapped in a quartz U-tube at −196 °C (Scheme 1a).^7–9^ Other routes^10^ include the elimination of HCl from *N*-chloromethylamine, the drawback being that the precursor itself is a highly sensitive reagent.^11–14^ Cryogenic photolysis of azidomethane leading to the loss of N_2_ has also been employed along with^15^ pyrolysis of methylamine in a quartz flow tube heated to 1000 °C^16,17^ and retro-aza-Diels–Alder reactions.^18,19^ Once formed, methanimine is incredibly unstable and will rapidly form oligomeric amines within a few hours at −77 °C.

In order to access methanimine, trapping as an adduct is clearly necessary. Vijn and co-workers reported the formation of *N*-methylene-*tert*-butylamine by the reaction of bulky *tert*-butylamine and paraformaldehyde (Scheme 1b). The desired imine did form in combination with its corresponding cyclotrimer in a ratio of monomer/trimer 4.2:1, showing that the added bulk bound to the nitrogen slightly inhibits trimerization.^20^ This is based on the work reported by Cromwell^21^ and earlier by Hurwitz,^22^ where 37% aqueous formaldehyde solution (herein referred to as formalin) was employed. The drawback of these works is the use of a *tert*-butyl protecting group, which is not easy to remove or exchange for other, potentially more useful, functionality.

The work conducted by Larsen and Grieco is relevant for the identification of potential trapping agents for the formation of methanimine using formalin. The group reported that a range of unactivated iminium salts, generated *in situ* under Mannich-like conditions from formalin, could be reacted with dienes via a mild aqueous aza-Diels–Alder reaction. Their research primarily focused on the use of benzylamine hydrochloride, with six examples reported. This paper is of particular interest because Larsen and Grieco briefly examined the use of ammonium chloride as the amine component, which resulted in the formation of two novel secondary amines, albeit in modest yields (Scheme 1c).^23^ While this reactivity was not discussed or examined further, it indicates that an *in situ* Diels–Alder reaction is an effective method for trapping out methanimine. Furthermore, the reaction also yields synthetically useful cyclic amines, which are challenging to access via other methods. More recently, Grison and co-workers employed plant extracts, containing Zn, that were deposited on montmorillonite K10 in a range of Diels–Alder reactions.^24^ This included the reaction of cyclopentadiene with formaldehyde and ammonia (mixed *in situ*) in an aqueous solution. After 6 h at 25 °C, the authors reported an 84% GC–MS yield. Skvarchenko et al. have also reported the use of formalin, NH_4_Cl in an EtOH/water mix for an aza-Diels–Alder reaction of an anthracene derivative.^25^ The reaction solvent employed by Larsen and Grieco^23^ was water, as is the case for the reactions we report herein. Using water as the reaction medium for Diels–Alder reactions in general,^26^ including aza-Diels–Alder reactions,^27,28^ is associated with rate accelerations. The reasons for this phenomenon may vary between specific reactions; various explanations have been advanced.^29–33^

## Results and Discussion

Considering methanimine’s distinct reactivity as an intermediate, attention was turned to optimizing the *in situ* formation of methanimine. We initiated studies by focusing on 2,3-dimethyl-1,3-butadiene as the trapping agent in aqueous aza-Diels–Alder chemistry.^27,28^ Ammonium chloride and formalin were used due to their cost effectiveness and ready availability.

An initial test reaction was conducted on an NMR scale using ammonium chloride and formalin (both 1 equiv) with a slight excess of diene (1.5 equiv) to determine whether this would be a suitable system to investigate. After 16 h at 60 °C, there is clear evidence that the desired product forms (4,5-dimethyl-1,2,3,6-tetrahydropyridine, **1a**), albeit in trace amounts. After this initial result, the reaction was conducted in NH_4_Cl (sat.) in a sealed ampule to maximize methanimine formation and trapping. We also changed the ratio of diene; as this is the most expensive component, it was used as the limiting reagent.

Running the reaction for 24 h at 40 °C gives 6% **1a** (Table 1, entry 1).^34^ Despite this low conversion, there are no other obvious byproducts present in the reaction, indicating that either the diene is unable to enter into solution or, once in solution, it degrades into baseline byproducts not easily observed by ^1^H NMR spectroscopy. Wang and co-workers reported the use of lanthanide triflates as effective Lewis acid catalysts for the aza-Diels–Alder reactions in aqueous media, in particular the use of ytterbium­(III) triflate was found to greatly increase rate of reaction.^35^ However, in our model system, the addition of 10 mol % Yb­(OTf)_3_ shows no noticeable increase in product conversion (entry 2).

**Table 1:**
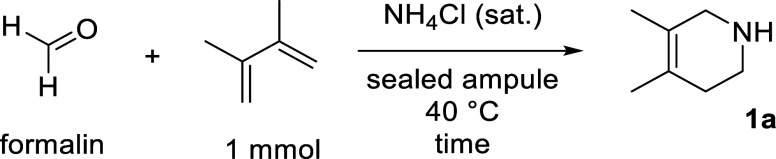
Optimization Table for the Aza-Diels–Alder Reaction Using *In Situ* Generated Methanimine

entry	conc. (M)	formalin (mmol)	time (h)	spectroscopic yield^a^ (%)
1	0.5	1.3	24	6
2^b^	0.5	1.3	24	9
3	0.5	1.3	64	32
4^c^	0.5	1.3	64	14
5^d^	0.5	1.3	64	24
6^e^	0.5	1	64	18
7^e^	1	1	64	22
8	1	1.3	64	37
9	1.5	1.3	64	29
10	2	1.3	64	34
11	0.5	1.3	120	39
12^f^	0.5	1.3	64	7
**13** ^g^	**0.5**	**1.3**	**64**	**42**

a Spectroscopic yield refers to yield as determined by ^1^H NMR spectroscopy using crude reaction mixture. Calculated against 1 equiv of maleic acid added at the end of the reaction as an internal standard.

b 10 mol % Yb­(OTf)_3_ added to the reaction mixture.

c Reaction conducted in a 30 mL sample vial.

d 1 mL MeCN as an additive.

e 3 mmol diene used.

f Reaction performed at 60 °C.

g Reaction performed at 45 °C.

Leaving the reaction for longer leads to an increase in conversion from 6% to 32% after 64 h at 40 °C (entry 3). This suggests that at these temperatures the initial formation of the methanimine intermediate *via* the Mannich type reaction might be slow. Repeating the reaction in a 30 mL sample vial results in a significant decrease in conversion to product from 32% to 14% compared with the corresponding reaction conducted in an ampule (entry 4).

We also considered that the lack of solubility of the diene in the aqueous solvent could be a potential issue. However, the addition of MeCN does not improve the conversion (entry 5). Adding the diene in excess (3 mmol) and using formalin as the limiting reagent reduced the yield (18%, entry 6). Increasing the concentration gives a slight improvement (compare entry 3 to entry 8 and entry 6 to entry 7), but the reaction is clearly finely balanced because further increasing the concentration (1.5 and 2 M, entries 9 and 10, respectively) impacts the conversion to product (29% and 34%, respectively). This may be due to 0.5 M conditions also allowing for more efficient stirring. Leaving the reaction for longer (5 days, entry 11) does not lead to any appreciable increase in yield, while increasing the temperature to 60 °C in an attempt to reduce reaction time reduces the yield to 7% (entry 12). However, a more modest increase in temperature to 45 °C improves yield: 42% **1a** is obtained (entry 13).

The drastic change in the yield when the reaction was conducted in a 30 mL sample vial (**D**, Figure 1) was unexpected, and therefore, it was clear that further investigations into the reaction vessel were needed. To probe this, reactions were setup simultaneously in seven different reaction vials and left for 120 h at 45 °C (**A**–**G**, Figure 1). After this time, a spectroscopic yield was obtained compared to an internal standard. The control reactions reveal that the choice of reaction vessel does impact formation of **1a**, where a medium reagent bottle (**E**, Figure 1) and 7 mL sample vial (**G**) give rise to the best spectroscopic yields (54% and 52%, respectively). Considering this, 7 mL sample vials were employed for ease of reproducibility and the ability to run several reactions in parallel. Due to the change of the reaction vessel a second optimization investigation was conducted (see the Supporting Information), but our optimized conditions for the formation of **1a** continue to be a 0.5 M solution of 1 mmol diene and 1.3 mmol formalin prepared from a saturated NH_4_Cl solution, stirred at 45 °C for 64 h. The spectroscopic yield for this reaction is 52%.

**Figure 1:**
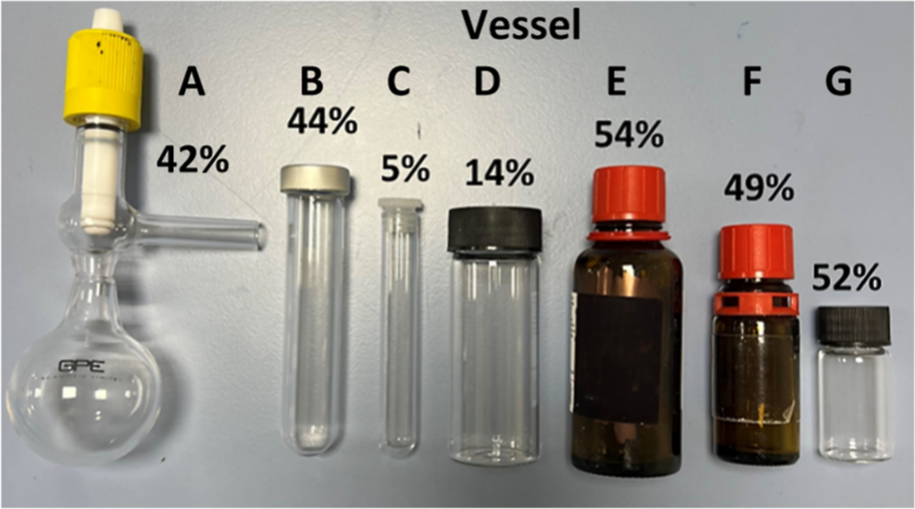
Investigations into how the choice of reaction vessel affects product formation for the aza-Diels–Alder reaction of methanimine. Ampule (25 mL, **A**), microwave vial (2–5 mL size, **B**), 4 mL push-cap vial (**C**), 30 mL vial (**D**), medium reagent bottle (standard contents = 25 mL commercial reagent, **E**), small reagent bottle (standard contents = 5 mL commercial reagent, **F**), and 7 mL vial (**G**).

Additional control reactions were conducted to better understand the reactivity: using *para*-formaldehyde instead of formalin significantly reduces the formation of **1a** (Scheme 2a). This could be due to the reaction temperature, which is not sufficient to convert *para*-formaldehyde to formaldehyde in any appreciable amount. However, it is worth noting that employing *para*-formaldehyde with NH_4_Cl (sat.) and 2,3-dimethylbuta-1,3-diene in a range of organic solvents (MeCN, THF, DMF, pyridine, MeOH) at RT or 40 °C fails to generate **1a**, while undertaking the same reaction of *para*-formaldehyde where the saturated NH_4_Cl solution is the only source of the solvent (at 60 °C to aid *para*-formaldehyde dissolution) only gives 6% **1a**. It is also worth reinforcing the fact that some aza-Diels–Alder reactions have been reported as being reversible in water at 50 °C,^18,19^ which could explain why this chemistry appears to be so capricious. Delaying the addition of diene (to determine whether methanimine could build up in solution to enable more efficient product formation) leads to a significant decrease in conversion (Scheme 2b) indicating the diene needs to be present from the start of the reaction in order to effectively trap out methanimine. We anticipate that methanimine forms as the protonated species under reaction conditions,^28,36,37^ but to determine whether a reduction in the headspace while maintaining concentration could improve the product yield the reaction was undertaken on a 2 mmol scale (Scheme 2c). However, after 64 h at 45 °C the conversion was 6%, which could be linked to a lower headspace but we cannot rule out a lack of tolerance of scale-up or less efficient stirring and heating within the small sample vial. To better understand how vital NH_4_Cl is, two different nucleophilic nitrogen sources, methyl carbamate and trifluoromethylsulfonamide, were employed in the reaction (Scheme 2d,e). No reaction occurs after 64 h at 45 °C suggesting that these two alternative ammonia surrogates are not nucleophilic enough to undergo the initial methanimine formation.

**Scheme 2:**
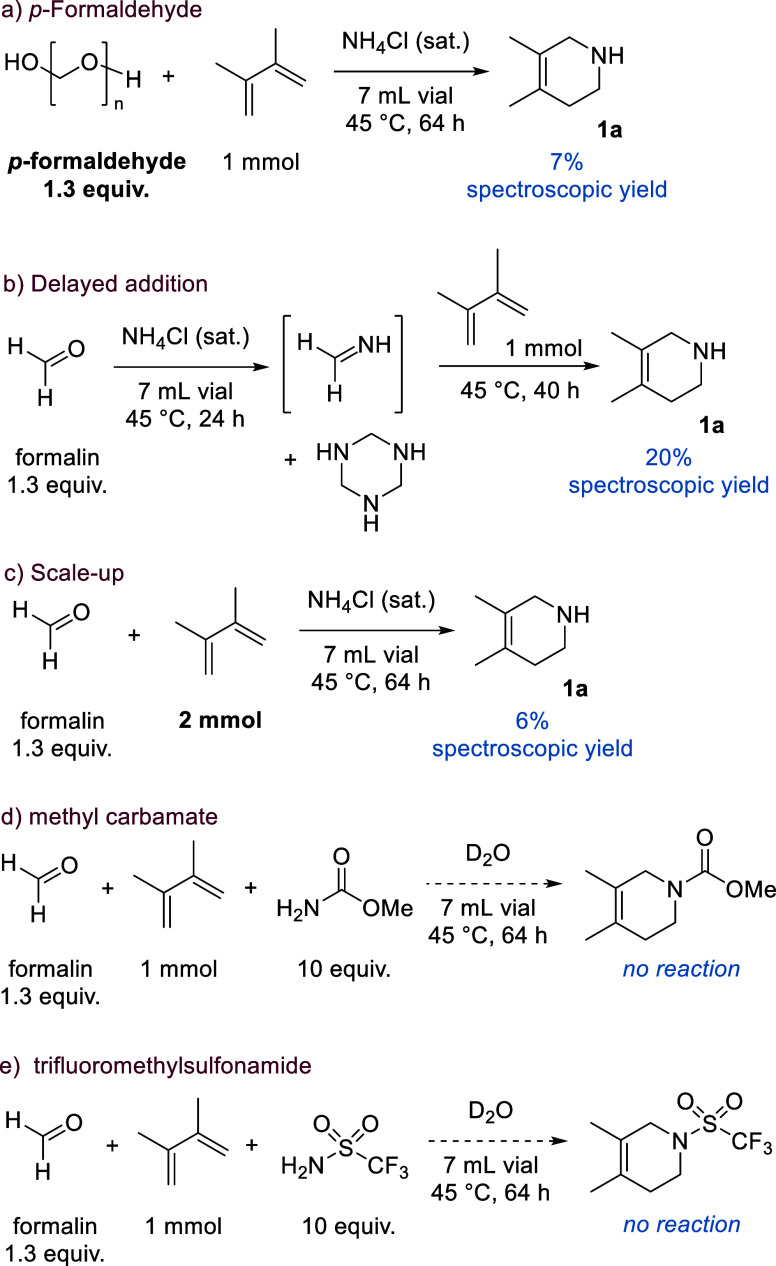
Control Reactions for the *In Situ* aza-Diels–Alder Reaction Conducted on a 1 mmol Scale;^a^ a) *Para*-formaldehyde Gives Minimal **1a** Formation; b) Delayed Diene Addition Reduces Conversion; c) Scale-up (2 mmol) Yields Only 6% **1a**; d) Methyl Carbamate and e) Trifluoromethylsulfonamide Do Not Form **1a**

a Spectroscopic yield refers to yield as determined by ^1^H NMR spectroscopy of the crude reaction mixture.

### Scope and Limitations

We turned our attention to simple hydrocarbon-based dienes, and pleasingly, these substrates are suitable for the aza-Diels–Alder reactions (Scheme 3). *In situ* protection using Boc anhydride was used to aid isolation (to generate **1a′**, **1b** to **1e**, Scheme 3). Reactions are regioselective, with **1b**, **1c**, and **1d** forming as one regioisomer only. A range of product yields are observed, indicating that the type of diene has a dramatic effect on reactivity. Several factors can be thought to play a role in how effective the diene will be for trapping methanimine, for example, cyclohexadiene is “locked” in the s-*cis* geometry, which means that no reorganization is required prior to the interaction with the methanimine intermediate. This could be a factor contributing to why this diene appears to show an improved yield (compare Scheme 3, **1d** and **1e**). In the case of **1c**, sterics are likely to be responsible for the low yield because the geminal dimethyl groups will hinder the formation of the required transition state. In the case of **1b** and **1c**, the dienes employed are relatively volatile with boiling points of 34 and 42 °C for 2-methyl-1,3-butadiene and *trans*-1,3-pentadiene, respectively. This volatility could explain why the yields are significantly lower than that of **1a′** because the diene might be lost to the vessel headspace. While the yields of the piperidine derivatives are lower than desired, it is important to note that the products formed are structurally simple cyclic amines, which would be highly challenging to access through other synthetic routes. In the case of **1c** and **1d**, the products produced are previously unreported.

**Scheme 3:**
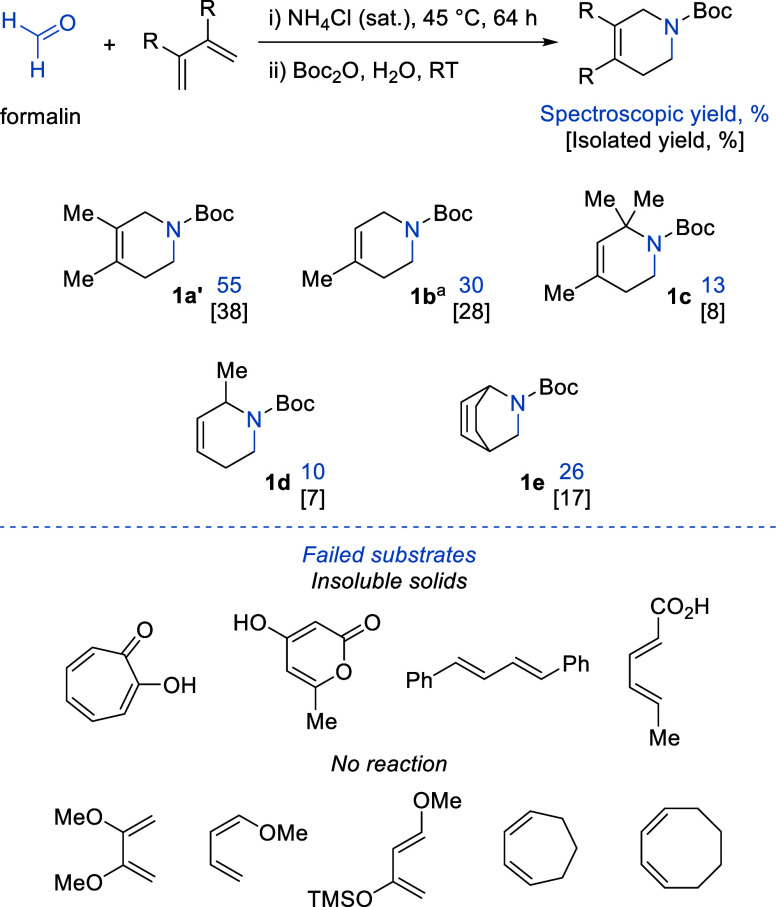
Diene Scope for the *In Situ* aza-Diels–Alder Reaction Using Methanimine^a^

a Reaction conditions: 1 mmol diene, 1.3 mmol formalin, 0.5 M solution prepared using NH_4_Cl (sat.). Spectroscopic yield refers to yield as determined by ^1^H NMR spectroscopy of the crude reaction mixture. Calculated using 1 mmol maleic acid added at the end of the reaction. ^a^Reaction conducted at 30 °C due to the volatility of diene.

Unfortunately, the reaction is highly sensitive to trapping reagents (Scheme 3). Several of the dienes tested are solid reagents; these did not dissolve in the reaction mixture and failed to react. Reactions using 1,3-cyclooctadiene and 1,3-cycloheptadiene give NMR spectra that show that the desired products do not form, and the dienes remain unreacted. Employing 1-methoxy-1,3-butadiene or 2,3-dimethoxy-1,3-butadiene gives a color change from colorless to dark brown. The ^1^H NMR spectra show that the aza-Diels–Alder reaction had not taken place, instead the dienes appear to degrade.^38^ When comparing cyclohexadiene to cycloheptadiene, the former is more reactive toward methanimine. This trend in reactivity is explained by the work of Levandowski and Houk, who stated that in order to achieve the required transition state for the Diels–Alder reaction to proceed, out of plane distortion across the diene double bonds is vital.^39^

To gain some additional information about the aza-Diels-Al-der reaction, the transition state structures were calculated for our standard reaction using density functional theory (Figure 2 and Supporting Information). The computed barrier of 29.4 kcal mol^–1^ for the aza-Diels–Alder reaction between methanimine and 2,3-dimethylbutadiene is in agreement with a slow reaction at elevated temperatures. Note, however, that when an acid-catalyzed reaction is assumed in aqueous media, the methanimine reactant will mostly occur in its protonated form. In this scenario, the reaction barrier is expected to be significantly lowered,^35^ but the overall reaction is reversible at elevated temperatures.^18,19^ Thus, both the neutral and the proton-assisted reaction are in agreement with the experimentally observed yields.

**Figure 2:**
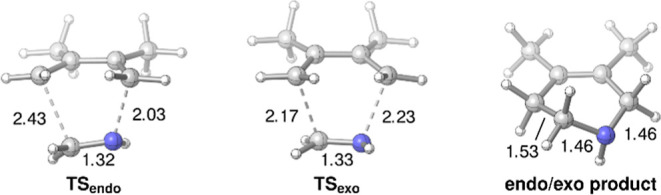
Transition structures and product for the aza-Diels–Alder reaction of methanimine with *trans*-2,3-dimethyl butadiene. Computed at the PBE0-D3/def2-TZVP/PCM­(acetonitrile)//PBE-D3/def2-SVP/PCM (acetonitrile) level.

Given the challenge of intercepting methanimine, we wondered whether we could access a protected variant that would allow the use of methanimine as a discrete synthon. Vijn and co-workers showed that *in situ* protection using a *tert*-butyl group is a viable option,^20^ but arguably, has a limited synthetic scope due to the challenges associated with removing the *tert*-butyl group. We, therefore, turned our attention to more labile *N*-protecting groups; borane and silane groups could have the level of steric protection required to inhibit the cyclotrimerization of a methanimine adduct (**2**, Figure 3a), but they could be labile enough to undergo facile cleavage or hydrolysis after an organic transformation has taken place. We specifically targeted commercially available protecting groups, pinacol borane (Bpin) and *tert*-butyldimethylsilyl (TBDMS) where we envisaged a route to the protected primary amine using NH_3_ followed by condensation with *para*-formaldehyde in the presence of a desiccant. We used DFT calculations to predict the likelihood of the barrier to cyclotrimerization (forming **3**, Figure 3a) with the aim that this should be substantial enough to stabilize the methanimine adduct as the monomer. Cyclotrimerization of methanimine (**2a**, Figure 3b) proceeds with a sizable activation barrier of 39.3 kcal mol^–1^, but the formation of the cyclic product **3a** is exergonic, with Δ_r_ *G* = −30.3 kcal mol^–1^ relative to **2a**. The Bpin-protected methanimine adduct **2b** exhibits a lower barrier to cyclotrimerization (Δ^⧧^ *G* = 26.4 kcal mol^–1^), and the corresponding product **3b** is significantly more stable (Δ_r_ *G* = −57.1 kcal mol^–1^, Figure 3c), indicating that **2b** is more reactive than the unprotected species. In contrast, TBDMS-protected methanimine **2c** undergoes cyclotrimerization with a slightly higher barrier of 37.1 kcal mol^–1^, yielding **3c** at −27.9 kcal mol^–1^ relative to **2c** (Figure 3d). These results suggest that such simple protecting group strategies do not divert reactivity away from the [2 + 2 + 2] cyclization pathway. Furthermore, assuming the presence of some protonated methanimine, the sequential formation of a protonated cyclic variant of **3a** can proceed via significantly lower barriers (see the Supporting Information), offering a potential mechanism for accelerating the cyclization reaction.

**Figure 3:**
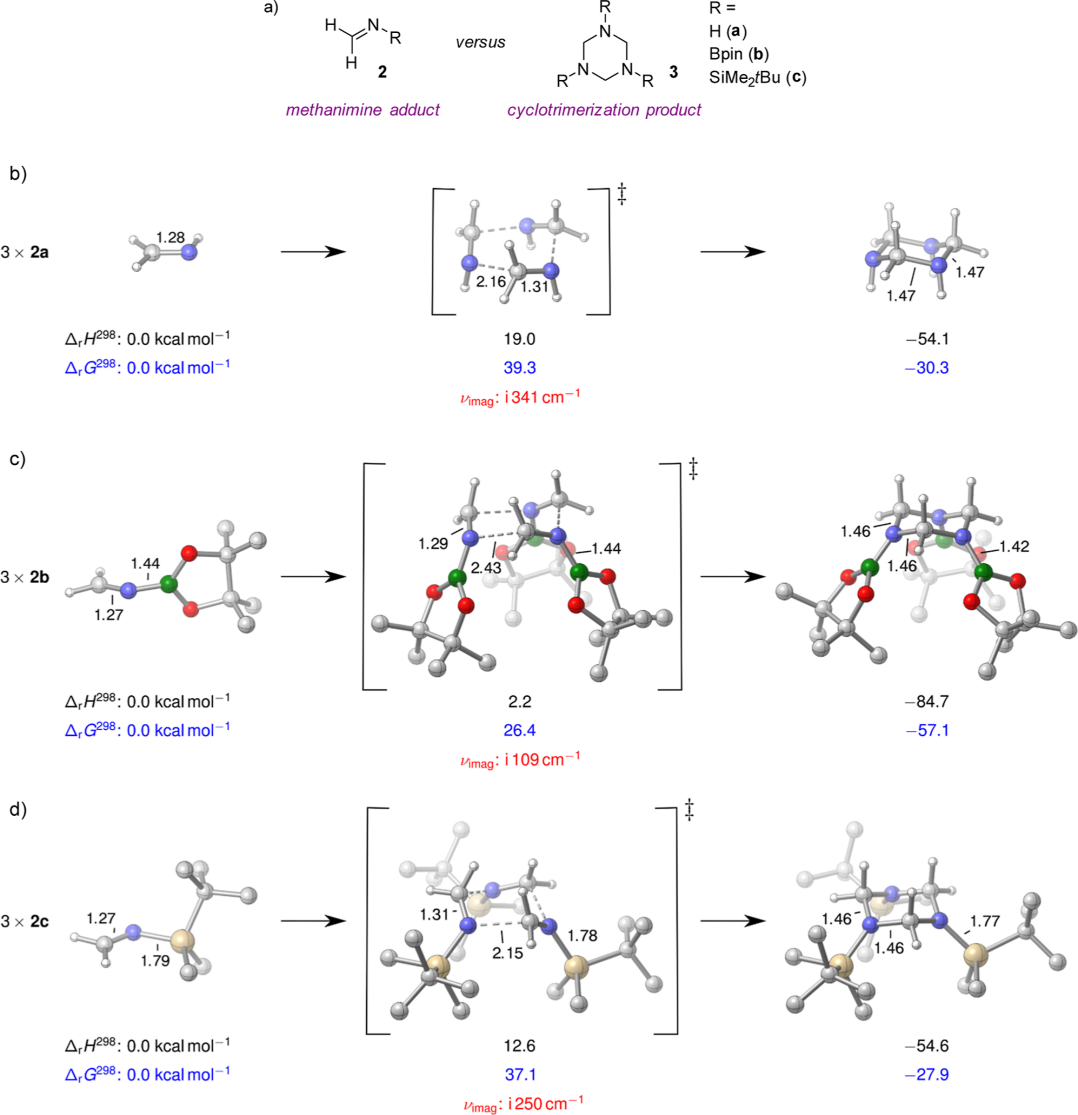
(a) Protected methanimine adducts considered for this *in silico* study are Bpin-protected (**2b**) and TBDMS-protected (**2c**). Reaction barriers and thermochemistry computed at the PBE0-D3/def2-TZVP/PCM­(acetonitrile)//PBE-D3/def2-SVP/PCM­(acetonitrile) level. Gibbs free energies for transition structures and cyclization products are corrected for standard-state conditions by −3.79 kcal mol^–1^. (b) The barrier to cyclotrimerization was first considered for methanimine (**2a**) and compared to (c) **2b** versus **3b** and (d) **2c** versus **3c**.

In short, although there is an energy barrier to the trimerization of the Bpin and TBDMS adducts (**2b** and **2c**, respectively), the reactions are highly exergonic, and these monomeric forms are unlikely to be the major species isolated from their synthesis.

## Conclusions

This work focused on whether a simple method could be employed to trap the highly reactive methanimine moiety. The work by Larsen and Grieco highlighted the potential of using an *in situ* aza-Diels–Alder reaction to sequester methanimine as simple piperidine-based *N*-heterocycles. We aimed to build on this, but investigations into the reaction conditions found that this is a highly capricious system, where the vessel size plays a significant role that affects reactivity. Through various optimization attempts, the conversion was improved from 5% to 55% spectroscopic yield when 2,3-dimethyl butadiene was used. Subsequent investigations into alternative dienes revealed that the steric and electronic nature of the diene affected the trapping of methanimine. While the yields of the resultant *N*-heterocycles are modest, the use of simple aqueous conditions, coupled with the fact that the products are novel, mean this could be a synthetically useful route to nitrogen-containing building blocks for further transformations. Our *in silico* optimization of a discrete protected methanimine synthon reveals that steric bulk is not enough to prevent cyclotrimerization, and the effort and cost of preparing even bulkier protecting groups are unlikely to outweigh the benefits of the aqueous aza-Diels–Alder methodology reported here.

## Experimental Section

See the Supporting Information.

## Supplementary Material





## Data Availability

The data underlying this study are available in the published article and its Supporting Information.

## Notes

Experimental section including general methods, reaction procedure, spectroscopic data, and computational data­(PDF)
Cartesian coordinates of computed molecular structures (ZIP)
