# Supplementary material for: Intercepting Methanimine for the Synthesis of Piperidine-Based N‑Heterocycles in an Aqueous Medium
Source: J Org Chem. 2025 Jul 26;90(31):11257–63. doi: 10.1021/acs.joc.5c01213 (PMC12340960; doi:10.1021/acs.joc.5c01213)
Supplement: Supplementary file 1 [file jo5c01213_si_001.pdf]

Intercepting Methanimine for the Synthesis of Piperidine-based *N*-Heterocycles in Aqueous Medium

Emily Pocock,[a] Martin Diefenbach,[b‡] Thomas M. Hood,[a‡] Michael Nunn,[c] Vera Krewald,[b*]

Simon E. Lewis,[a*] Ruth L. Webster[d*]

[a]Department of Chemistry, University of Bath, Claverton Down, Bath, BA2 7AY, U.K.
[b]Department of Chemistry, TU Darmstadt, Peter-Grünberg-Str. 4, 64287 Darmstadt, Germany
[c]Early Chemical Development, Pharmaceutical Sciences, Biopharmaceuticals R&D, AstraZeneca, Macclesfield SK10 2NA, U.K.
[d]Yusuf Hamied Department of Chemistry, University of Cambridge, Cambridge CB2 1EW, U.K.

Contents

	Page
General Methods	S2
Safety Statement	S2
Optimization using 7 mL vials	S3
General Procedure	S3
Analysis Data	S4
Computational Details	S38
References	S42

1. General Methods

All chemicals were purchased from commercial sources (organic reagents from Merck Sigma, salts from Fisher Scientific) and used as supplied.
^{1}H and $^{13}C\{^{1}H\}$ NMR spectra were recorded on Bruker Advance 400 MHz NMR spectrometers. In $CDCl_3$, ^{1}H and $^{13}C\{^{1}H\}$ NMR chemical shifts are reported relative to $CHCl_3$ at 7.26 ppm and 77.16 ppm, respectively. Coupling constants (*J*) are reported in Hertz (Hz). Multiplicities are indicated by: br s (broad singlet), s(singlet), d (doublet), t (triplet), q (quartet) and m (multiplet) app. (apparent). Structural assignments were made with additional information from gCOSY, gHSQC, and gHMBC experiments.
Infrared (IR) spectra of neat compounds were recorded at ambient temperature over the range 4000–650 cm^{-1} using a PerkinElmer Spectrum 100 ATR-FTIR spectrometer using a diamond ATR unit. Peaks are reported in cm^{-1}.
For mass spectrometry a microTOF electrospray time-of-flight (ESI TOF) mass spectrometer (Bruker Daltonik GmbH, Bremen, Germany) was used. Data are reported in the form of *m/z*. The observed mass and isotope pattern matched the corresponding theoretical values as calculated from the expected molecular formula.
Analytical thin-layer chromatography was performed on Merck silica gel 60 F254 aluminium-backed plates. Visualisation was accomplished with UV light (254 nm), and vanillin stain. Automated flash column chromatography (normal phase) was performed using a CombiFlash NextGen 300+ system equipped with UV and ELSD detectors, using 4 - 40g silica columns.

2. Safety Statement

Caution! The dienes employed in this study are all highly flammable with flammable vapors. Although employed in small (mmol) quantities, care should be taken when handling. Reaction mixtures must be allowed to cool to room temperature before work-up.

Boc anhydride and formalin are flammable and toxic liquids. They should be handled in a fumehood, and as with all experiments, appropriate PPE should be worn.

Cyclic amine products **1c** and **1d** are novel and should be treated as harmful/toxic, along with compounds **1a'**, **1b** and **1e**.

3. Optimization of Reaction Using 7 mL Vials

Table S1. Additional optimisation table for the in-situ aza-Diels–Alder reaction conducted in vials using methanimine.

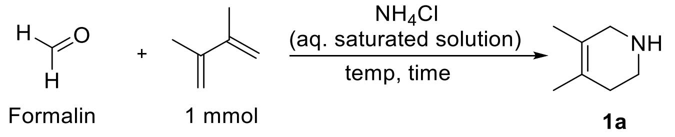

Concentration (M)	Formalin (equiv.)	Temperature (°C)	Time (h)	Spectroscopic yield (%)
0.5	1.3	45	120	52
0.5	1.3	45	64	52
1	1.3	45	64	43
1.5	1.3	45	64	38
2	1.3	45	64	38
0.4	1.3	45	64	54
0.3	1.3	45	64	43
0.5	1.3	55	64	11
0.5	3	45	64	14
0.5	5	45	6	2
0.5	1	45	64	31
0.5	1.3	45	24	19
0.5	1.3	45	48	38

Reaction Conditions: Reactions were conducted on a 1 mmol scale in a sealed 7 mL sample vial. Spectroscopic yields (as determined by ^1H NMR spectroscopy using crude reaction mixture) were calculated against 1 equivalent of maleic acid added at the end of the reaction as an internal standard.

4. General Procedure for the Synthesis of N-Heterocycles via *in situ* Methanimine Formation

To three individual 7 mL sample vials each equipped with a magnetic stirrer bar was added 2 mL saturated ammonium chloride solution. Then to each vial diene was added (1 mmol, 1 equiv.) and finally formaldehyde solution (7 wt. % in H_2O) (96.8 µL, 1.3 mmol, 1.3 equiv.). The vials were then sealed in a sand bath and stirred at 40°C for 64h.

After this time the three individual vials were combined and aqueous NaOH was added until a pH 10 was obtained. Then BOC anhydride (0.65 g, 3 mmol, 3 equiv.) was added and the resulting solution was stirred at room temperature and the reaction was monitored by TLC until it reached completion. The organics were then isolated via separation using ethyl acetate (3×5 mL) and these organics were then concentrated in situ to give a crude oil.

The organics were then redissolved in ethanol and imidazole (0.304 g, 3 equiv.) was added to react with the excess BOC anhydride. This mixture was stirred at room temperature for 1 h and then the desired product was isolated via column chromatography using 2:8 ethyl acetate: hexane).

5. Analysis Data

tert-butyl 4,5-dimethyl-3,6-dihydropyridine-1(2H)-carboxylate, **1a'**

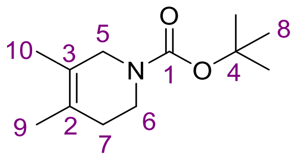

Following the general procedure and using 2,3-dimethyl butadiene (113.1 µL, 1 mmol per vial)

Yield: 0.244 g, 38% (colourless oil).

Analytic data is in accordance with those reported in literature.[1]

R_f = 0.65 (20% ethyl acetate / 80% hexane)

^{1}H NMR ($CDCl_3$, 400 MHz): δ 3.67 (*br.* s, 2H, C^5-H), 3.42 (t, *J* = 5.9 Hz, 2H, C^6-H), 1.99 (*br.* s, 2H, C^7-H), 1.62 (s, 3H, C^9-H), 1.58 (s, 3H, C^{10}-H), 1.44 (s, 9H, C^8-H).

^{13}C{^{1}H} NMR ($CDCl_3$, 101 MHz) δ 154.9 (C^1), 124.9 (rotamers) (C^2), 122.9 (rotamers) (C^3), 79.4 (C^4), 47.8/47.5 (rotamers) (C^5), 41.4/40.1 (rotamers) (C^6), 31.1 (C^7), 28.6 (C^8), 18.8 (C^9), 16.1 (C^{10}).

^{13}C{^{1}H} NMR ($CDCl_3$, 101 MHz, -25°C) δ 155.0/154.8 (C^1), 124.9/124.4 (C^2), 123.1/122.5 (C^3), 79.6/79.4 (C^4), 47.6/46.9 (C^5), 41.1/39.7 (C^6), 30.9/30.7 (C^7), 28.5/28.5 (C^8), 18.9/18.8 (C^9), 16.2/16.2 (C^{10}).

HRMS (ESI+) m/z: $[M + Na]^+$ Calcd for $C_{12}H_{21}NO_2Na^+$ 234.1464; Found 234.1467.

FT-IR (cm^{-1}): 2975.8, 1697.8, 1418.3, 1391.4, 1364.5, 1210.2 cm^{-1}.

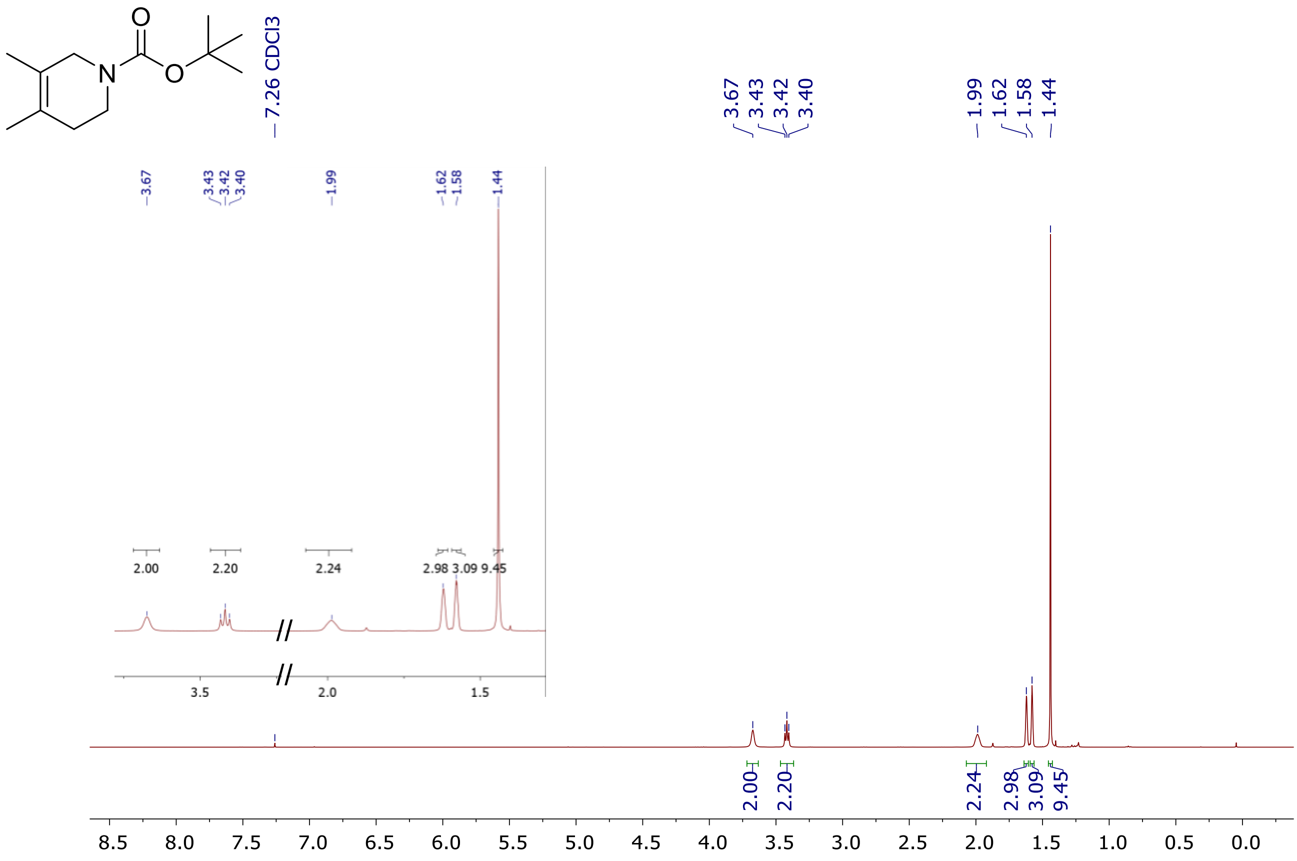

Figure S1: ^{1}H NMR spectrum (400 MHz) of **1a'** in $CDCl_3$ after isolation via column chromatography. Inset shows zoom region.

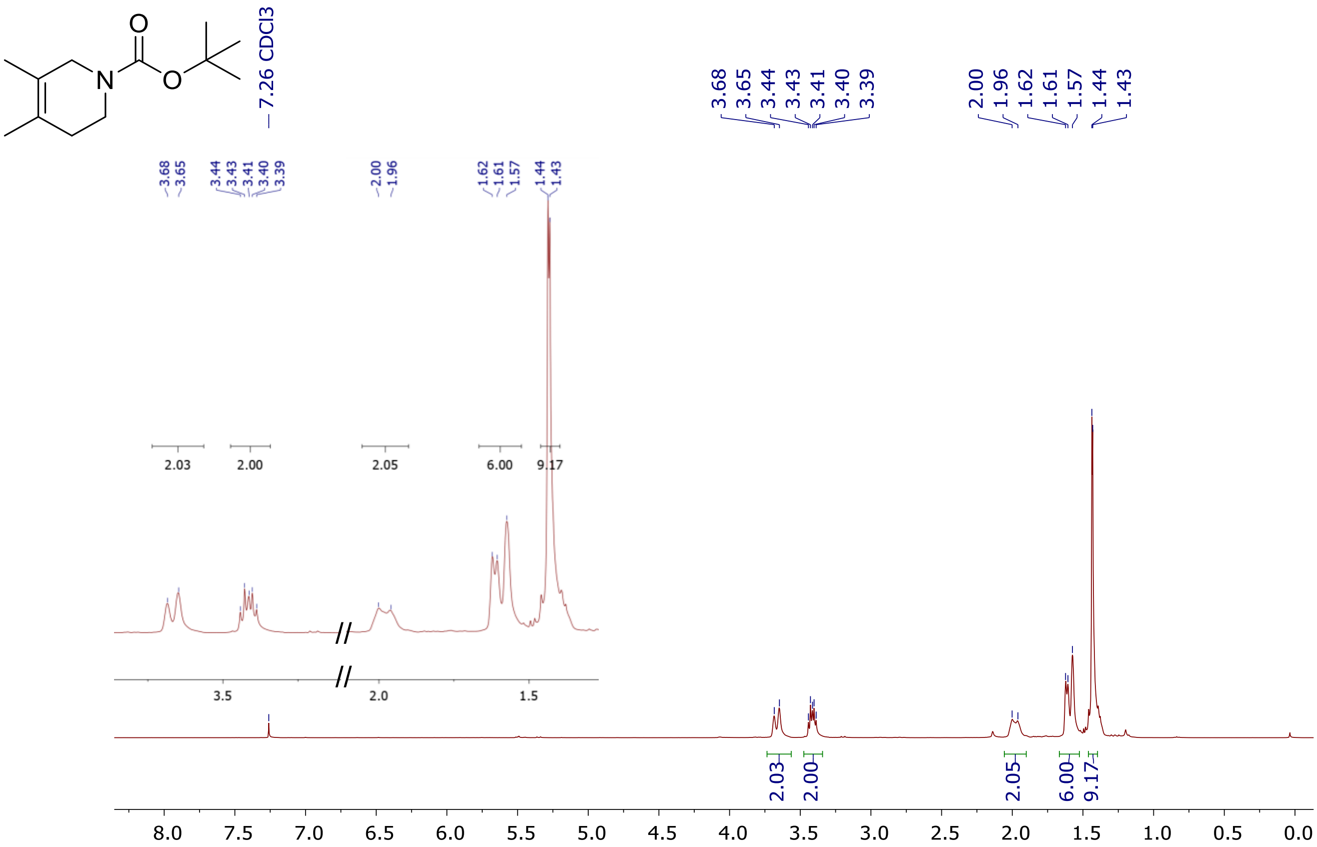

Figure S2: ^{1}H NMR spectrum (400 MHz) of **1a'** in $CDCl_3$ after isolation via column chromatography. Spectrum recorded at −25°C. Inset shows zoom region.

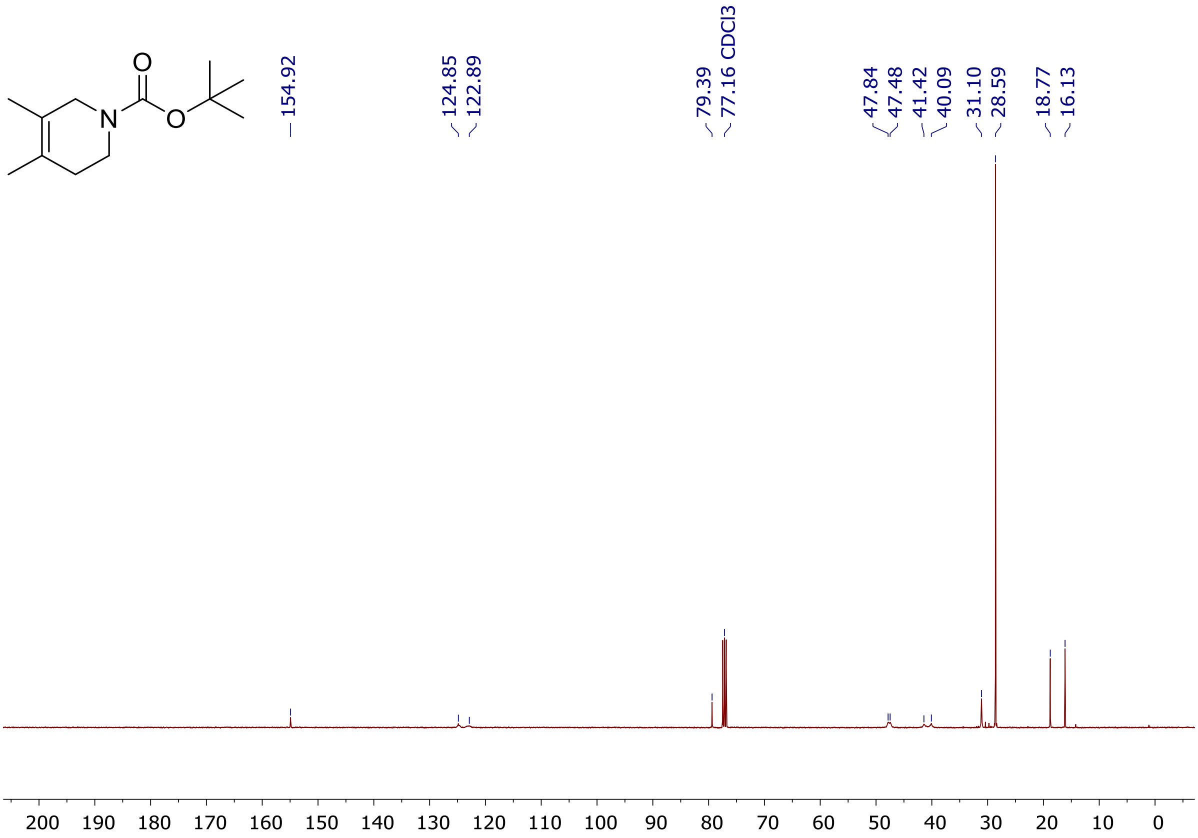

Figure S3: $^{13}C\{^1H\}$ NMR spectrum (101 MHz) of **1a'** in $CDCl_3$ after isolation via column chromatography.

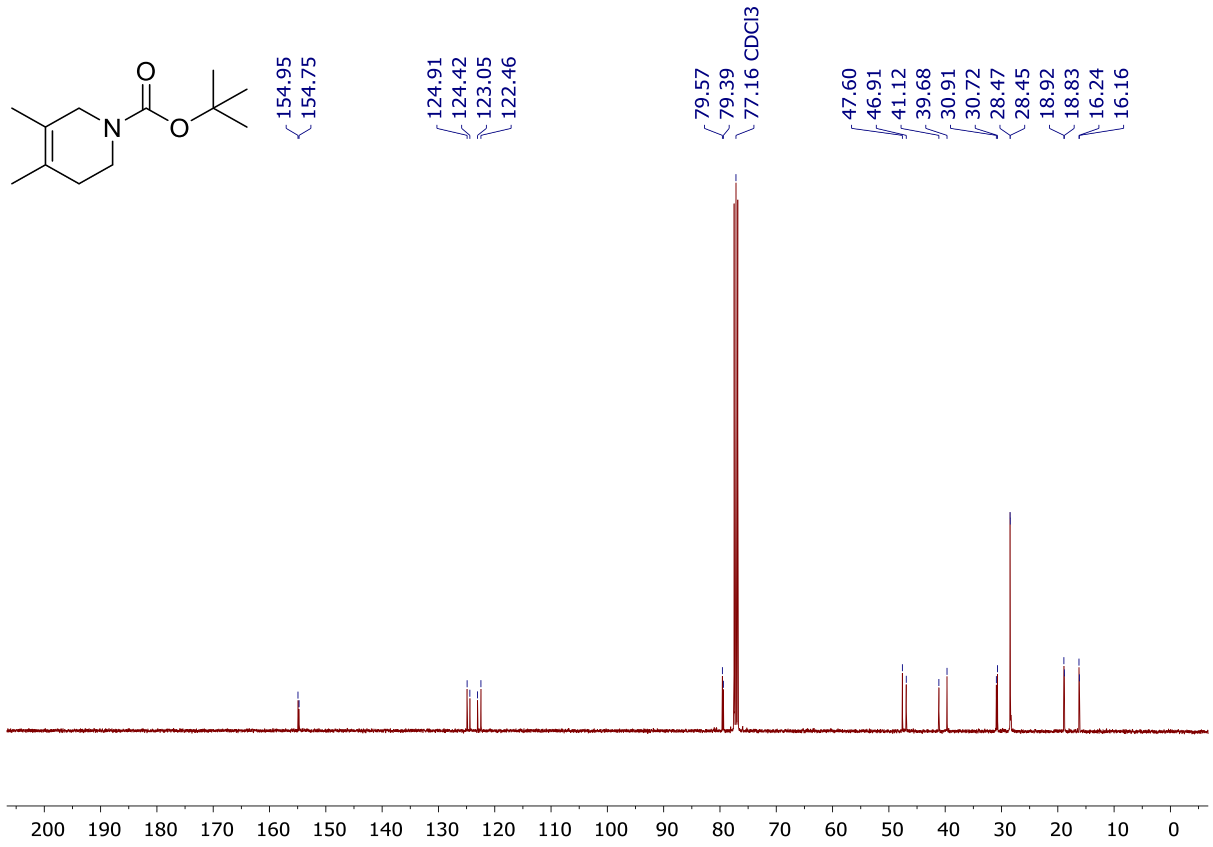

Figure S4: $^{13}C\{^{1}H\}$ NMR spectrum (101 MHz) of **1a'** in $CDCl_3$ after isolation via column chromatography. Spectrum recorded at −25°C.

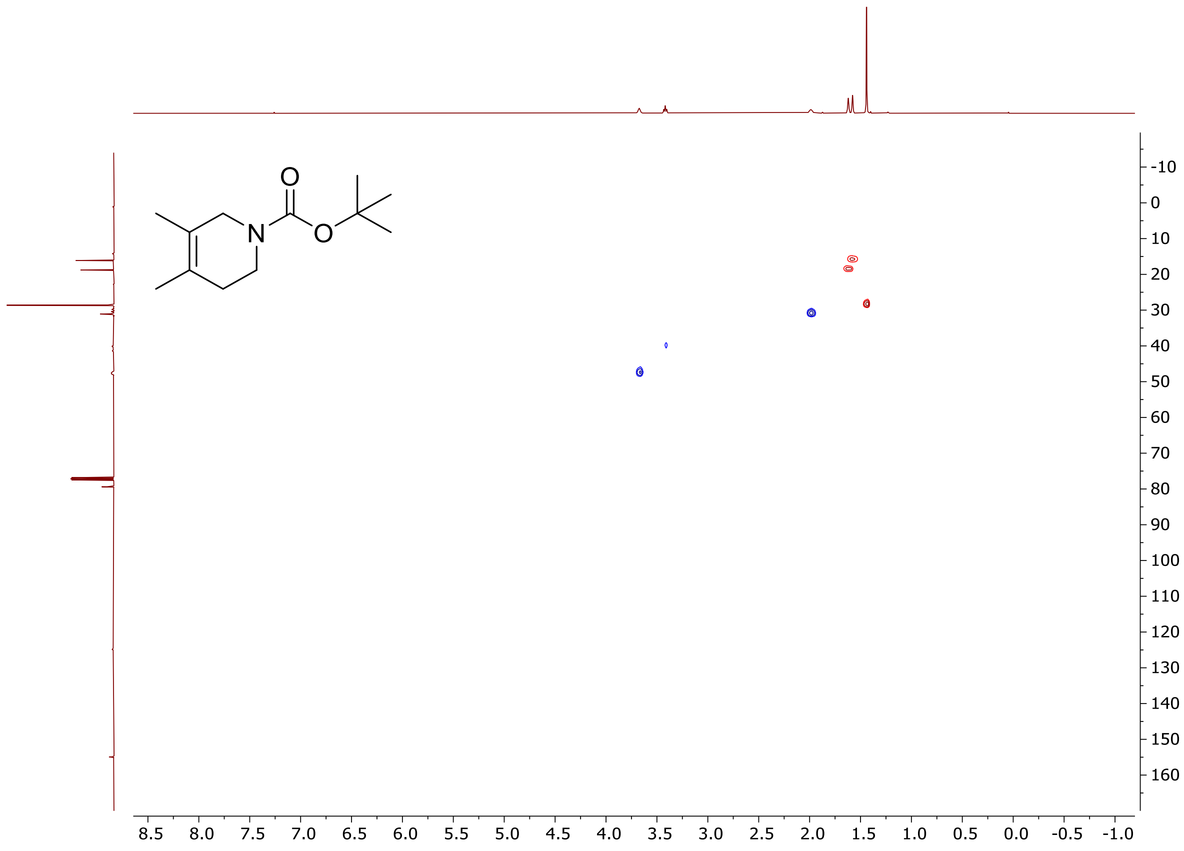

Figure S5: ^{1}*H-*^{13}*C HSQC* NMR spectrum (400 MHz) of **1a'** in $CDCl_3$ after isolation via column chromatography.

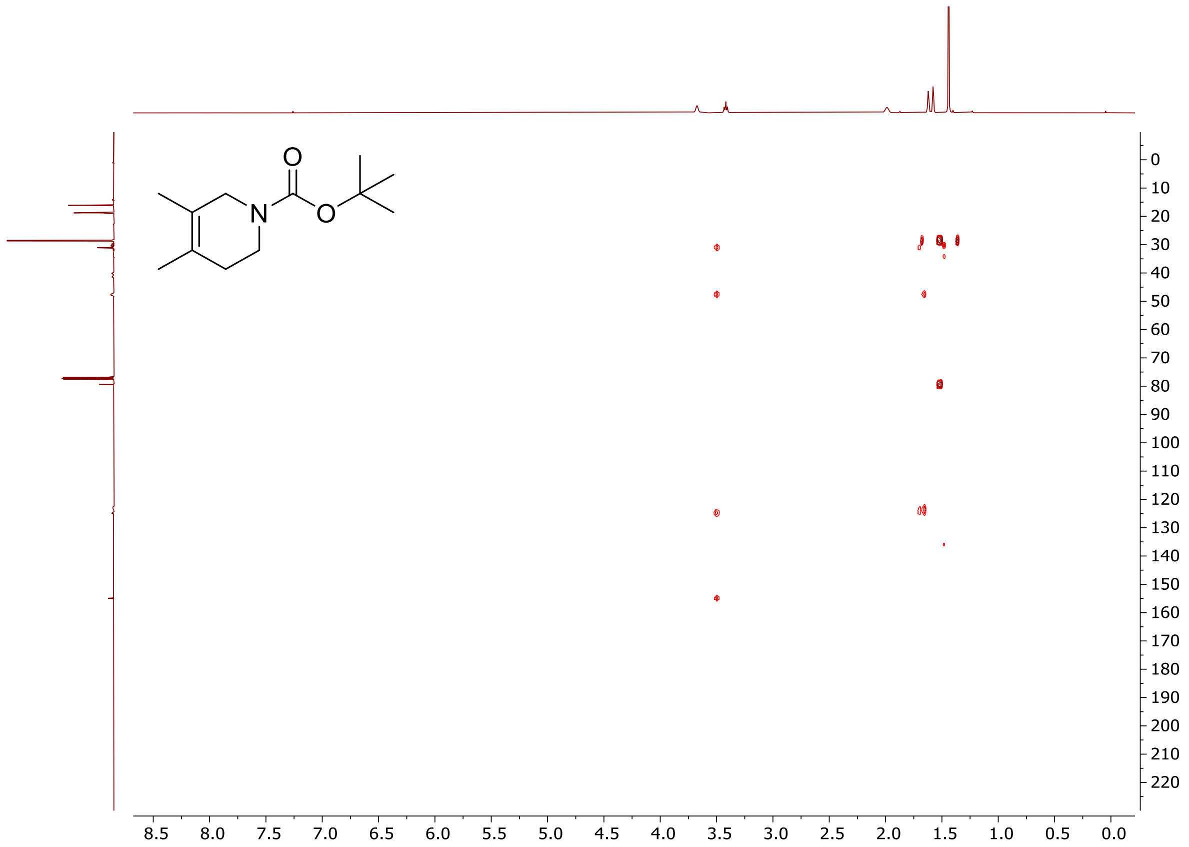

Figure S6: ^1H-^{13}C HMBC NMR spectrum (400 MHz) of **1a'** in $CDCl_3$ after isolation via column chromatography.

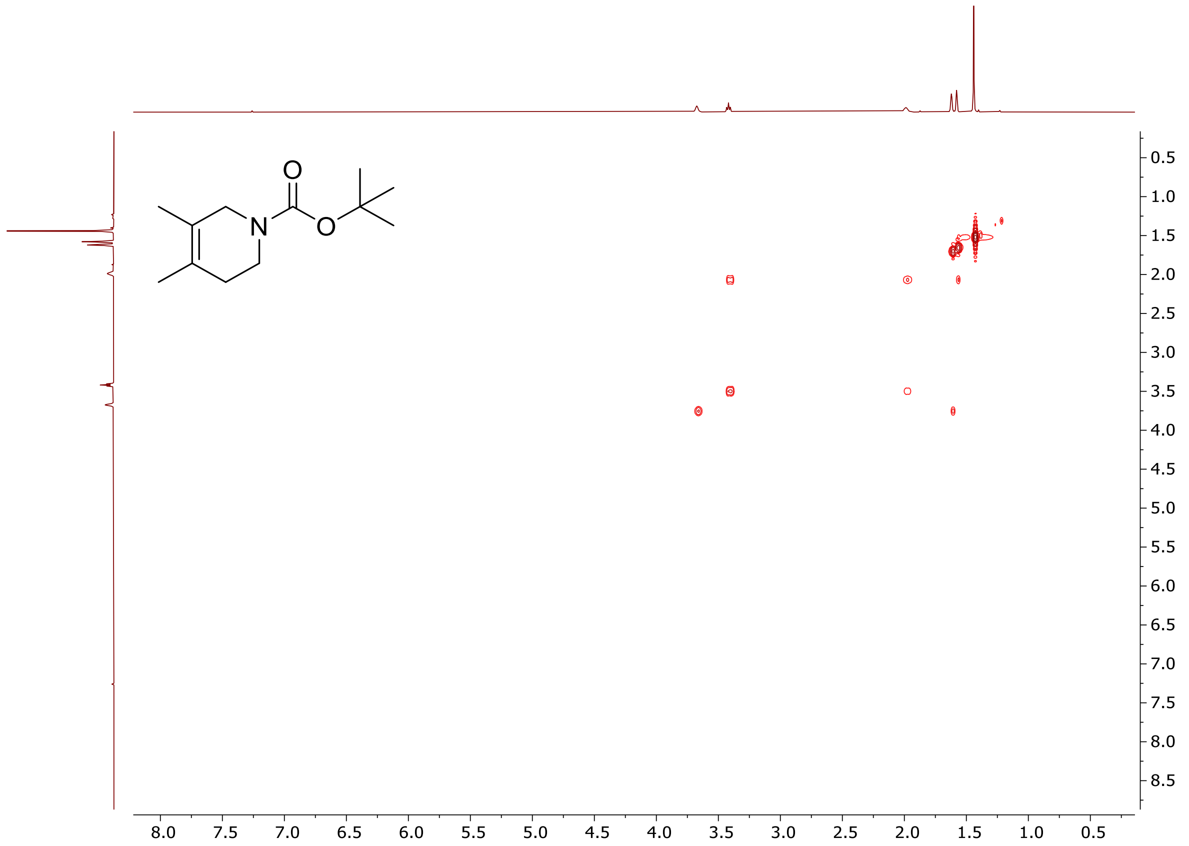

Figure S7: ^1H-^1H COSY NMR spectrum (400 MHz) of **1a'** in $CDCl_3$ after isolation via column chromatography.

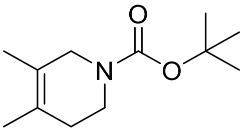

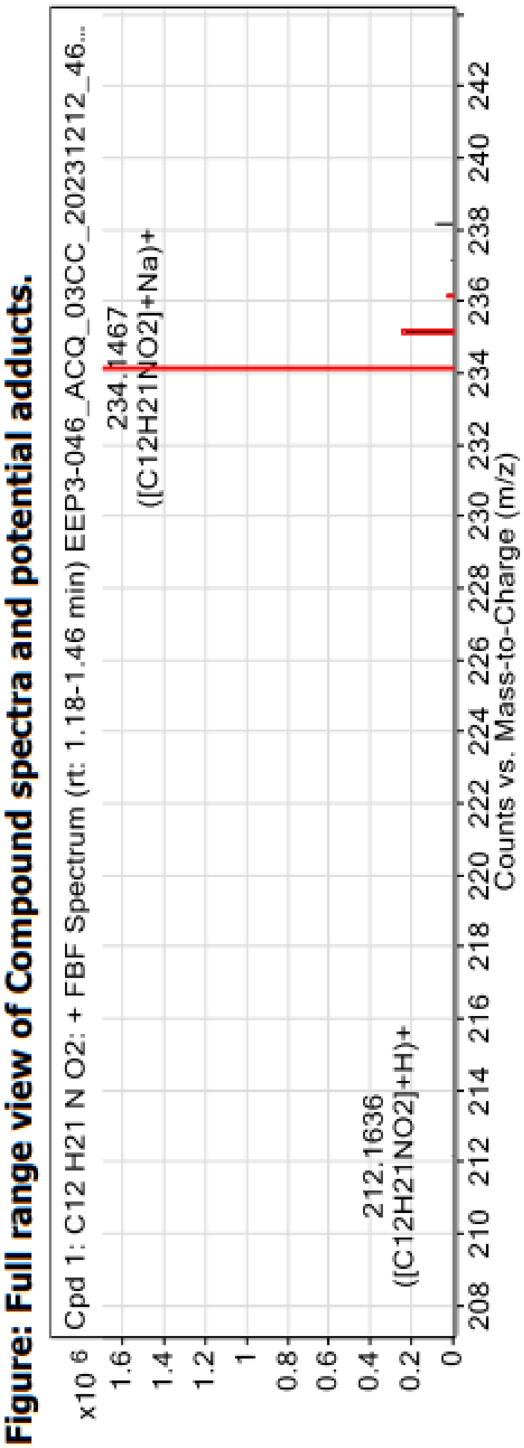

Figure S8: HRMS spectrum for compound **1a’**.

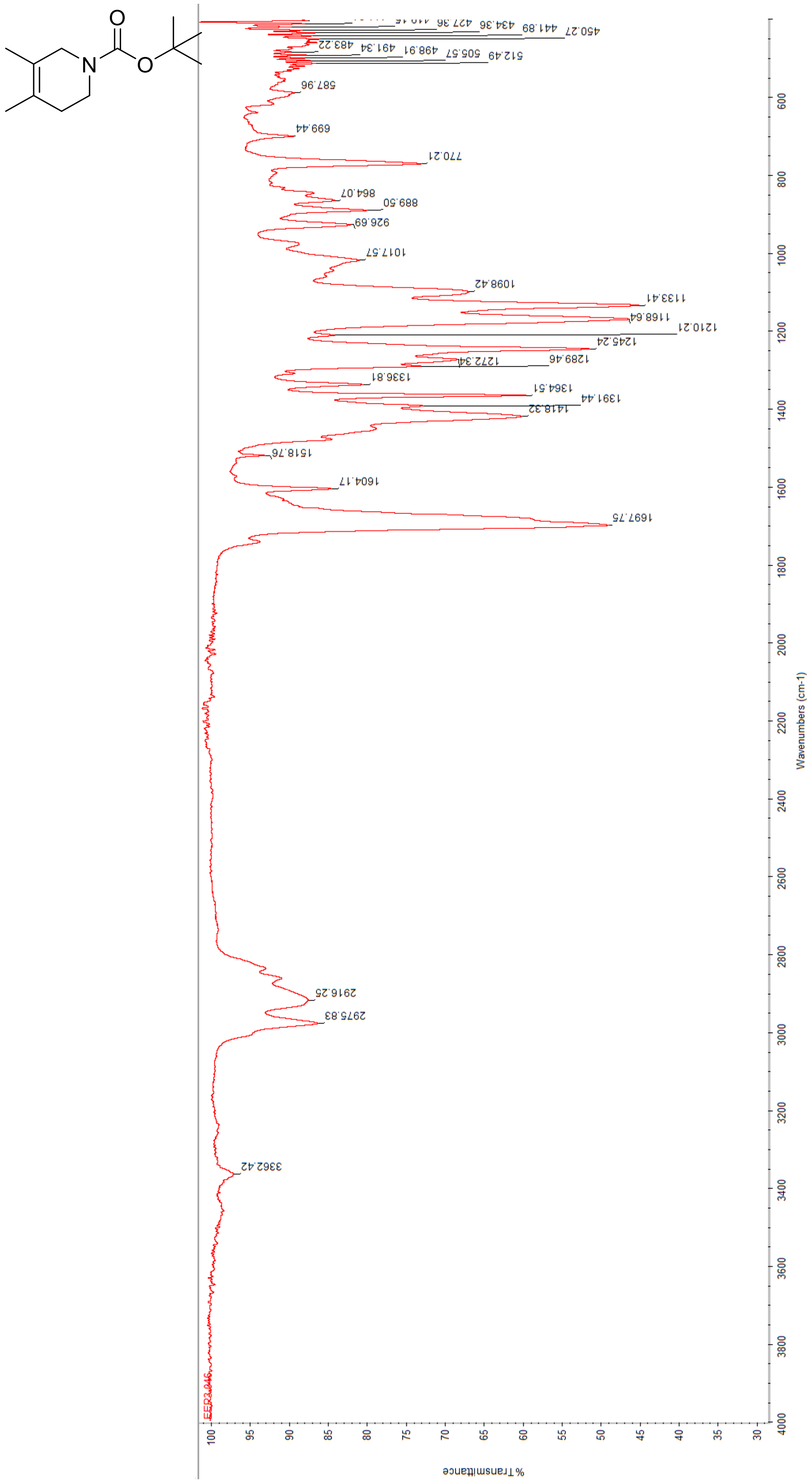

*Figure S9: IR spectrum for compound **1a'**.*

tert-butyl 4-methyl-3,6-dihydropyridine-1(2H)-carboxylate, **1b**

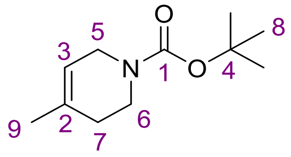

Following the general procedure and using isoprene (100.2 µL, 1 mmol per vial)

Yield: 0.164 g, 28% (colourless oil).

Analytic data is in accordance with those reported in literature.[2]

R_f = 0.70 (20% ethyl acetate / 80% hexane)

^{1}H NMR ($CDCl_3$, 400 MHz): δ 5.30 (s, 1H, C^3-H), 3.85 – 3.71 (*br.* m, 2H, C^5-H), 3.44 (t, *J* = 5.8 Hz, 2H, C^6-H), 1.98 (*br.* s, 2H, C^7-H), 1.67 (s, 3H, C^9-H), 1.43 (s, 9H, C^8-H).

^{13}C{^{1}H} NMR ($CDCl_3$, 101 MHz) δ 155.0 (C^1), 132.8 (rotamers) (C^2), 118.2 (rotamers) (C^3), 79.4 (C^4), 43.5 (rotamers) (C^5), 41.1/39.8 (rotamers) (C^6), 23.0 (rotamers), 28.6, 23.3.

^{13}C{^{1}H} NMR ($CDCl_3$, 101 MHz, -25°C) δ 154.9/154.7 (C^1), 132.8/132.4 (C^2), 118.0/117.5 (C^3), 79.4/79.3 (C^4), 43.1/42.4 (C^5), 40.6/39.2 (C^6), 29.6/29.4 (C^7), 28.2 (C^8), 23.4 (C^9).

HRMS (ESI+) m/z: $[M + Na]^+$ Calcd for $C_{11}H_{20}NO_2Na^+$ 198.1489; Found 198.1486.

FT-IR (cm^{-1}): 2974.2, 2929.2, 1694.7, 1452.2, 1415.7, 1364.6, 1153.7 cm^{-1}.

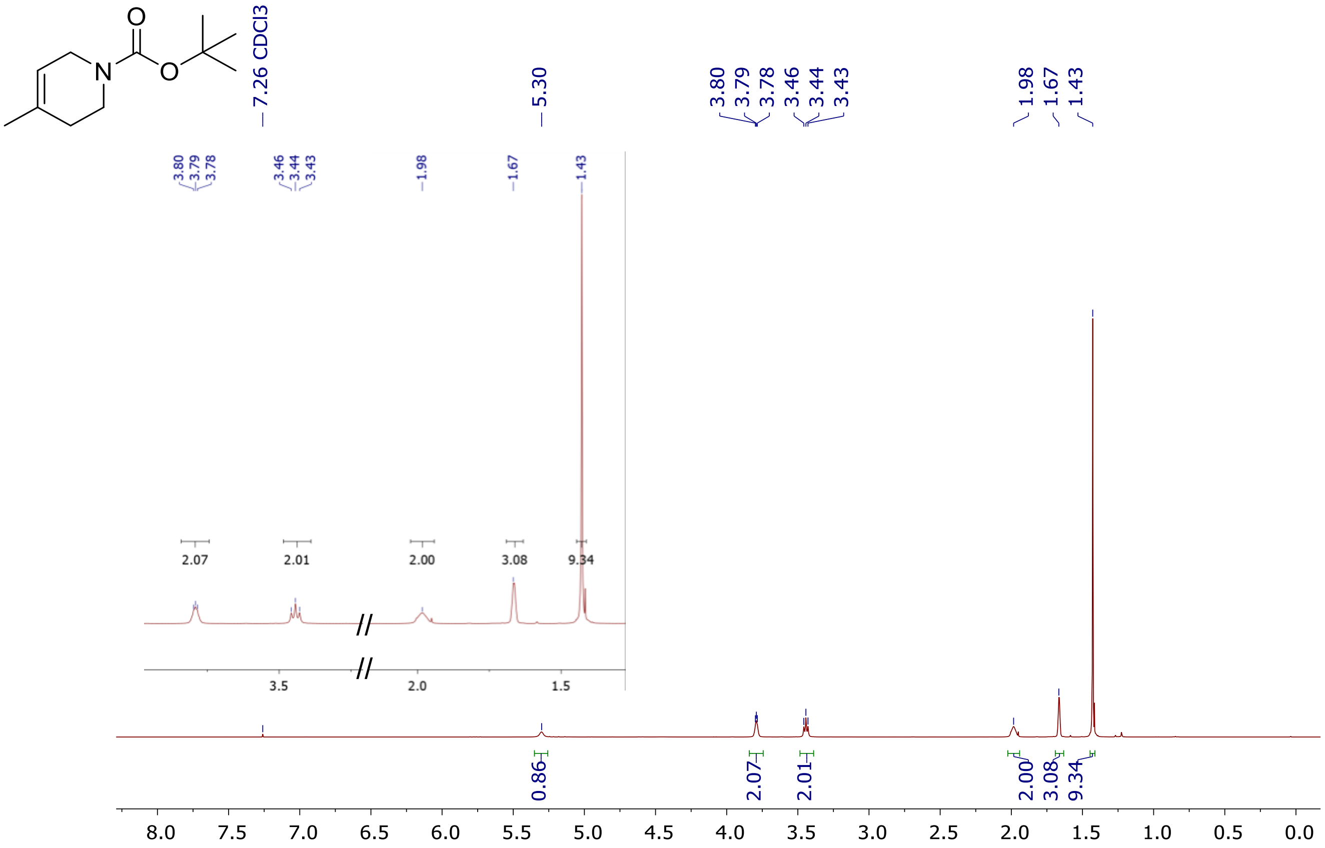

Figure S10: ^{1}H NMR spectrum (400 MHz) of **1b** in $CDCl_3$ after isolation via column chromatography. Inset shows zoom region.

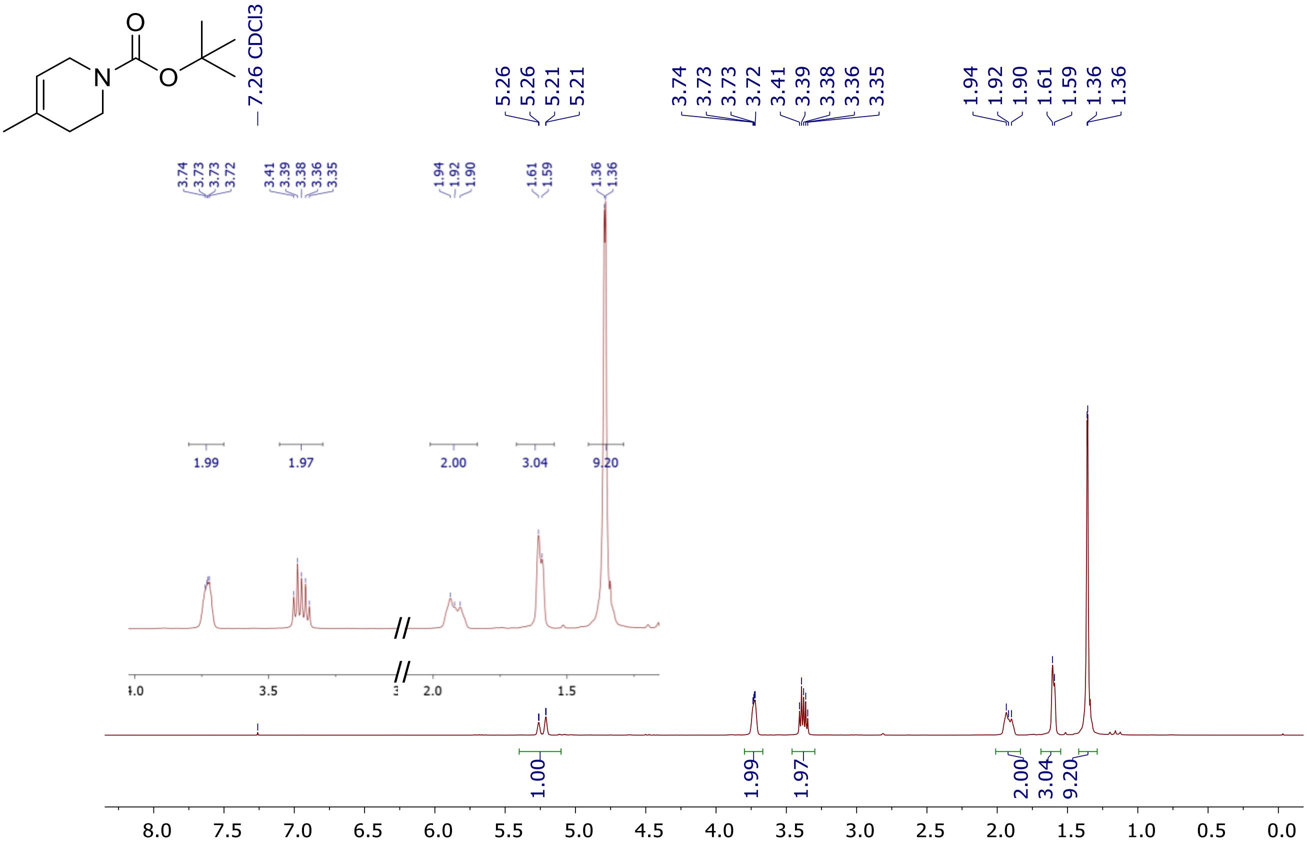

Figure S11: ^{1}H NMR spectrum (400 MHz) of **1b** in $CDCl_3$ after isolation via column chromatography. Spectrum recorded at −25°C. Inset shows zoom region.

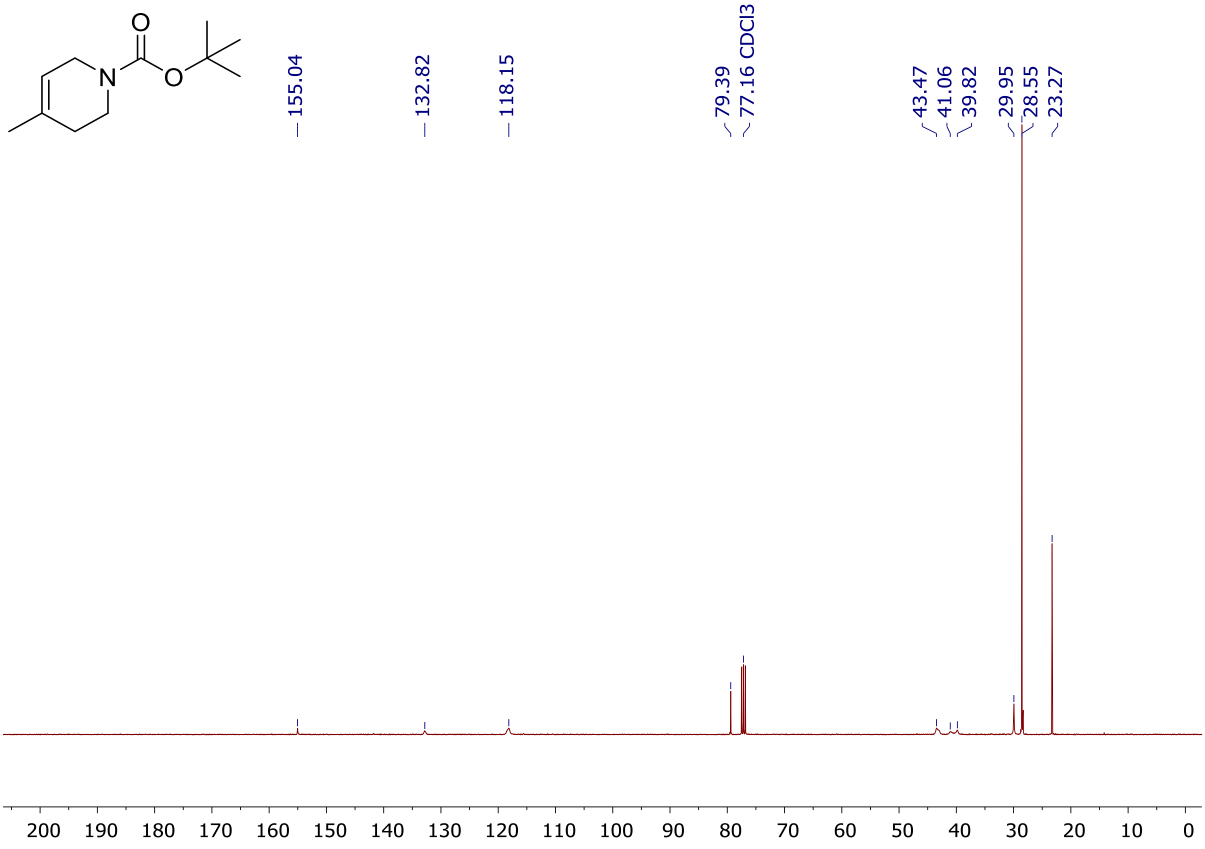

Figure S12: $^{13}C\{^{1}H\}$ NMR spectrum (101 MHz) of **1b** in $CDCl_3$ after isolation via column chromatography.

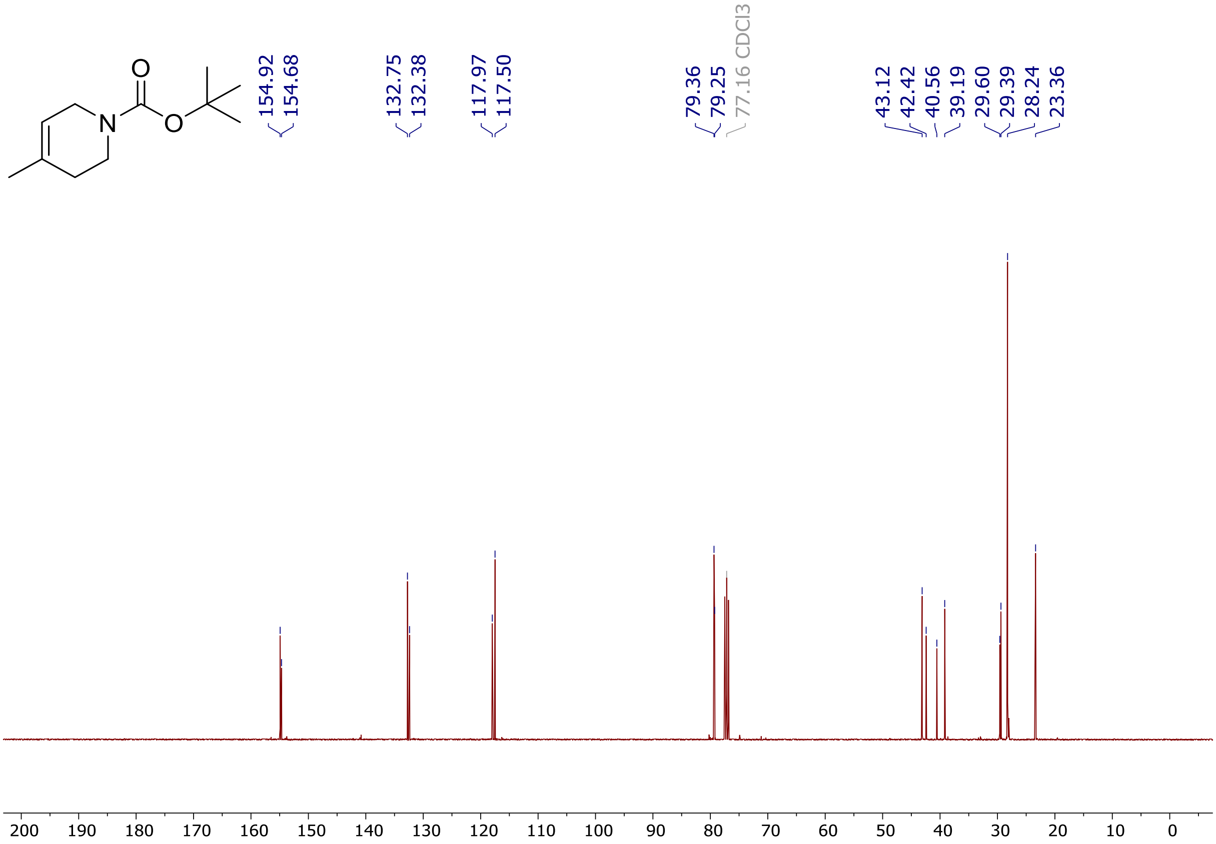

Figure S13: $^{13}C\{^{1}H\}$ NMR spectrum (101 MHz) of **1b** in $CDCl_3$ after isolation via column chromatography. Spectrum recorded at −25°C.

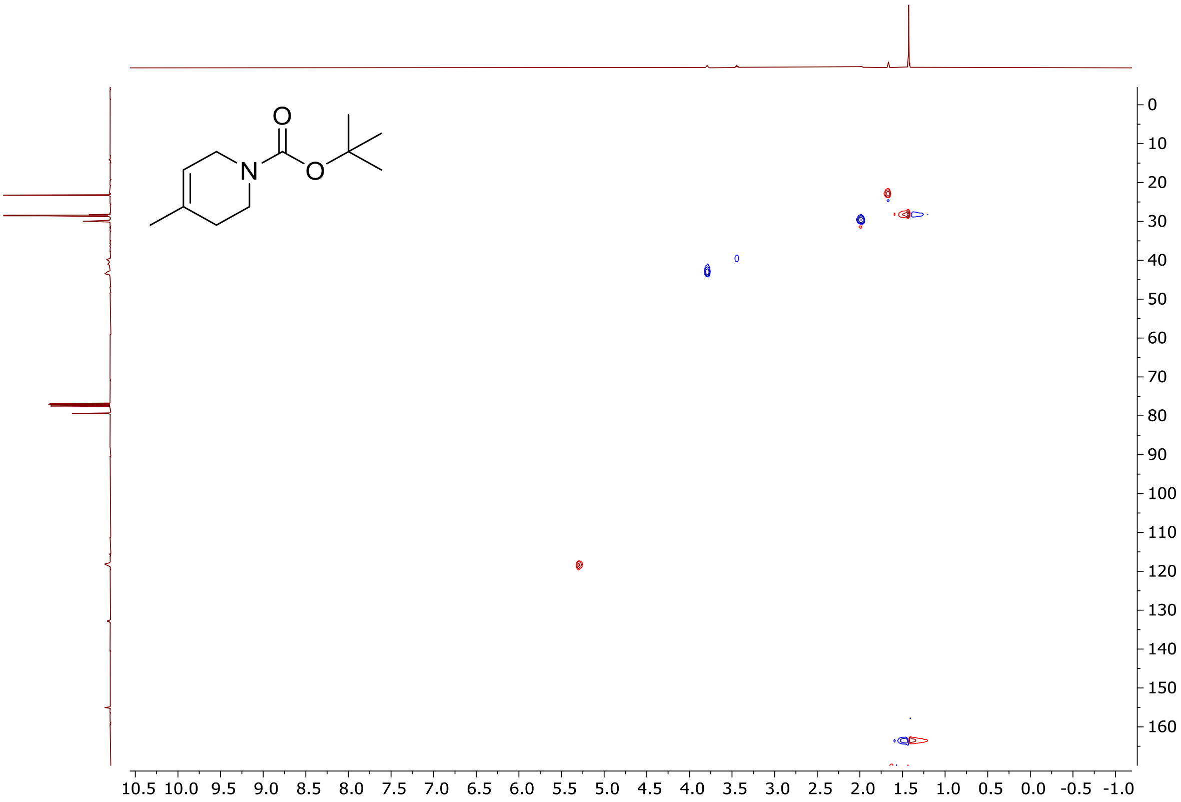

Figure S14: ^{1}H-^{13}C HSQC NMR spectrum (400 MHz) of **1b** in $CDCl_3$ after isolation via column chromatography.

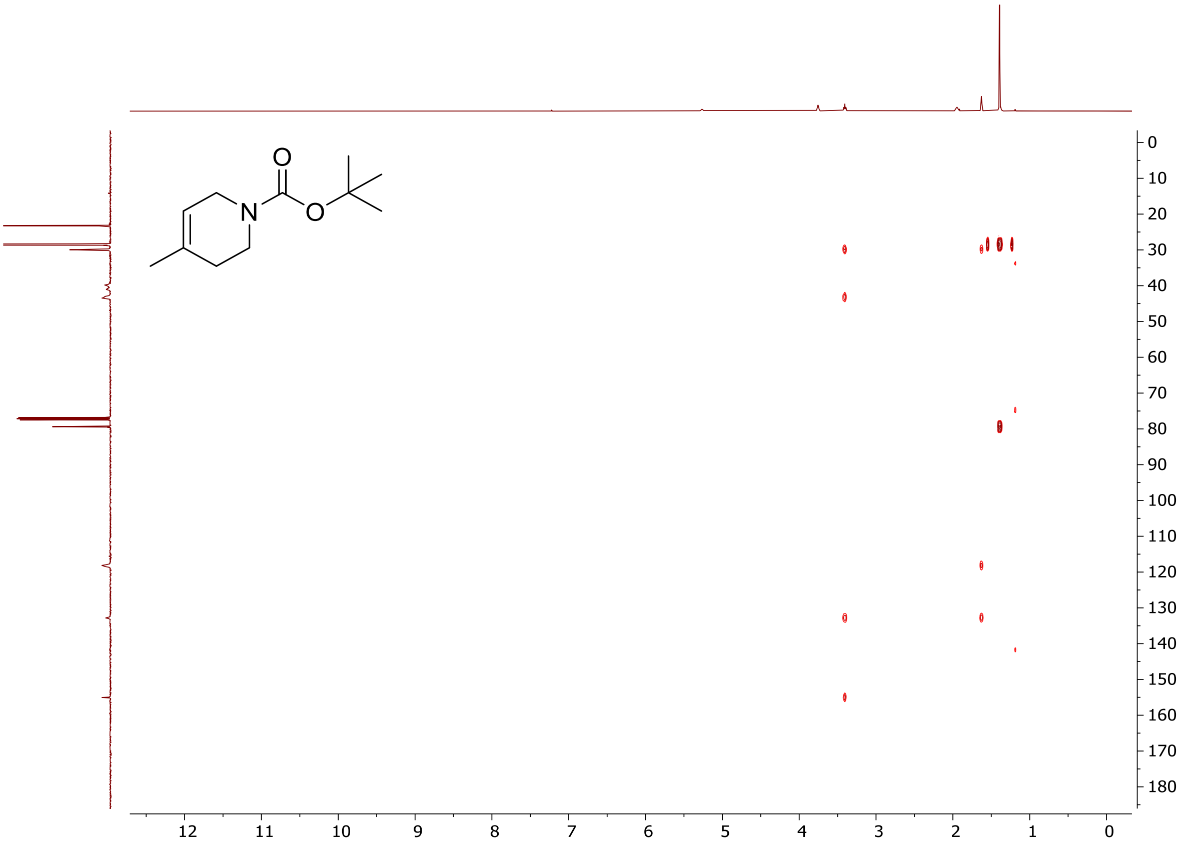

Figure S15: ^{1}H-^{13}C HMBC NMR spectrum (400 MHz) of **1b** in $CDCl_3$ after isolation via column chromatography.

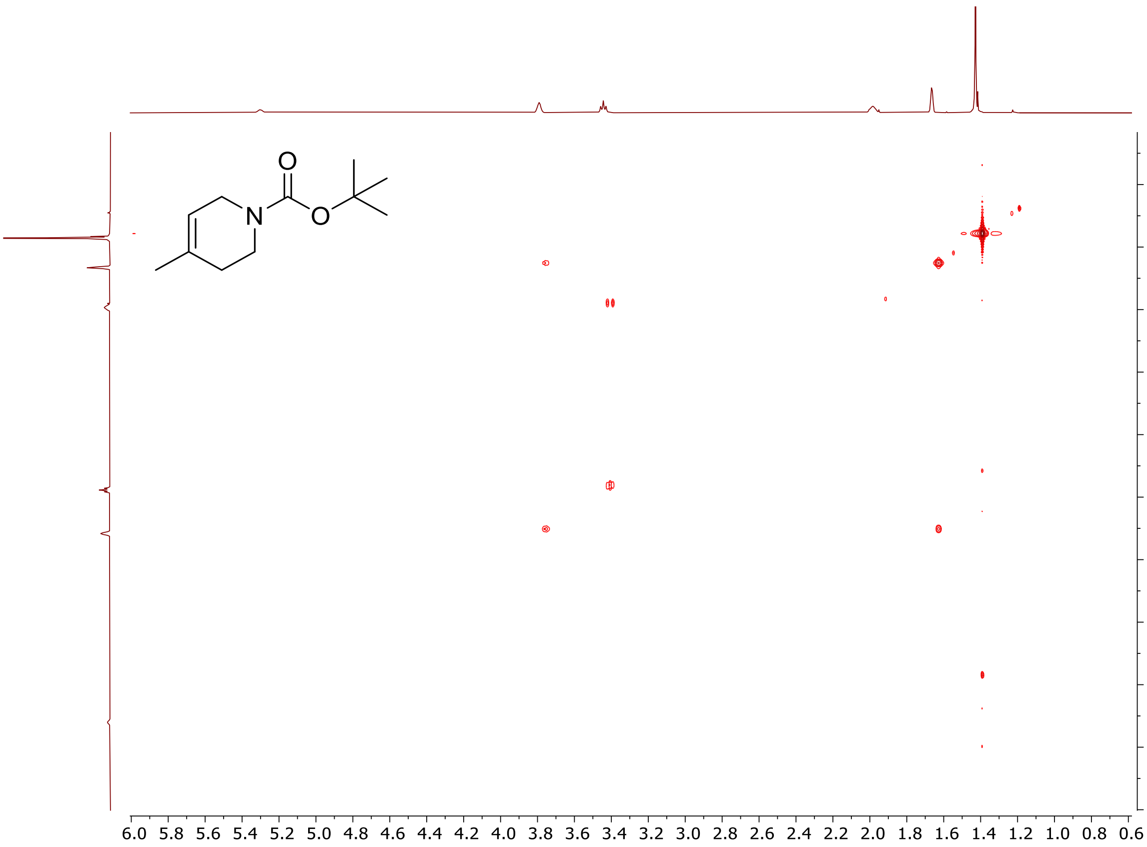

Figure S16: ^{1}H-^{1}H COSY NMR spectrum (400 MHz) of **1b** in $CDCl_3$ after isolation via column chromatography.

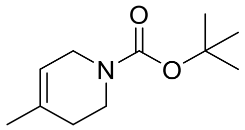

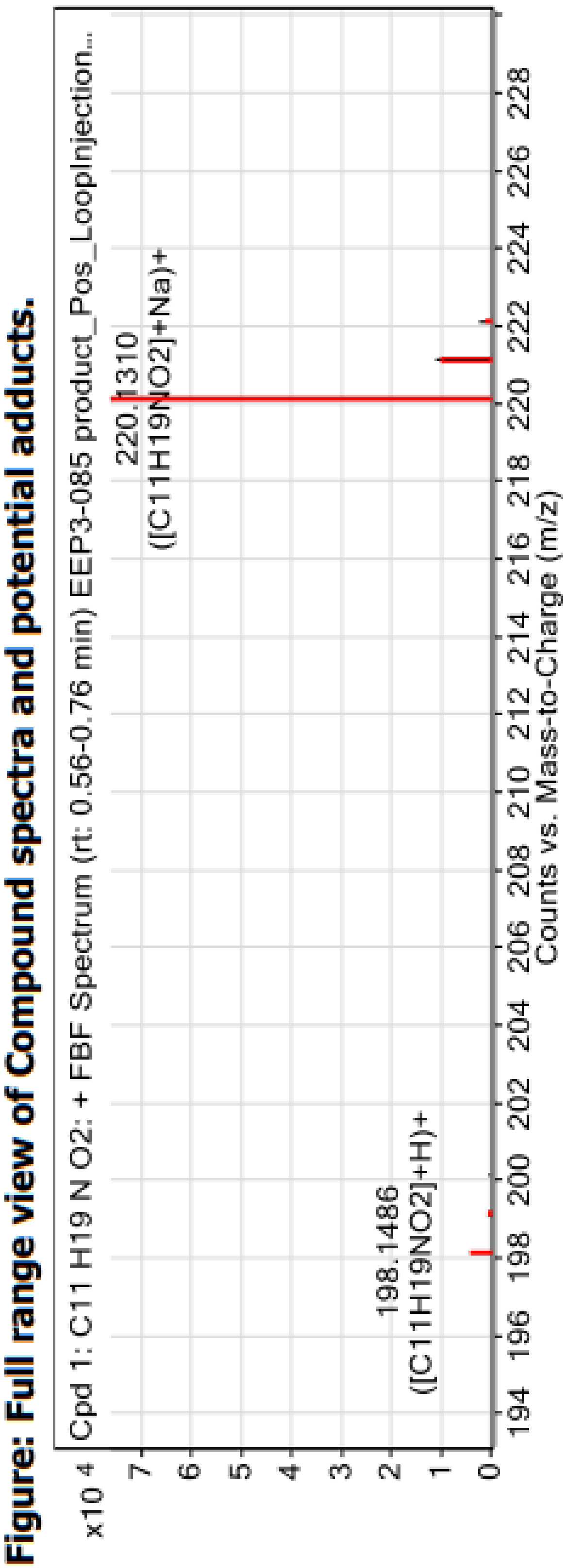

Figure S17: HRMS spectrum for compound ***1b****.*

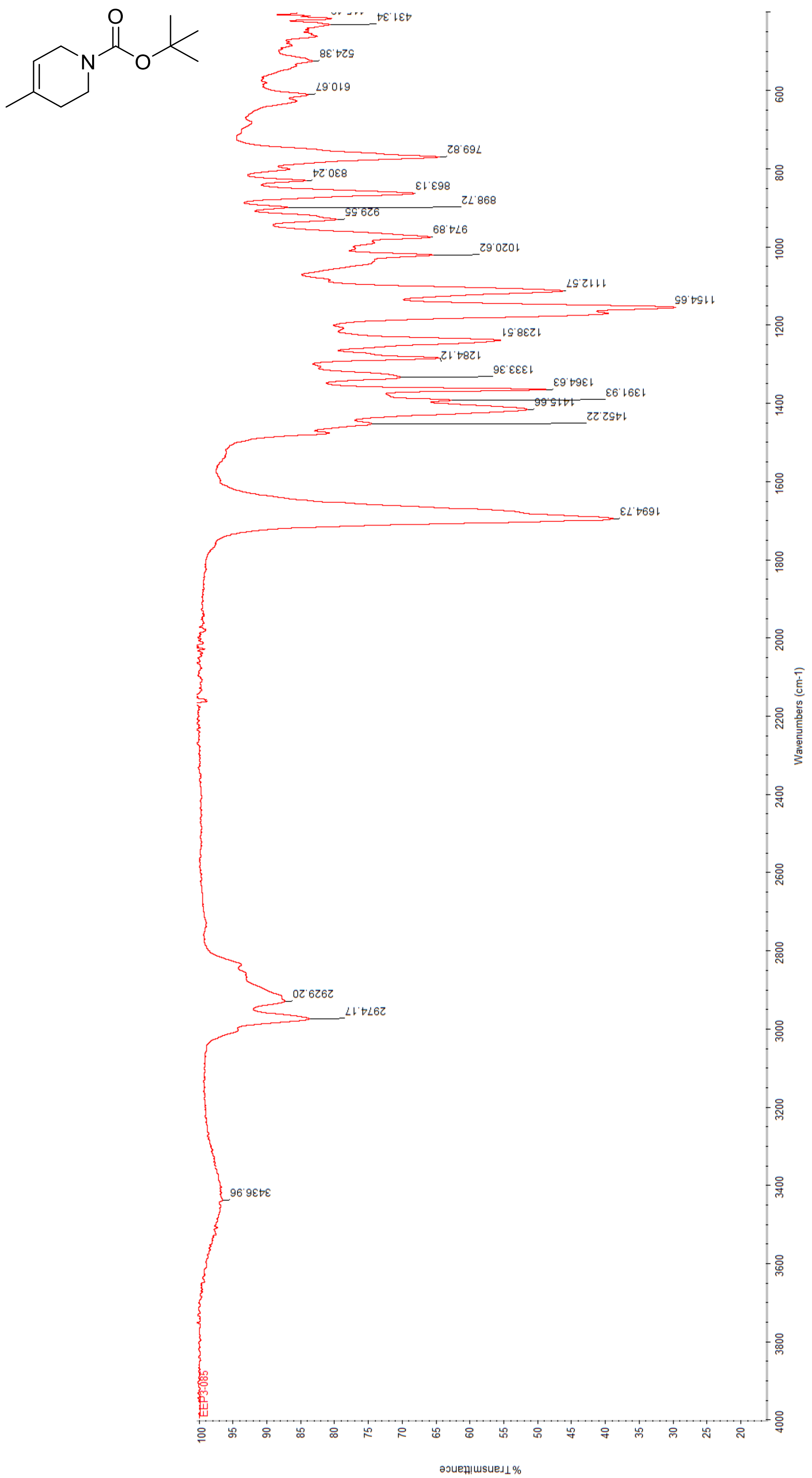

Figure S18: IR spectrum of Compound ***1b****.*

tert-butyl 4,6,6-trimethyl-3,6-dihydropyridine-1(2H)-carboxylate, **1c**

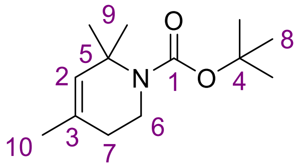

Following the general procedure and using 2,4-dimethyl-1,3 pentadiene (129.3 µL, 1 mmol per vial)

Yield: 0.049 g, 8% (colourless oil).

R_f = 0.80 (20% ethyl acetate / 80% hexane)

^{1}H NMR ($CDCl_3$, 400 MHz): δ 5.05 (d, *J* = 1.5 Hz, 1H, C^2-H), 3.48 (t, *J* = 5.6 Hz, 2H, C^6-H), 1.96 (t, *J* = 5.6 Hz, 2H, C^7-H), 1.69 (s, 3H, C^{10}-H), 1.48 (s, 9H, C^8-H), 1.39 (s, 6H, C^9-H).

^{13}C{^{1}H} NMR ($CDCl_3$, 101 MHz) δ 156.1 (C^1), 131.8 (C^2), 130.3 (C^3), 79.6 (C^4), 55.2 (C^5), 40.8 (C^6), 30.0 (C^7), 28.8 (C^8), 27.2 (C^9), 23.2 (C^{10}).

HRMS (ESI+) m/z: $[M + Na]^+$ Calcd for $C_{13}H_{24}NO_2^+$ 226.1802; Found 226.1795.

FT-IR (cm^{-1}): 2925.1, 2855.5, 1702.3, 1684.9, 1389.1, 1363.2, 1167.1 cm^{-1}.

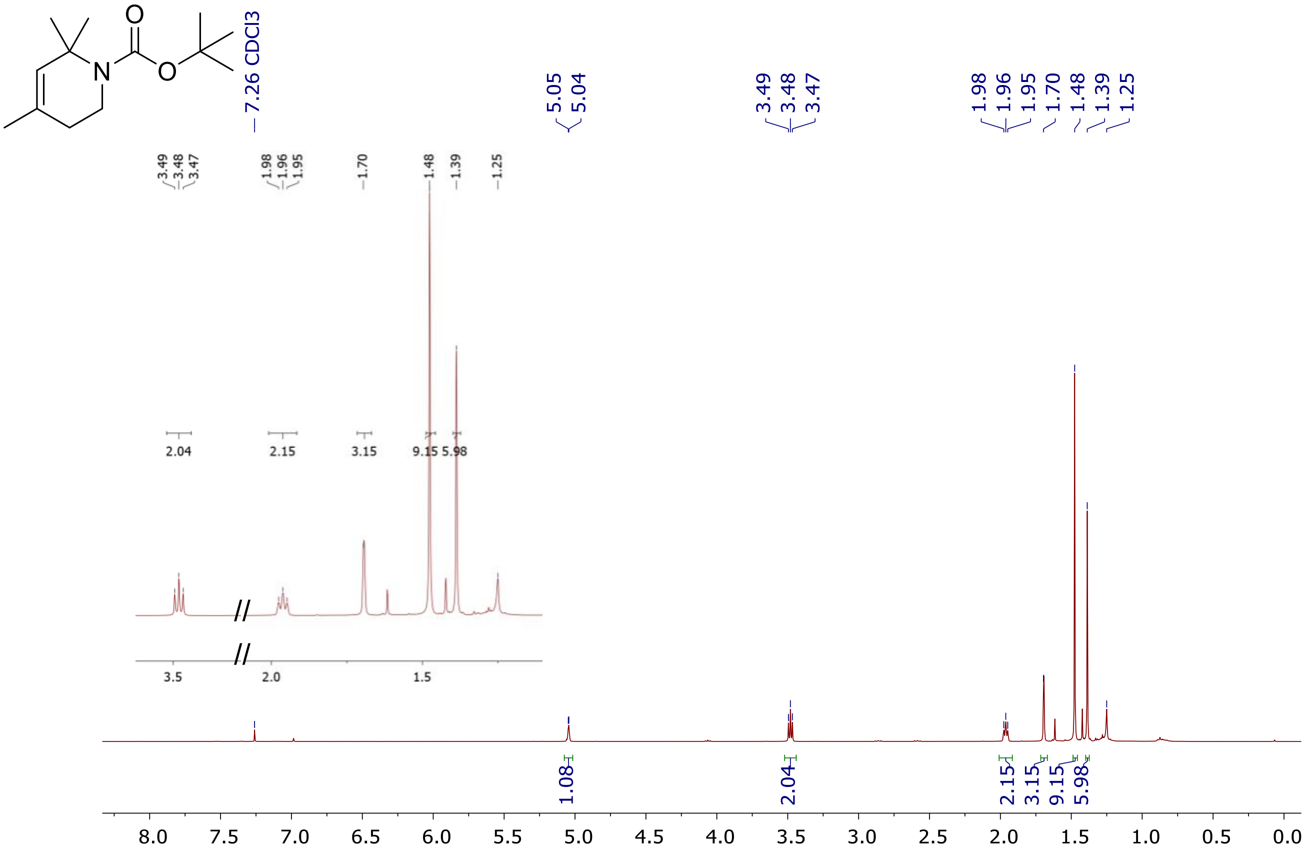

Figure S19: ^{1}H NMR spectrum (400 MHz) of **1c** in $CDCl_3$ after isolation via column chromatography. Inset shows zoom region.

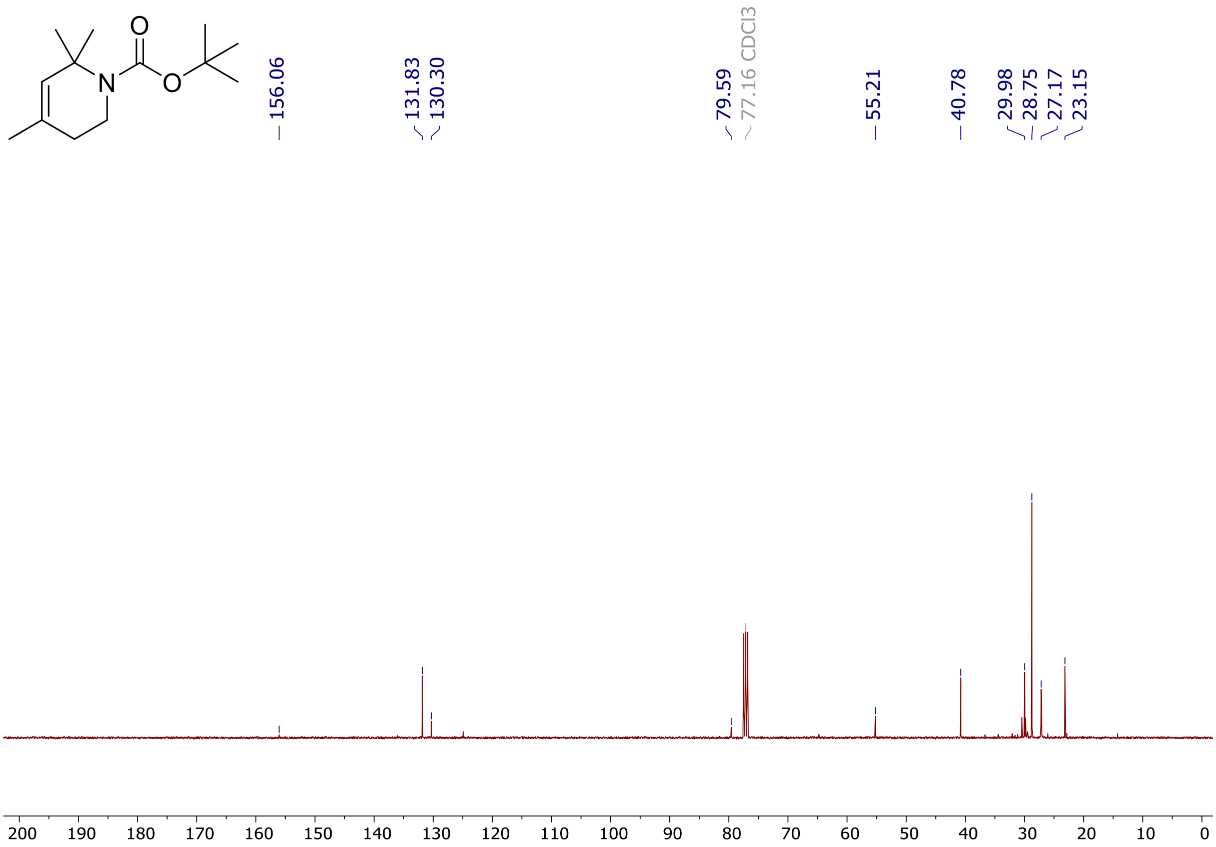

Figure S20: $^{13}C\{^{1}H\}$ NMR spectrum (101 MHz) of **1c** in $CDCl_3$ after isolation via column chromatography.

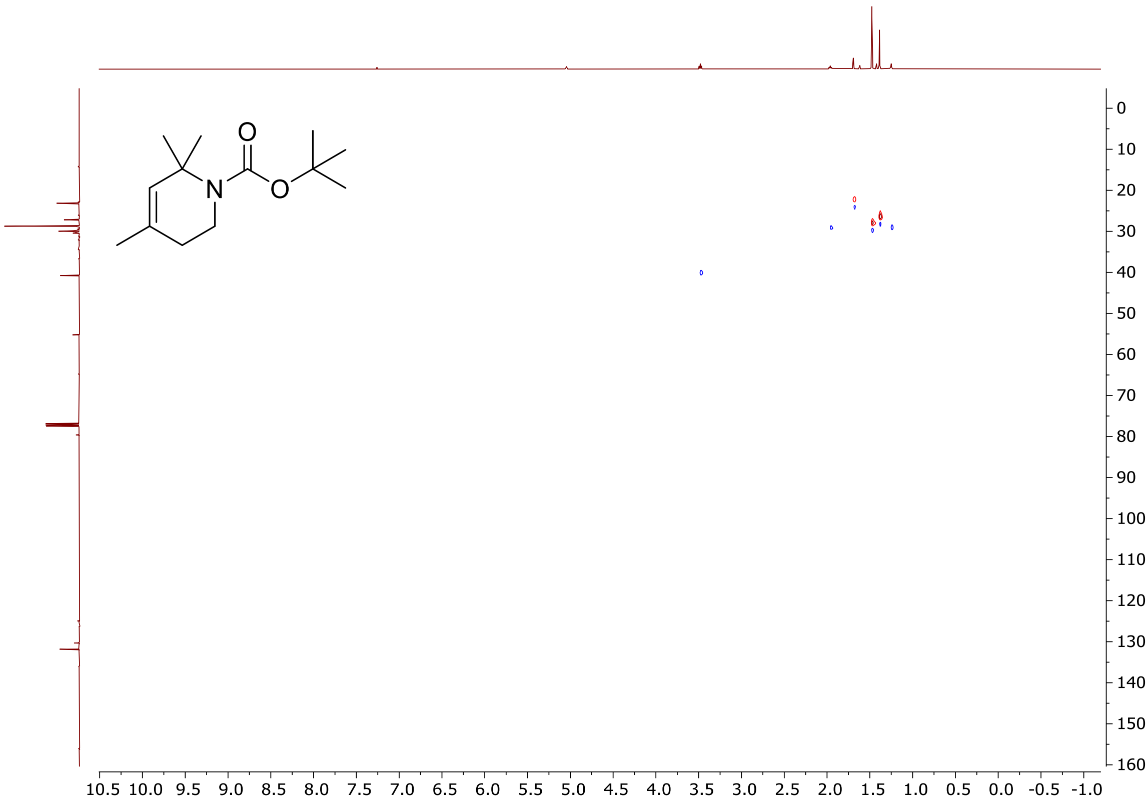

Figure S 21: *^{1}H-^{13}C HSQC* NMR spectrum (400 MHz) of **1c** in $CDCl_3$ after isolation via column chromatography.

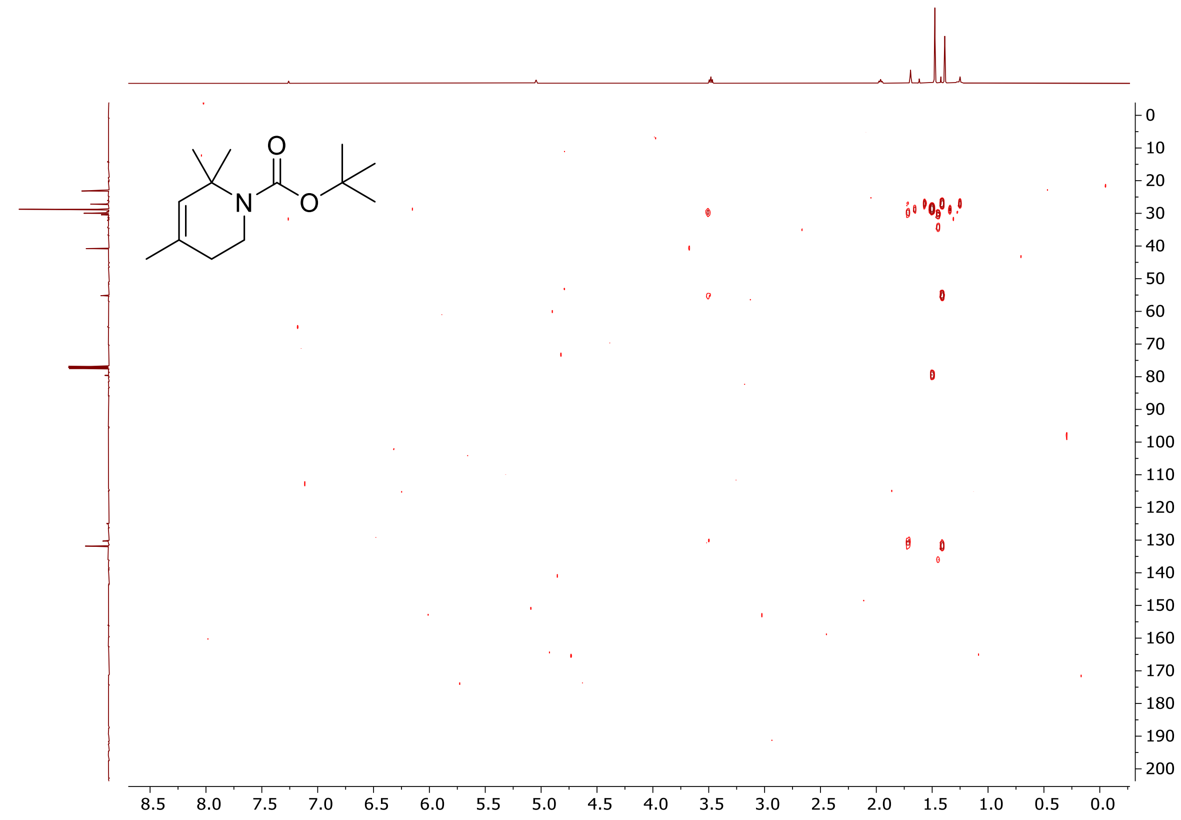

Figure S22: ^{1}H-^{13}C HMBC NMR spectrum (400 MHz) of **1c** in $CDCl_3$ after isolation via column chromatography.

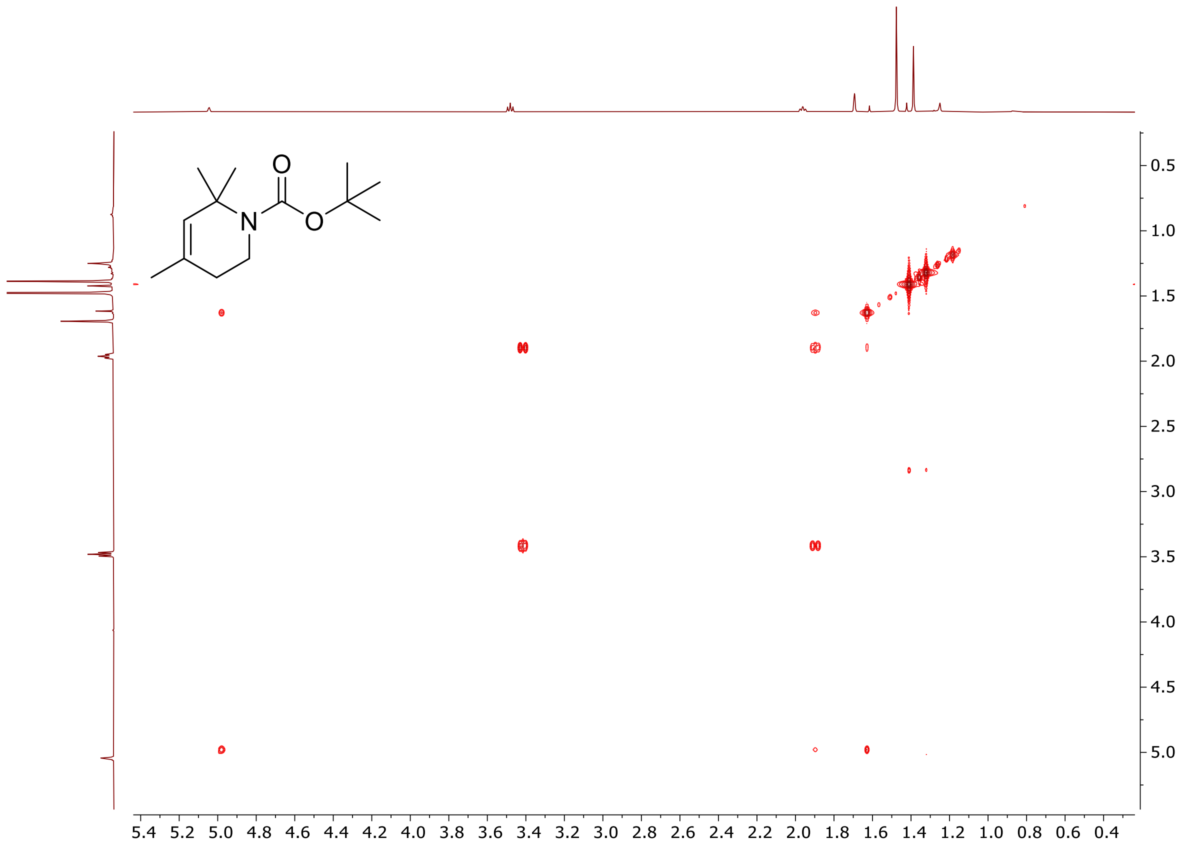

Figure S23: ^{1}H-^{1}H COSY NMR spectrum (400 MHz) of **1c** in $CDCl_3$ after isolation via column chromatography.

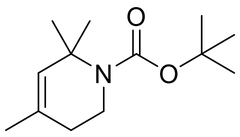

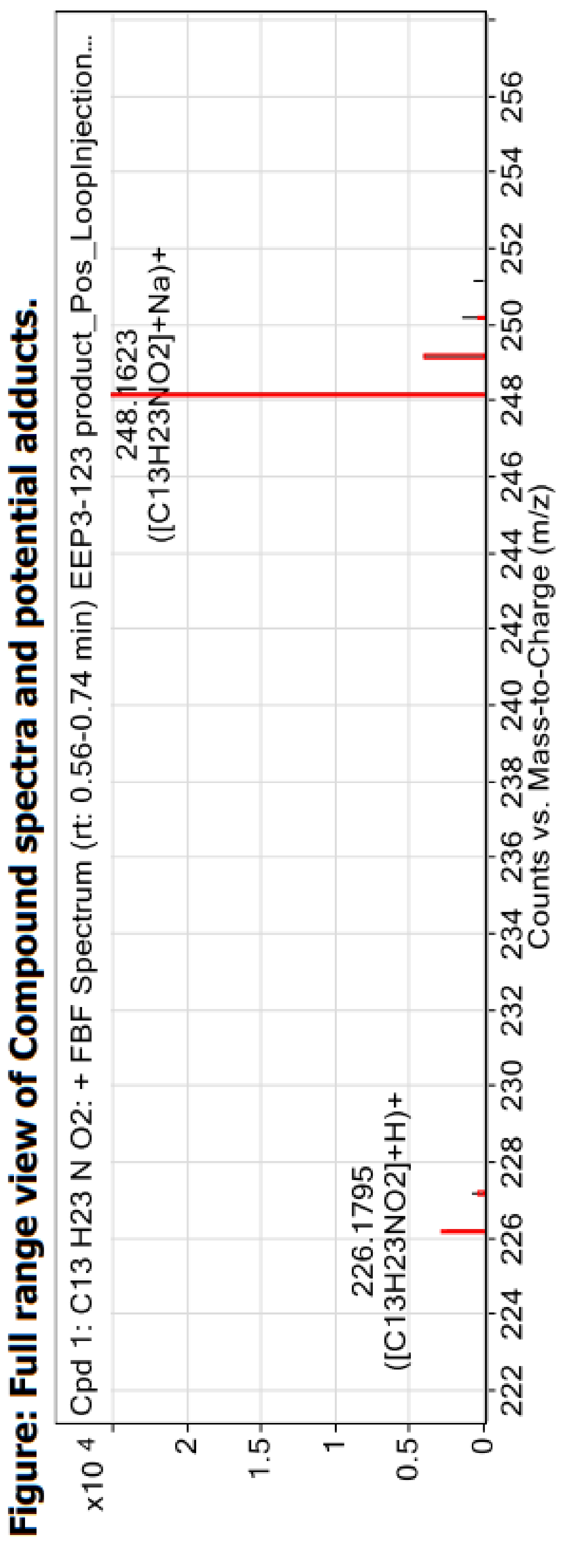

Figure S24: HRMS spectrum for compound ***1c****.*

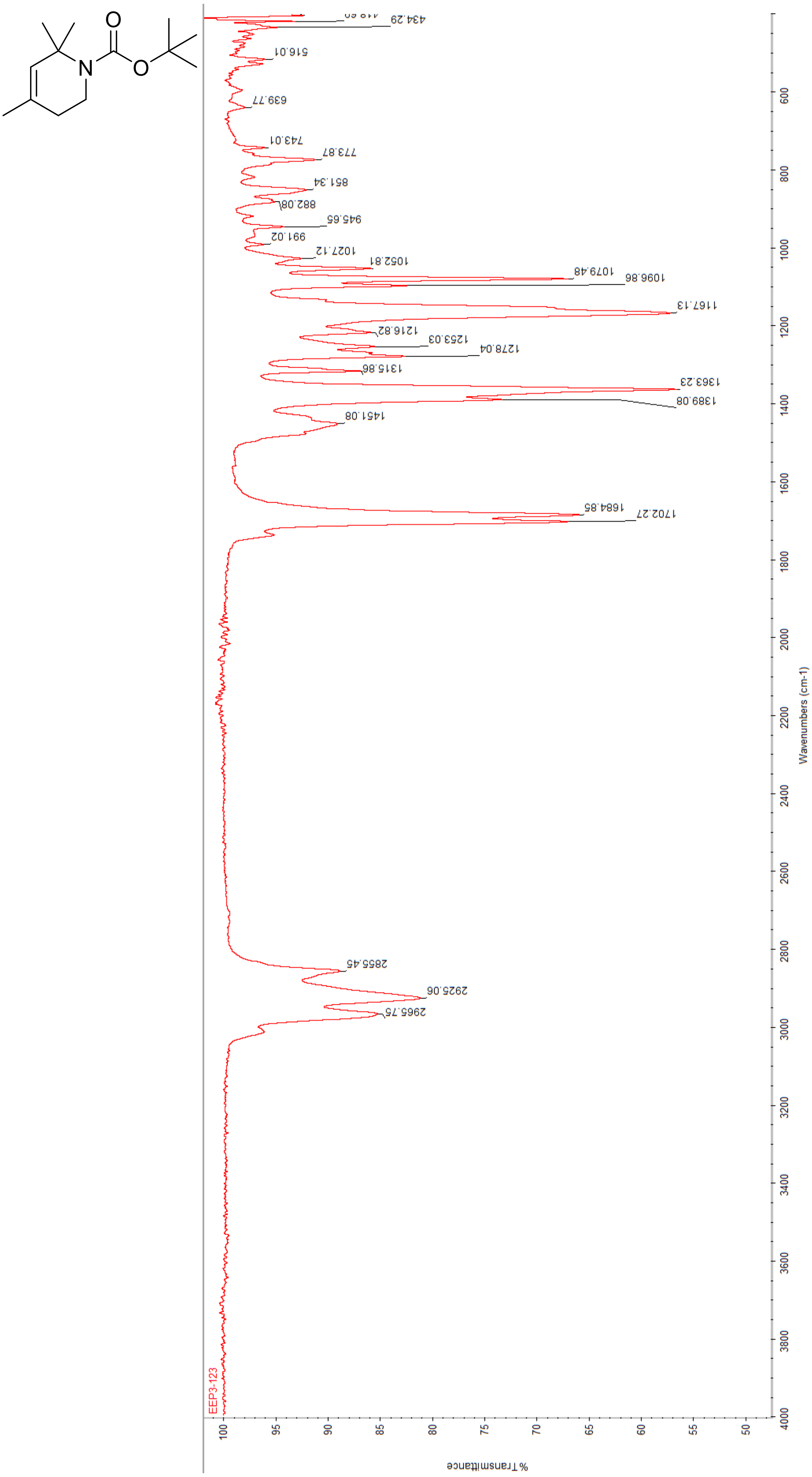

*Figure S25: IR spectrum of compound **1c**.*

tert-butyl 6-methyl-3,6-dihydropyridine-1(2H)-carboxylate, **1d**

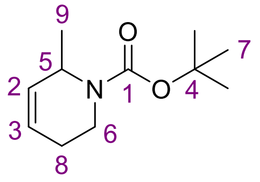

Following the general procedure and using trans-1,3 pentadiene (99.7 µL, 1 mmol per vial)

Yield: 0.042 g, 7% (colourless oil).

R_f = 0.67 (20% ethyl acetate / 80% hexane)

^{1}H NMR ($CDCl_3$, 400 MHz): δ 5.83 – 5.73 (*br.* m, 1H, C^3-H), 5.58 (*br.* d, *J* = 10.1 Hz, 1H, C^2-H), 4.40 (s, 1H, C^6-H), 4.07 (s, 1H, C^6-H), 2.82 (s, 1H, C^5-H), 2.23 – 2.06 (*br.* m, 1H, C^8-H), 1.93 (d, *J* = 16.7 Hz, 1H, C^8-H), 1.46 (s, 9H, C^7-H), 1.16 (d, *J* = 6.8 Hz, 3H, C^9-H).

^{13}C{^{1}H} NMR ($CDCl_3$, 101 MHz) δ 154.6 (C^1), 130.2 (C^2), 124.7 (C^3), 79.4 (C^4), 48.1 (C^5), 35.7 (C^6), 28.7 (C^7), 25.2 (C^8), 18.9 (C^9).

^{13}C{^{1}H} NMR ($CDCl_3$, 101 MHz, -25°C) δ 154.7/154.2 (C^1), 130.4/129.7 (C^2), 124.8/124.4 (C^3), 79.5/79.4 (C^4), 48.2/47.3 (C^5), 36.8/35.3 (C^6), 28.5 (C^7), 25.2/25.0 (C^8), 18.9/18.6 (C^9).

HRMS (ESI+) m/z: $[M + Na]^+$ Calcd for $C_{11}H_{19}NO_2Na^+$ 220.1314; Found 220.1308.

FT-IR (cm^{-1}): 2974.0, 2928.3, 1692.2, 1413.8, 1363.9, 1170.3 cm^{-1}.

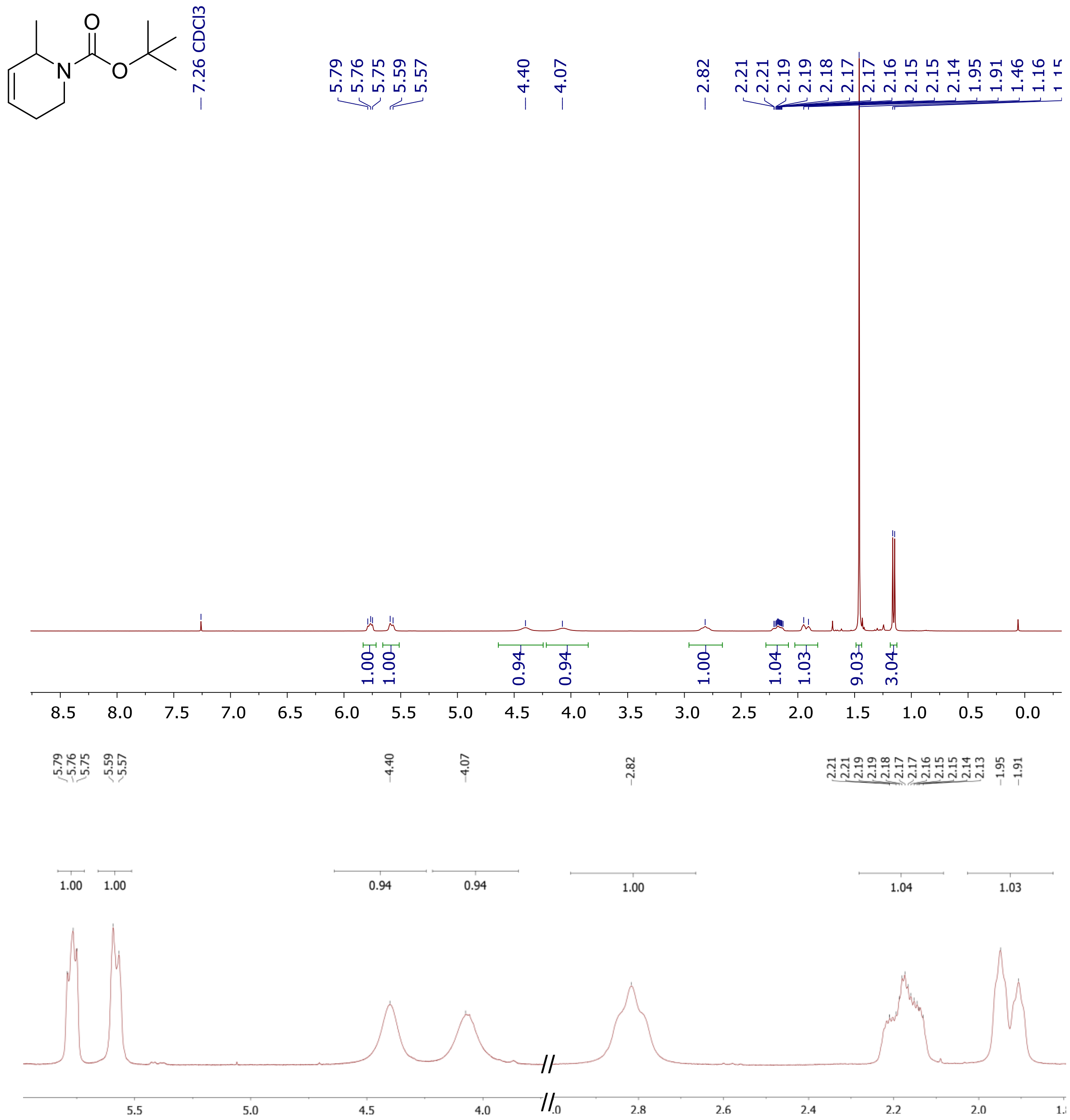

Figure S26: ^{1}H NMR spectrum (400 MHz) of **1d** in $CDCl_3$ after isolation via column chromatography. Full spectral range (top spectrum) with zoom regions shown below.

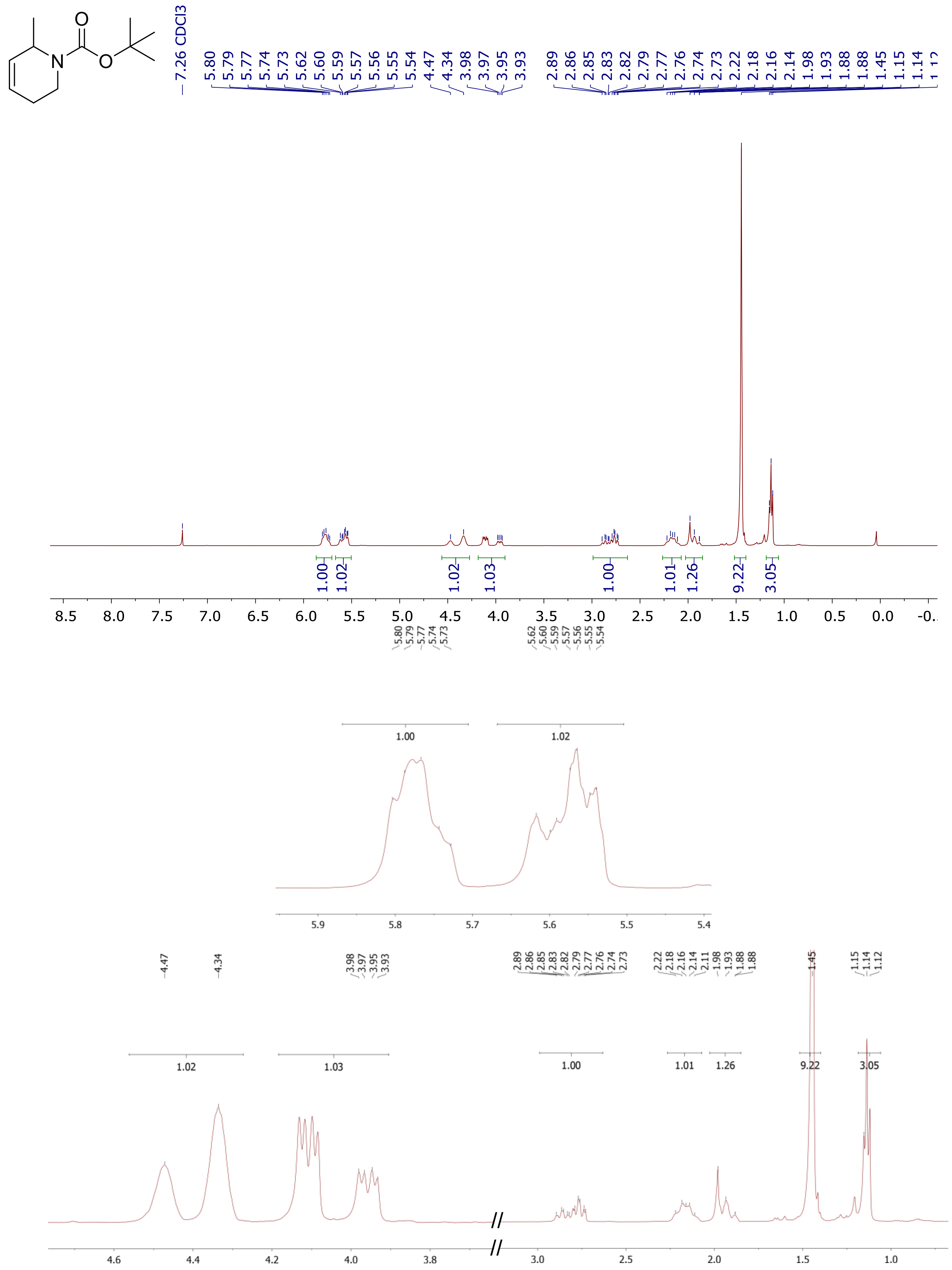

Figure S27: ^{1}H NMR spectrum (400 MHz) of **1d** in $CDCl_3$ after isolation via column chromatography. Spectrum recorded at −25°C. Full spectral range (top spectrum) with zoom regions shown below.

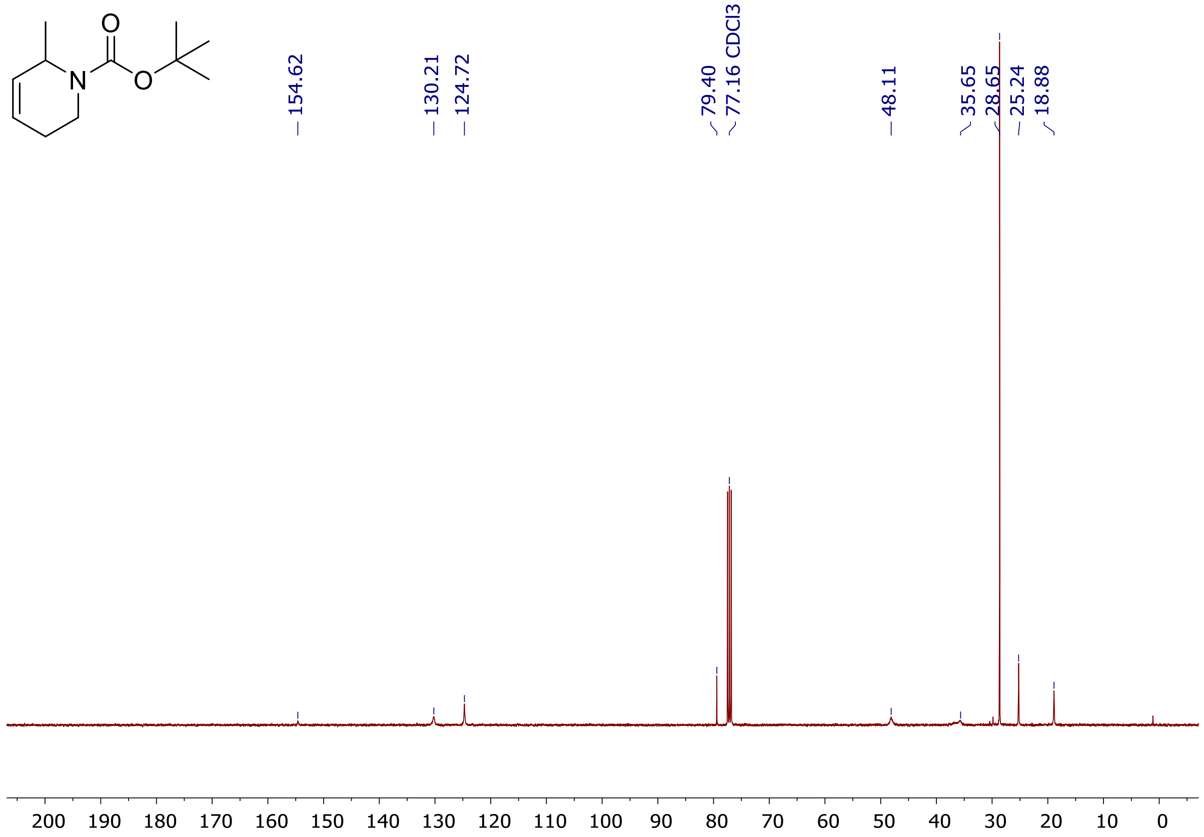

Figure S28: $^{13}C\{^{1}H\}$ NMR spectrum (101 MHz) of **1d** in $CDCl_3$ after isolation via column chromatography.

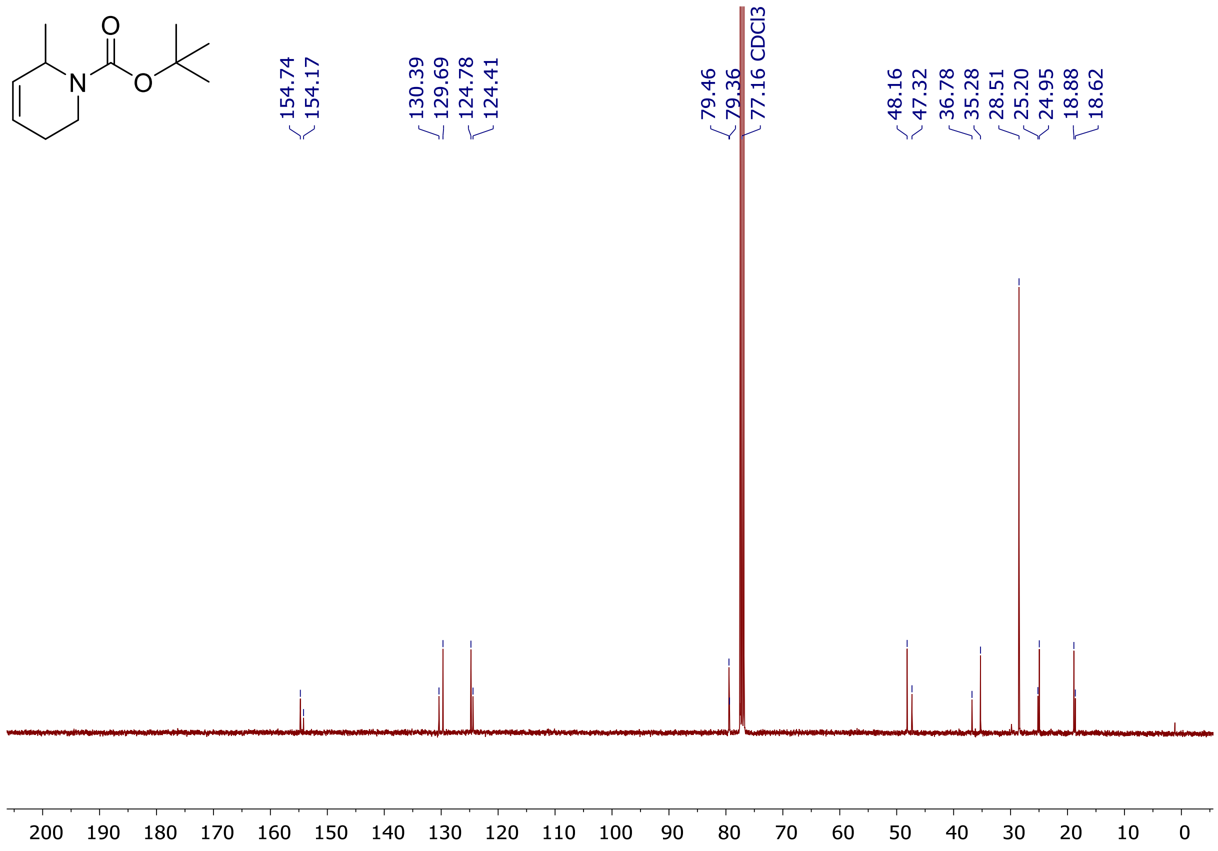

Figure S29: $^{13}C\{^{1}H\}$ NMR spectrum (101 MHz) of **1d** in $CDCl_3$ after isolation via column chromatography. Spectrum recorded at −25°C.

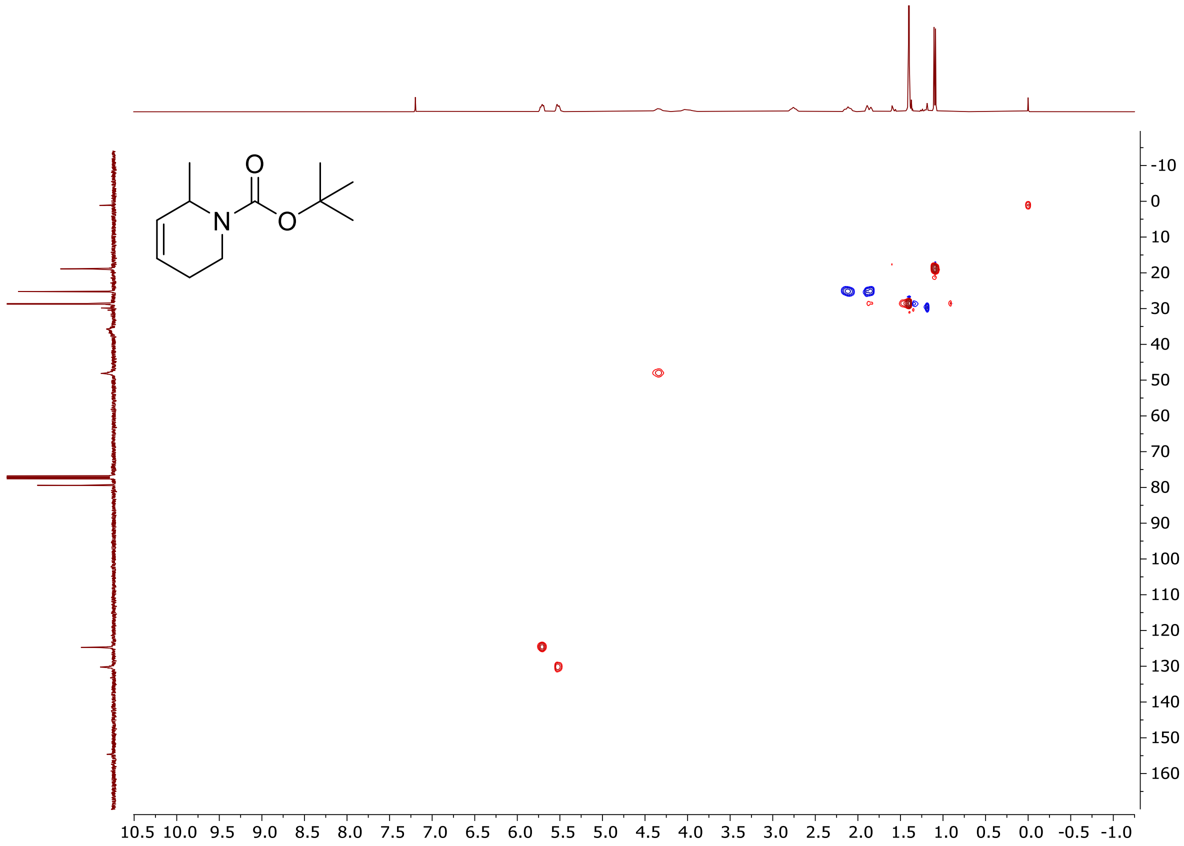

Figure S30: ^{1}H-^{13}C *HSQC* NMR spectrum (400 MHz) of **1d** in $CDCl_3$ after isolation via column chromatography.

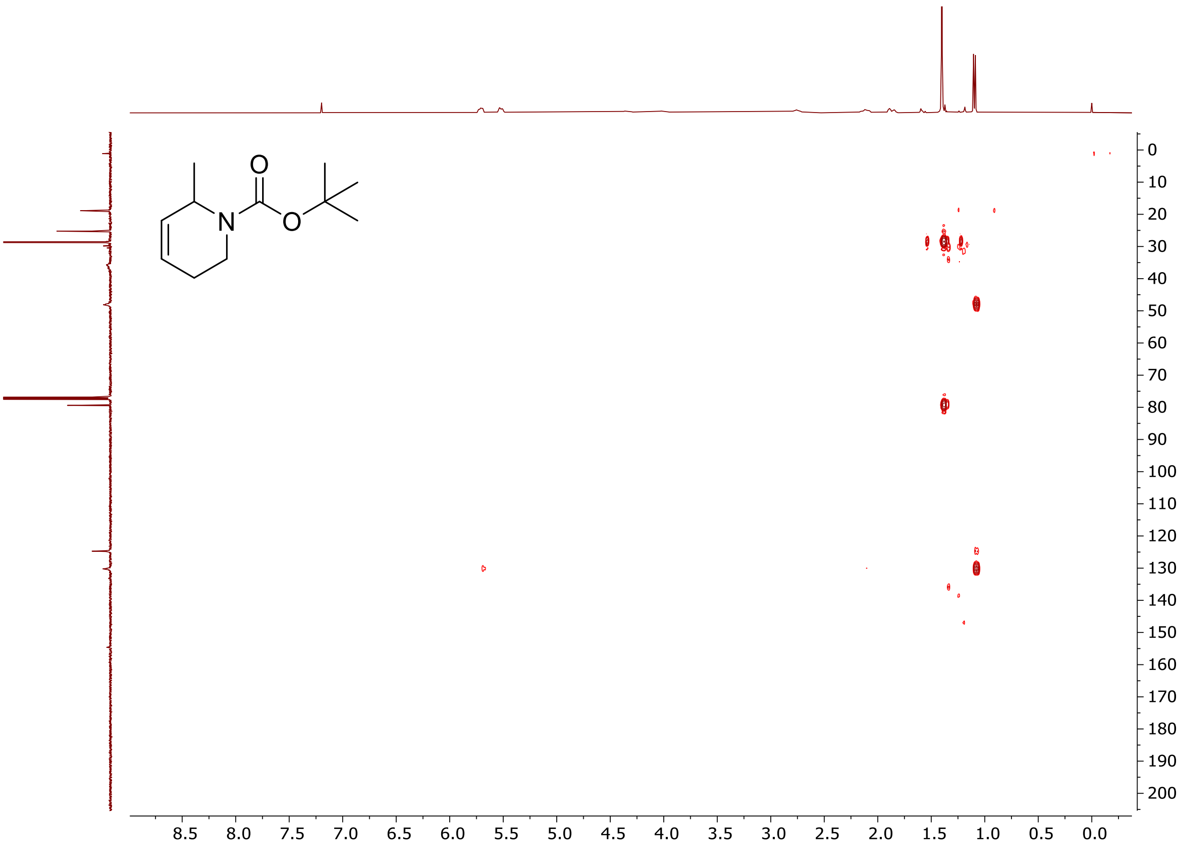

Figure S31: ^{1}H-^{13}C HMBC NMR spectrum (400 MHz) of **1d** in $CDCl_3$ after isolation via column chromatography.

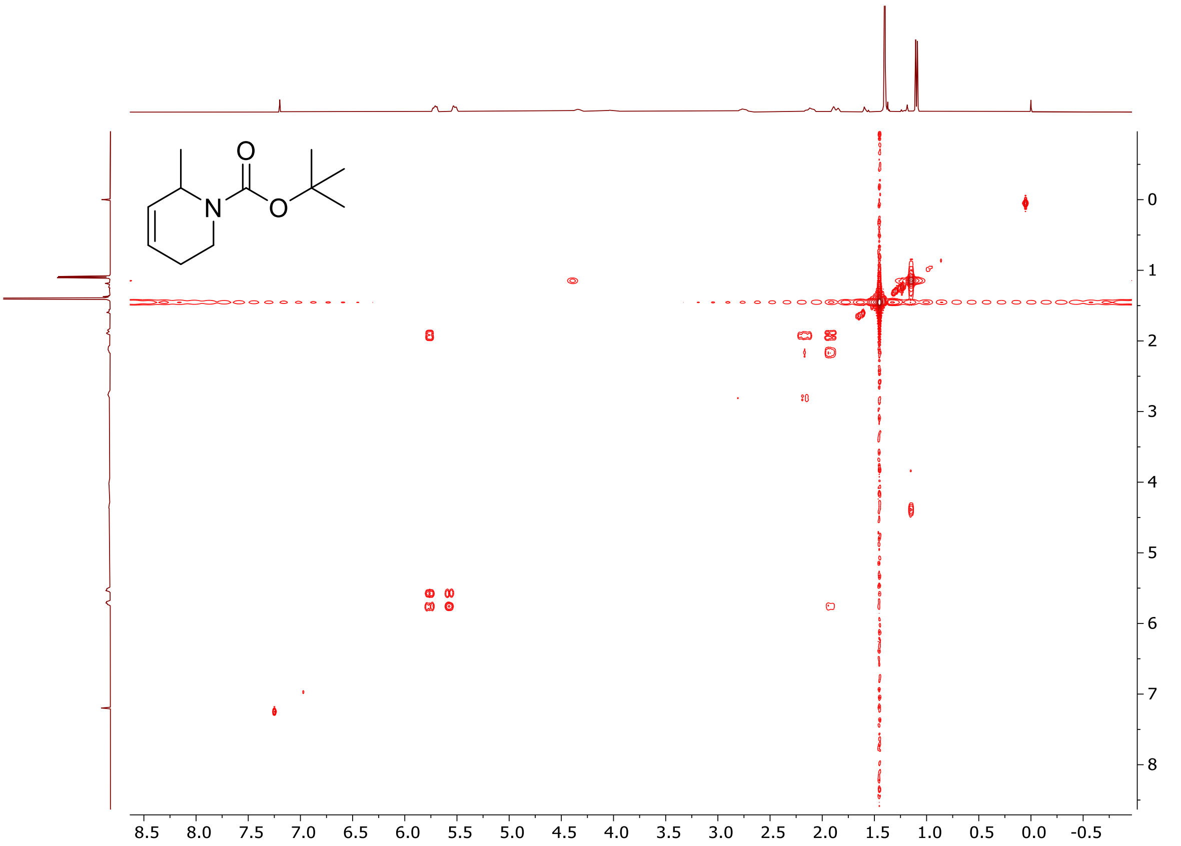

Figure S32: ^{1}H-^{1}H COSY NMR spectrum (400 MHz) of **1d** in $CDCl_3$ after isolation via column chromatography.

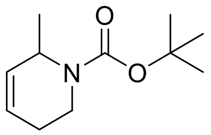

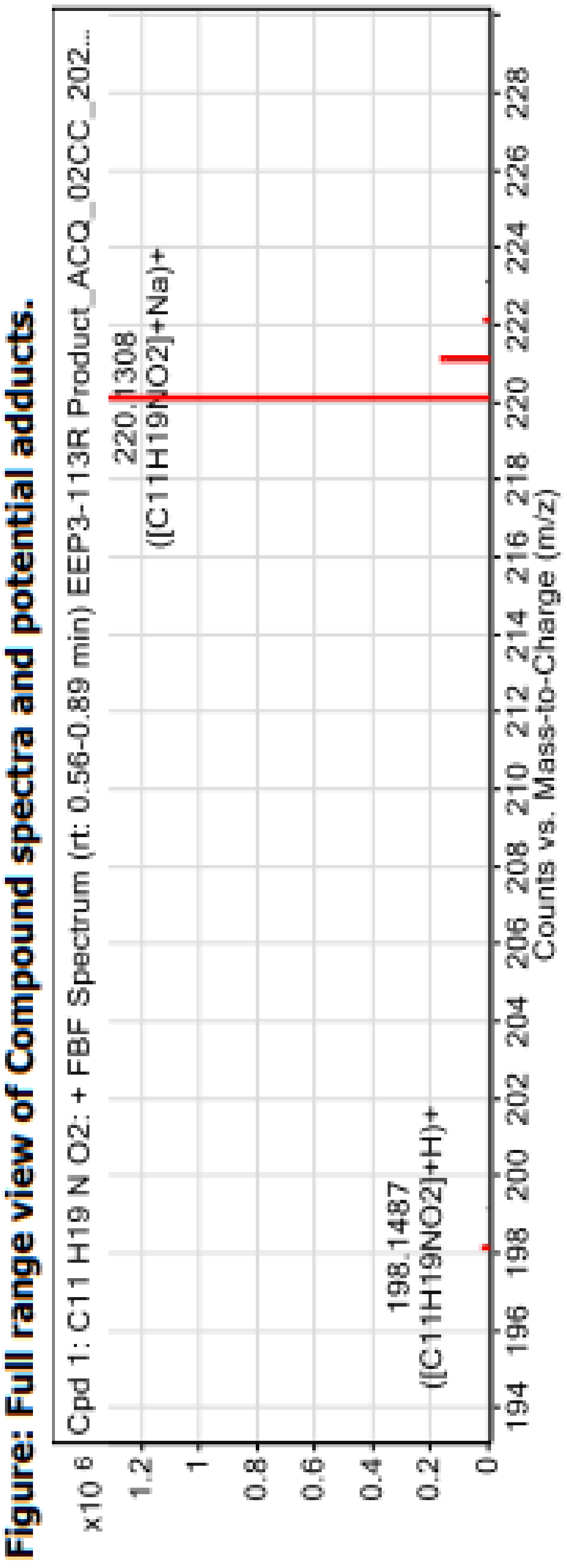

Figure S33: HRMS spectrum for compound ***1d****.*

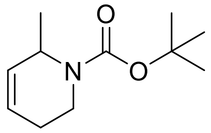

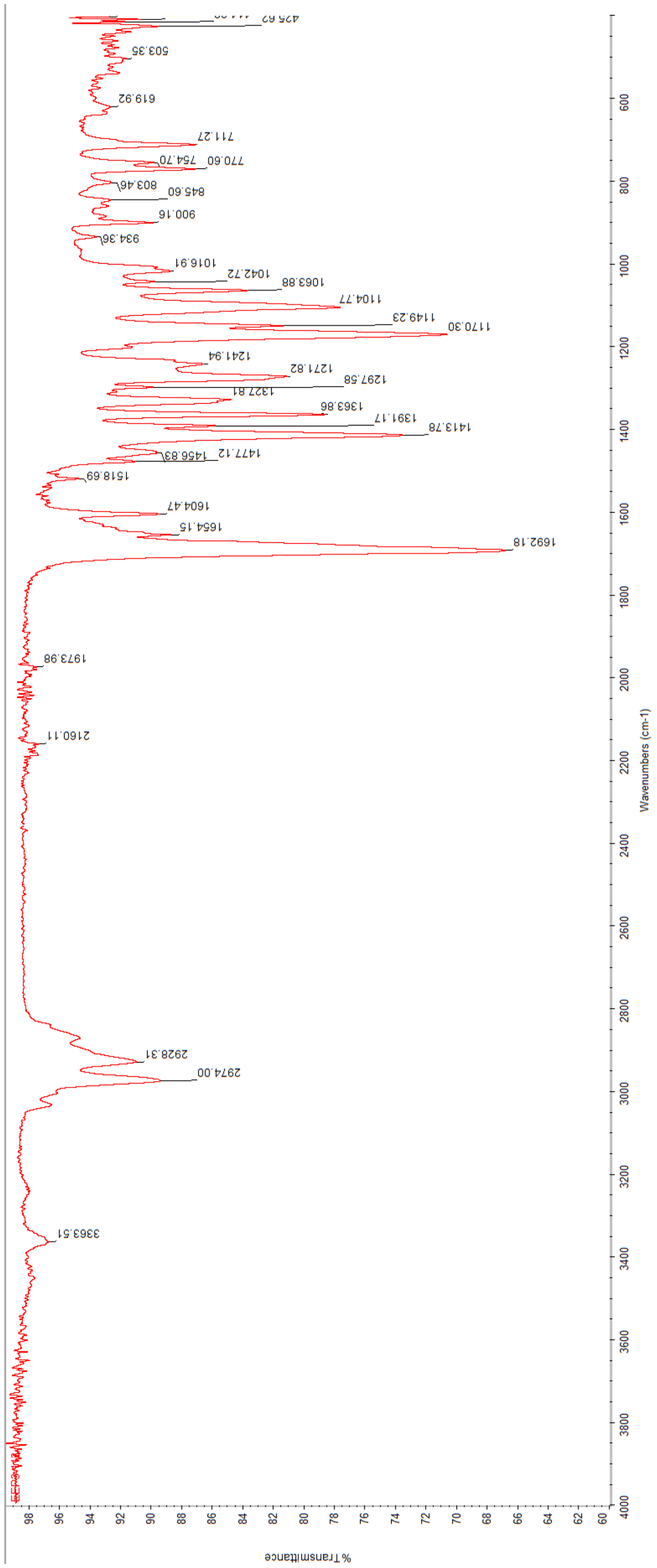

*Figure S34: IR spectrum of compound **1d**.*

tert-butyl-2-azabicyclo[2.2.2]oct-5-ene-2-carboxylate, **1e**

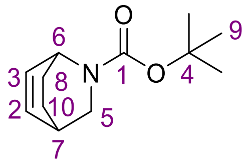

Following the general procedure and using cyclohexadiene (95.4 µL, 1 mmol per vial)

Yield: 1.071 g, 17% (colourless oil).

Analytic data is in accordance with those reported in literature.[3]

R_f = 0.57 (10% ethyl acetate / 90% hexane)

^{1}H NMR ($CDCl_3$, 400 MHz): δ 6.50 – 6.25 (m, 2H, $C^{2/3}$-H), 4.70 - 4.54 (m, 1H, C^6-H), 3.21 (d, *J* = 10.3 Hz, 1H), 3.01 – 2.86 (m, 1H), 2.69 (d, *J* = 15.6 Hz, 1H), 2.00 – 1.85 (m, 1H), 1.64 – 1.54 (m, 1H), 1.43 (m, 10H), 1.34 (d, *J* = 8.8 Hz, 1H).

$^{13}C\{^1H\}$ NMR ($CDCl_3$, 101 MHz) δ 155.0/154.7 (C^1), 134.2/133.7 (C^2), 133.2/132.5 (C^3), 79.2/79.1 (C^4), 48.4/ 48.0 (C^5), 46.2/44.7 (C^6), 30.9/30.6 (C^7), 28.7 (C^8), 27.7/26.9 (C^9), 22.1/22.1 (C^{10}).

HRMS (ESI+) m/z: $[M + Na]^+$ Calcd for $C_{12}H_{19}NO_2Na^+$ 232.1308; Found 232.1307.

FT-IR (cm^{-1}): 2963.5, 2869.6, 1686.2, 1396.7, 1364.6, 1133.9 cm^{-1}.

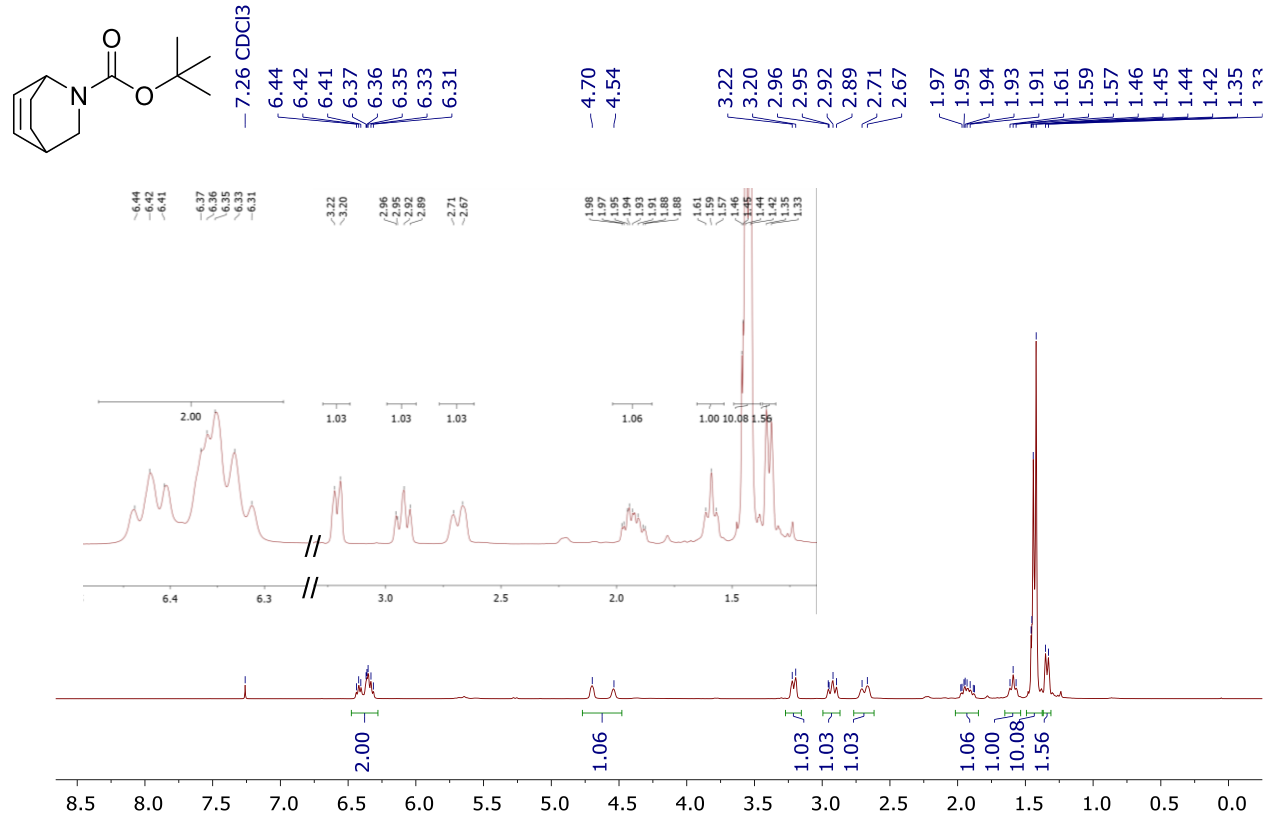

Figure S35: ^{1}H NMR spectrum (400 MHz) of **1e** in $CDCl_3$ after isolation via column chromatography. Zoom region shown in inset.

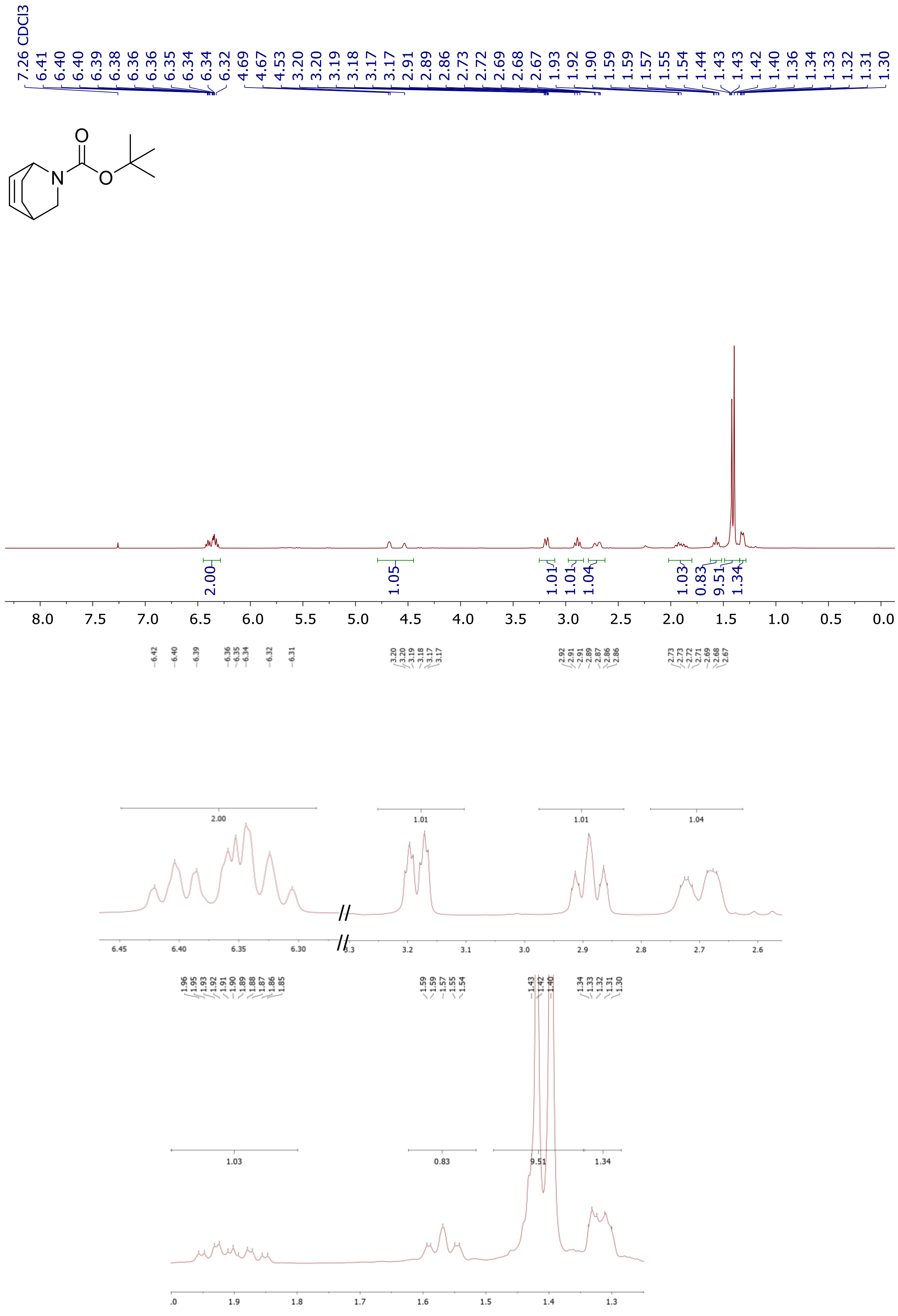

Figure S36: ^{1}H NMR spectrum (400 MHz) of **1e** in $CDCl_3$ after isolation via column chromatography. Spectrum recorded at -25°C. Full spectral range (top spectrum) with zoom regions shown below.

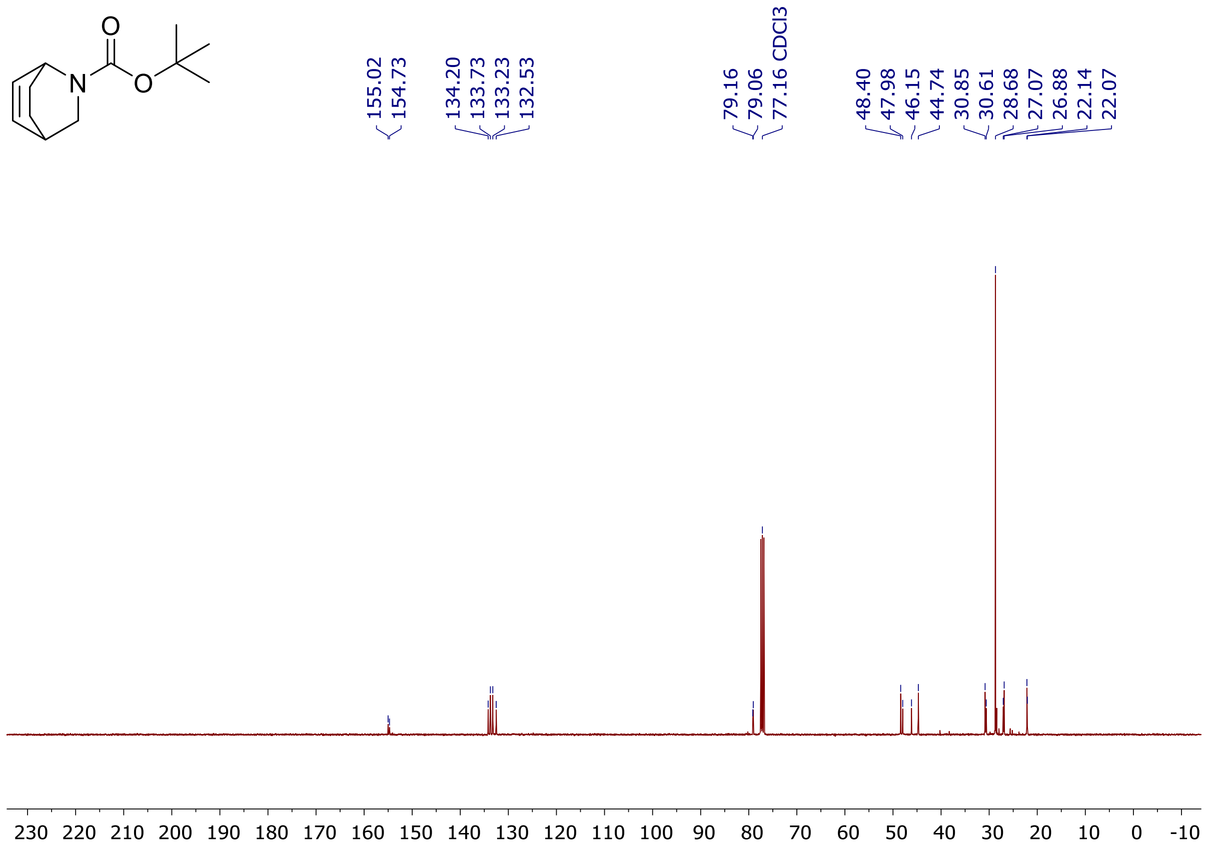

Figure S37: $^{13}C\{^{1}H\}$ NMR spectrum (400 MHz) of **1e** in $CDCl_3$ after isolation via column chromatography.

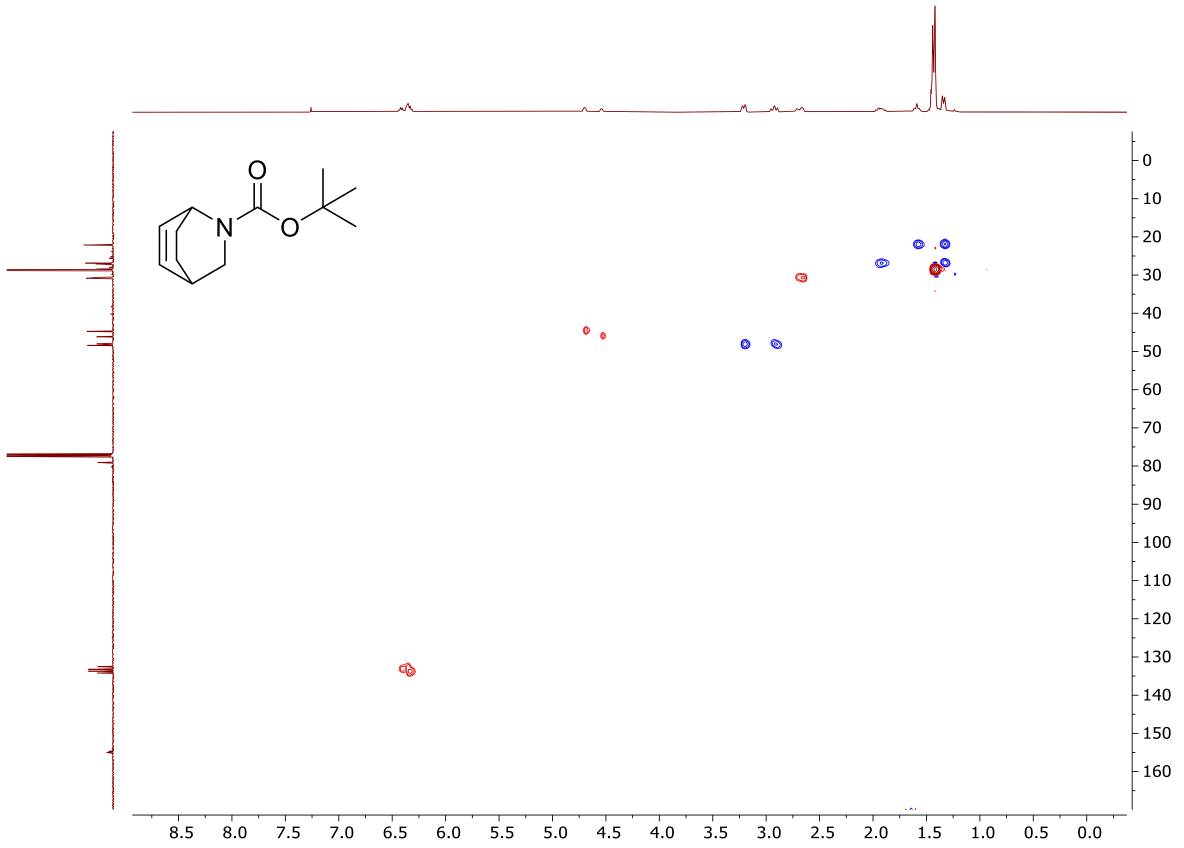

Figure S38: *^{1}H-^{13}C HSQC* NMR spectrum (400 MHz) of **1e** in $CDCl_3$ after isolation via column chromatography.

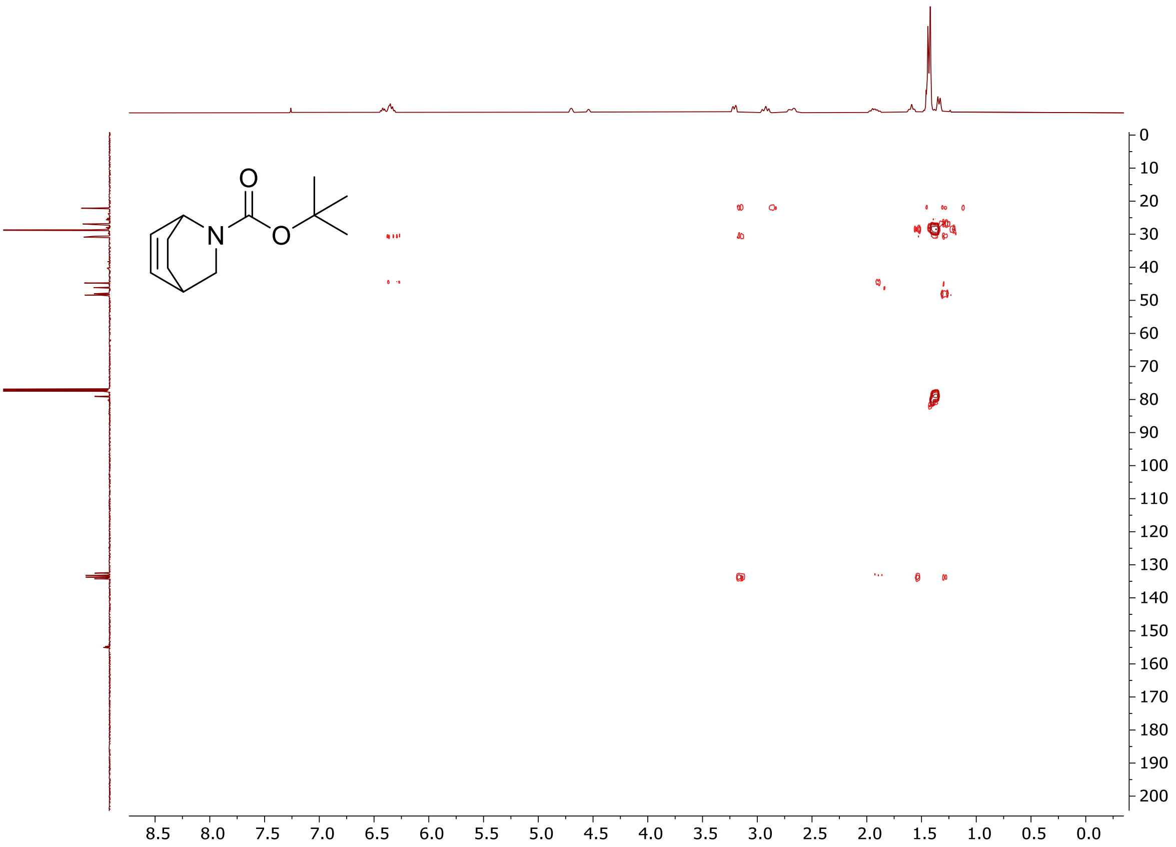

Figure S39: ^{1}H-^{13}C HMBC NMR spectrum (400 MHz) of **1e** in $CDCl_3$ after isolation via column chromatography.

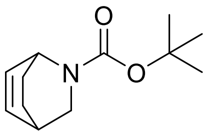

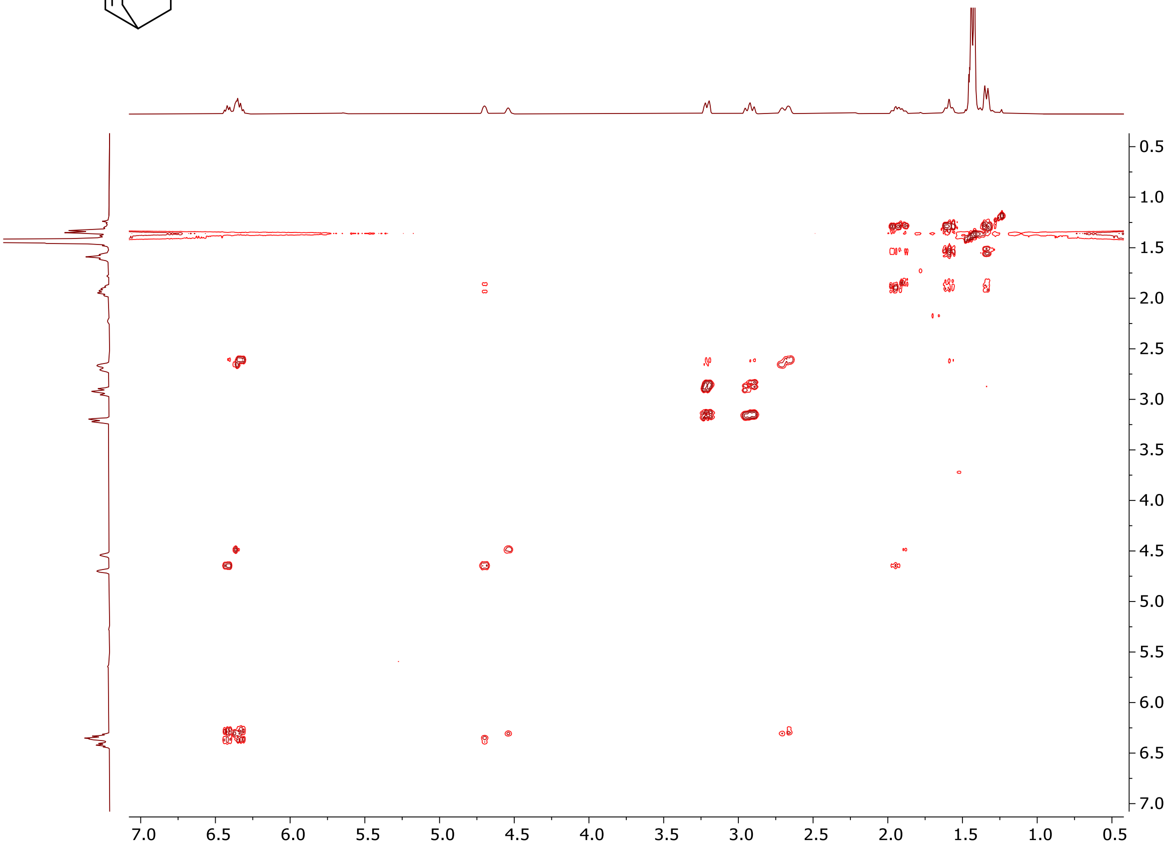

Figure S40: ^1H-^1H COSY NMR spectrum (400 MHz) of **1e** in $CDCl_3$ after isolation via column chromatography.

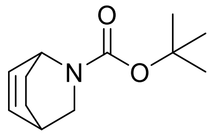

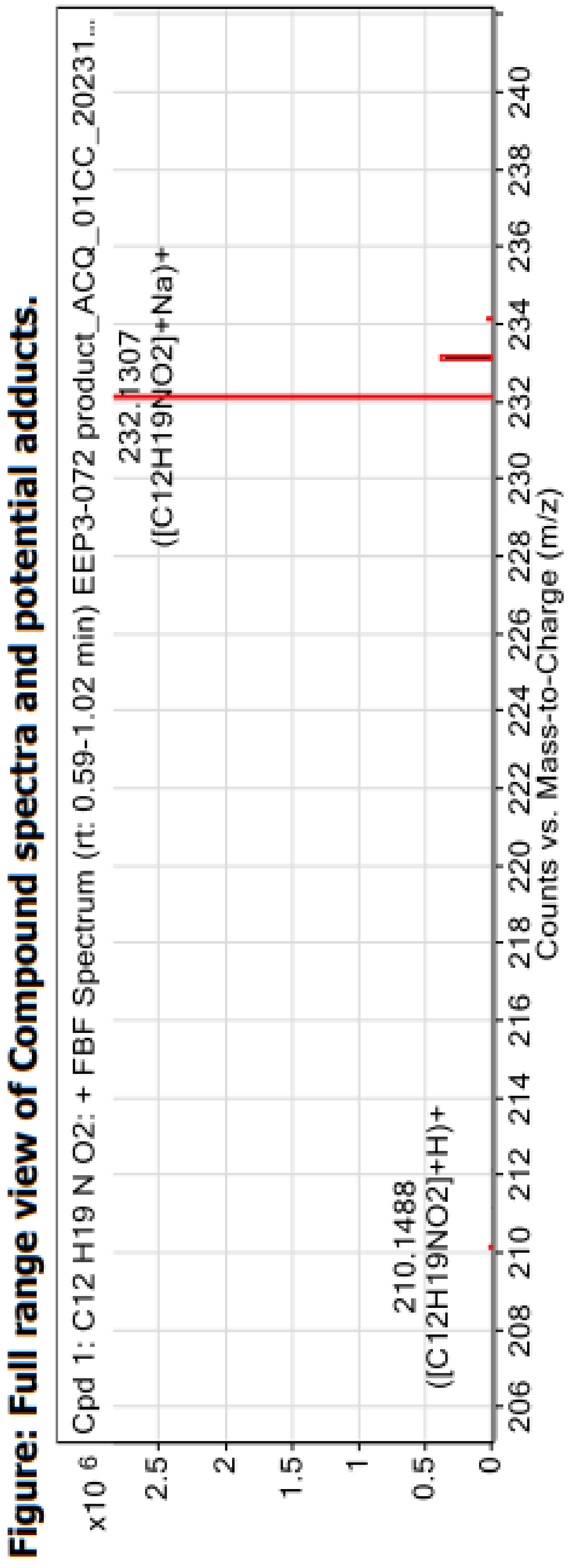

Figure S41: HRMS spectrum for compound ***1e****.*

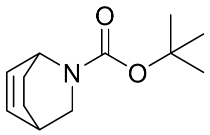

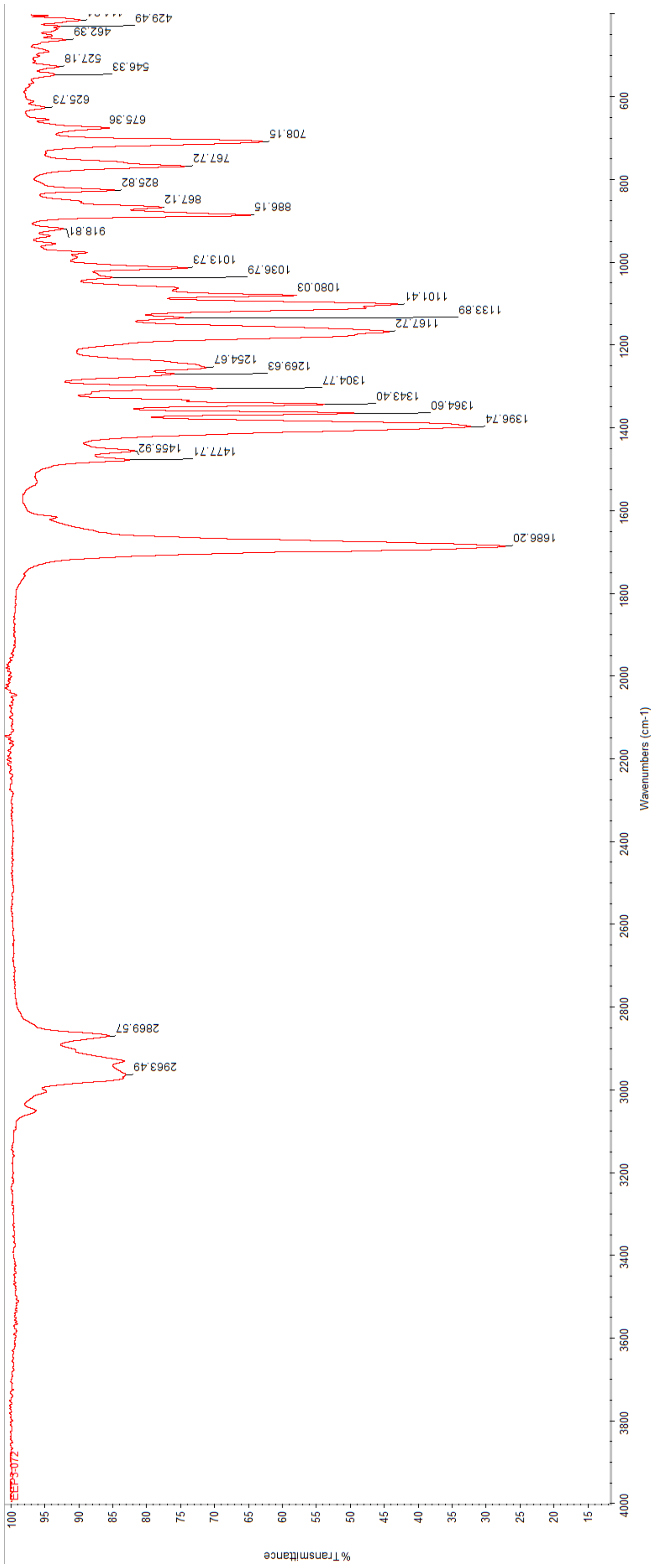

Figure S42: IR spectrum for Compound **1e**.

6. Computational Details

Quantum chemical calculations were carried out with the Gaussian 16[4] program. Molecular geometries are optimised employing density functional theory (DFT) with the generalised gradient approximation (GGA) in conjunction with the PBE[5,6] functional, an atom-pairwise dispersion correction (D3)[7] and a polarised continuum solvent model (PCM),[8] together with a split-valence double-zeta basis set (def2-SVP)[9] and the corresponding auxiliary Coulomb-fitting basis set of Weigend.[10] Zero-point vibrational energies and thermal contributions to Gibbs free energies at 298.15 K were obtained at this level of theory. Refined relative energies and properties including frontier molecular orbitals are obtained with the hybrid GGA functional PBE0[11,12] and a triple-zeta valence basis set (def2-TZVP),[9] utilising the same conditions (D3 dispersion correction and PCM solvent model).

To account for the volumetric effect that arises from the change of standard state in the gas phase at 1 atm to 1 mol L^{-1} in solution, we apply a standard-state conversion of 1.894 kcal mol L^{-1} per additional free reactant particle in order to account for the overestimation of entropic contributions to Gibbs free energies.[13]

Reaction Energies (Barriers and Thermochemistry) Aza-Diels-Alder Reaction

The computed barrier of 29.4 kcal mol^{-1} for the aza-Diels-Alder reaction between methanimine and 2,3-dimethyl butadiene is in agreement with a slow reaction at elevated temperatures. Note, however, that when assuming an acid-catalysed reaction in aqueous media, the methanimine reactant will mostly occur in its protonated form. In this scenario the reaction barrier is expected to be significantly lowered, see, e.g. McCarrick, Wu and Houk,[14] but the overall reaction is reversible at elevated temperatures.[15,16] Thus, both, the neutral and the proton-assisted reaction are in agreement with the experimentally observed yields.

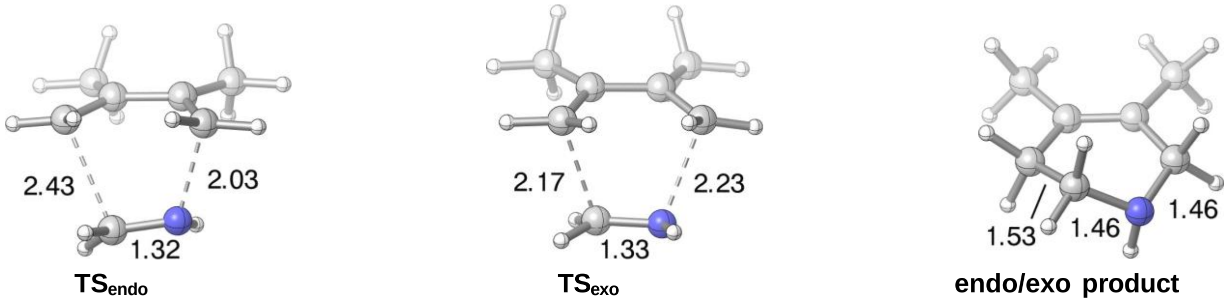

Figure S43: Transition structures and product for aza-Diels-Alder reaction of methanimine with trans-2,3-dimethyl butadiene.

Table S1. Relative reaction enthalpies, $\Delta_r H^{298}$, and Gibbs free energies, $\Delta_r G^{298}$, in kcal mol^{-1}, computed at the PBE0-D3/def2-TVP/PCM(acetonitrile) // PBE-D3/def2-SVP/PCM(acetonitrile) level. Gibbs free energies for transition structures and reaction product are corrected for standard-state conditions by −1.84 kcal mol^{-1}.

***trans*-2,3-dimethyl butadiene + methanimine**		
Species	$\Delta_r H^{298}$[a]	$\Delta_r G^{298}$[b]
TS_{endo}	18.9	29.4
TS_{exo}	20.7	31.2
Diels-Alder product	−36.2	−24.8

[a] $\Delta H^{298} = \Delta E(\text{tot}) + \text{ZPVE} + \Delta E(\text{vib}) + \Delta E(\text{rot}) + \Delta E(\text{trans}) + \text{R}\,T$

[b] $\Delta G^{298} = \Delta H^{298} - T\,\Delta S - 1.89$; see Computational Details.

Cyclotrimerisation Reaction

Cyclotrimerisation of methanimine (**2a**) is associated with a sizeable barrier of 39.3 kcal mol^{-1}, but the cyclic species **3a** is exergonic by Δ_rG = −30.3 kcal mol^{-1} relative to **2a**. The Bpin-protected methanimine adduct **2b** has a modest barrier to cyclotrimerisation of $\Delta^{‡}G$ = −26.4 kcal mol^{-1} and the formation of **3b** is highly exergonic (Δ_rG = −57.1 kcal mol^{-1}). **2b** is therefore more reactive than free methanimine **2a**. While the transition state for the cyclotrimerisation of **2c** is higher at 37.1 kcal mol^{-1}, **3c** sits at −27.9 kcal mol^{-1} relative to **2c**. Clearly these simple protecting group strategies will not significantly modify the HOMO-LUMO gap (see Figure 3) and thus [2+2+2] cyclisation is viable. Assuming the presence of a certain proportion of protonated methaniminie, the formation of a protonated cyclic variant of **3a** can be formed in a sequential reaction with considerably lower barriers, see Figure 4, thus accelerating the cyclisation reaction.

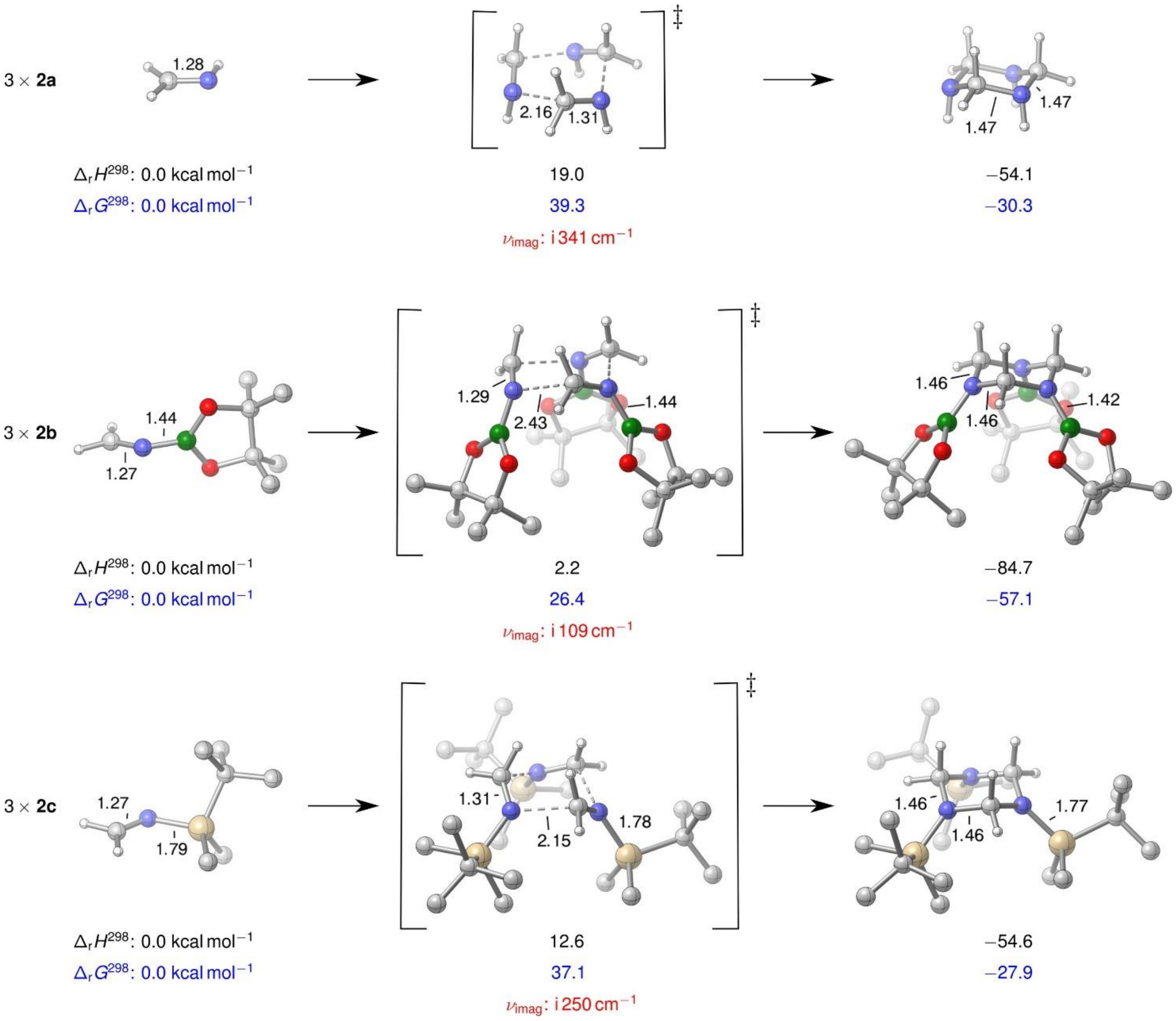

*Figure S44: Reaction barriers and thermochemistry for trimerisation of methanimine **2a** and protected adducts **2b** and **2c**, computed at the PBE0-D3/def2-TZVP/PCM(acetonitrile) // PBE-D3/def2-SVP/PCM(acetonitrile) level. Gibbs free energies for transition structures and cyclisation products are corrected for standard-state conditions by −3.79 kcal mol^{-1}.*

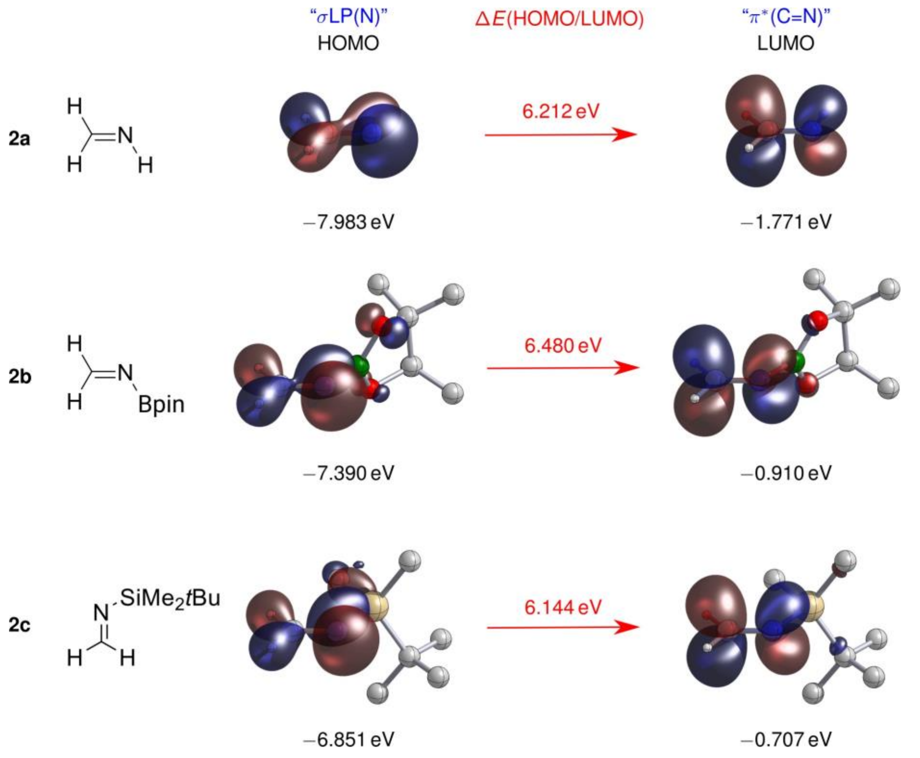

Figure S45: Frontier molecular orbitals and energies for the highest occupied and the lowest unoccupied MOs of methanimine ***2a*** *and protected adducts* ***2b*** *and* ***2c****.*

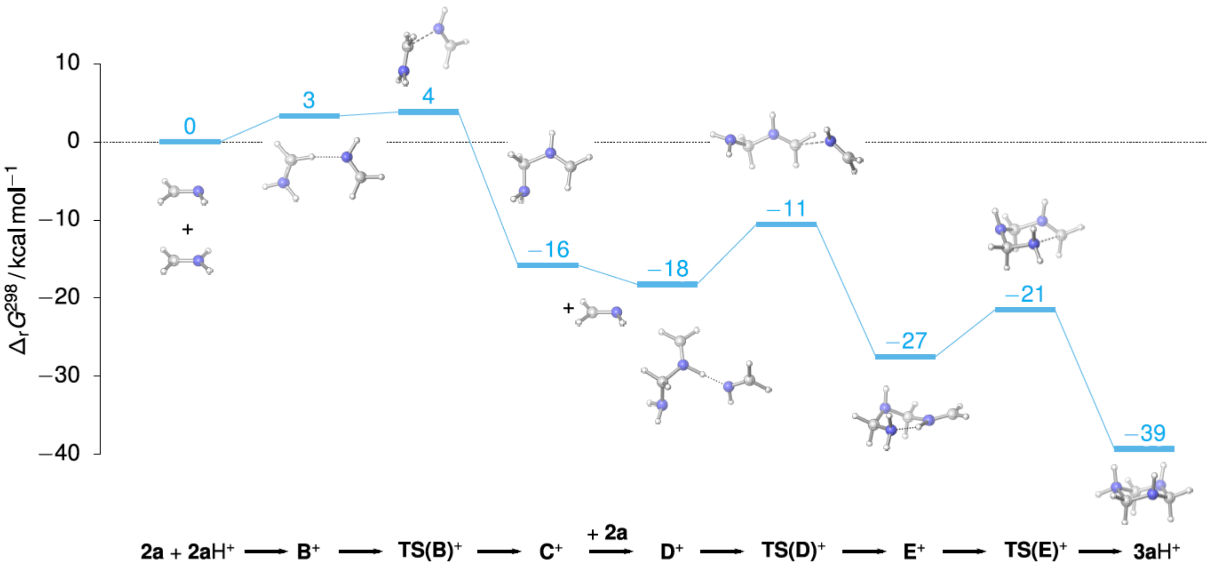

*Figure S46: PES for stepwise, proton-assisted trimerisation of methanimine, i.e., reaction of 2 × methanimine (**2a**) + formiminium cation (**2a**H^+) to yield the protonated cylclisation product **3a**H^+, computed at the PBE0-D3/def2-TZVP/PCM(acetonitrile)//PBE-D3/def2-SVP/PCM(acetonitrile) level. Gibbs free energies include corrections for standard-state conditions by −1.84 kcal mol^{-1} for **B**$^+$ – **C**$^+$ and by −3.79 kcal mol^{-1} for **D**$^+$ – **3a**H^+.*

Table S2: DFT results for aza-Diels-Alder reaction of methanimine with *trans*-2,3-dimethyl 1,3-butadiene. Total energies, thermal and Gibbs energy corrections (Hartree) computed with PBE0-D3(PCM,solvent=water)/def2-TZVP // PBE-D3(PCM,solvent=water)/def2-SVP are given, along with corresponding relative energies (kcal mol^{-1}) with respect to the reactants. Relative Gibbs free energies corrected for standard-state conditions by −1.84 kcal mol^{-1}, G^{298}_{std}, are also given.

**PBE0-D3(water)/def2-TZVP // PBE-D3(water)/def2-SVP**							
Species	E_{tot}	H^{298}_{corr}	G^{298}_{corr}	E_{rel}	H^{298}_{rel}	G^{298}_{rel}	G^{298}_{std}
**methaniminie (2a)**	−94.554 612	0.042 541	0.016 713	0.0	0.0	0.0	0.0
***trans*-dimethylbutadiene**	−234.417 149	0.145 315	0.107 356	0.0	0.0	0.0	0.0
***cis*-dimethylbudatiene**	−234.413 072	0.144 984	0.106 538	2.6	2.4	2.1	2.1
**TS_{endo}**	−328.942 954	0.189 097	0.145 039	18.1	18.9	31.2	29.4
**TS_{exo}**	−328.940 040	0.189 056	0.145 131	19.9	20.7	33.1	31.2
**aza-D/A product**	−329.035 591	0.193 943	0.151 338	−40.1	−36.2	−22.9	−24.8

Table S3: DFT results for cyclotrimerisation reaction of methanimine **2a**, Bpin-adduct **2b** and TBDMS-adduct **2c**. Total energies, thermal and Gibbs energy corrections (Hartree) computed with PBE0-D3(PCM,solvent=acetonitrile)/def2-TZVP // PBE-D3(PCM,solvent=acetonitrile)/def2-SVP are given, along with corresponding relative energies (kcal mol^{-1}) with respect to the reactants. Relative Gibbs free energies corrected for standard-state conditions by −3.79 kcal mol^{-1}, G^{298}_{std}, are also given.

**PBE0-D3(acetonitrile)/def2-TZVP // PBE-D3(acetonitrile)/def2-SVP**							
Species	E_{tot}	H^{298}_{corr}	G^{298}_{corr}	E_{rel}	H^{298}_{rel}	G^{298}_{rel}	G^{298}_{std}
3 × **2a** ⟶ **3a** (R = H)							
**methaniminie (2a)**	−94.554 477	0.042 541	0.016 713	0.0	0.0	0.0	0.0
**TS(2a,3a)**	−283.637 233	0.131 757	0.092 625	16.4	19.0	43.1	39.3
**3a**	−283.761 432	0.139 430	0.105 929	−61.5	−54.1	−26.5	−30.3
3 × **2b** ⟶ **3b** (R = Bpin)							
**2b**	−504.947 873	0.218 822	0.168 480	0.0	0.0	0.0	0.0
**TS(2b,3b)**	−1514.844 284	0.660 665	0.554 165	−0.4	2.2	30.2	26.4
**3b**	−1514.990 723	0.668 582	0.567 538	−92.3	−84.7	−53.3	−57.1
3 × **2c** ⟶ **3c** (R = TBDMS)							
**2c**	−620.866 546	0.234 018	0.179 742	0.0	0.0	0.0	0.0
**TS(2c,3c)**	−1862.584 979	0.707 398	0.589 747	9.2	12.6	40.9	37.1
**3c**	−1862.698 558	0.713 950	0.599 717	−62.1	−54.6	−24.1	−27.9

Table S4: DFT results for proton-assisted stepwise cyclotrimerisation reaction of 2 × methanimine **2a**, with formiminium cation **2a**H$^+$. Total energies, thermal and Gibbs energy corrections (Hartree) computed with PBE0-D3(PCM,solvent=acetonitrile)/def2-TZVP // PBE-D3(PCM,solvent=acetonitrile)/def2-SVP are given, along with corresponding relative energies (kcal mol^{-1}) with respect to the reactants. Relative Gibbs free energies corrected for standard-state conditions by −1.84 kcal mol^{-1} for **B**$^+$ – **C**$^+$ and by −3.79 kcal mol^{-1} for **D**$^+$ – **3a**H$^+$, G^{298}_{std}, are also given.

**PBE0-D3(acetonitrile)/def2-TZVP // PBE-D3(acetonitrile)/def2-SVP**							
Species	E_{tot}	H^{298}_{corr}	G^{298}_{corr}	E_{rel}	H^{298}_{rel}	G^{298}_{rel}	G^{298}_{std}
2 × **2a** + **2a**H$^+$ ⟶ **3a**H$^+$							
**2a**	−94.554 476	0.042 532	0.016 703	0.0	0.0	0.0	0.0
**2aH$^+$**	−94.997 283	0.056 650	0.031 100	0.0	0.0	0.0	0.0
**B$^+$**	−189.559 417	0.100 257	0.063 787	−4.8	−4.1	5.2	3.3
**TS(B)$^+$**	−189.557 644	0.100 194	0.062 798	−3.7	−3.1	5.7	3.8
**C$^+$**	−189.596 143	0.103 187	0.070 030	−27.9	−25.3	−13.9	−15.8
**D$^+$**	−284.165 690	0.146 297	0.100 945	−37.3	−34.4	−14.4	−18.2
**TS(D)$^+$**	−284.152 326	0.146 638	0.099 840	−28.9	−25.8	−6.8	−10.5
**E$^+$**	−284.190 138	0.148 853	0.110 633	−52.7	−48.2	−23.7	−27.5
**TS(E)$^+$**	−284.180 968	0.149 463	0.111 025	−46.9	−42.0	−17.7	−21.5
**2aH$^+$**	−284.216 736	0.153 553	0.118 411	−69.3	−61.9	−35.5	−39.3

4. References

[1] Kuhn, K. M.; Champagne, T. M.; Hong, S. H.; Wei, W. H.; Nickel, A.; Lee, C. W.; Virgil, S. C.; Grubbs, R. H.; Pederson, R. L. Low catalyst loadings in olefin metathesis: synthesis of nitrogen heterocycles by ring-closing metathesis. *Org. Lett.* **2010**, *12*, 984-987.

[2] Luo, J. J.; Jing, D.; Lu, C.; Zheng, K. Photoinduced Metal-Free Decarboxylative Transformations: Rapid Access to Amines, Alkyl Halides, and Olefins. *Eur. J. Org. Chem.* **2023**, *26*, e202300167.

[3] Arakawa, Y.; Yasuda, M.; Ohnishi, M.; Yoshifuji, S. Stereospecific synthesis of cis-2,4-pyrrolidinedicarboxylic acid and cis-2,5-piperidinedicarboxylic acid. *Chem. Pharm. Bull.* **1997**, *45*, 255-259.

[4] Gaussian 16, Revision C.01. Frisch, M. J.; Trucks, G. W.; Schlegel, H. B.; Scuseria, G. E.; Robb, M. A.; Cheeseman, J. R.; Scalmani, G.; Barone, V.; Petersson, G. A.; Nakatsuji, H.; Li, X.; Caricato, M.; Marenich, A. V.; Bloino, J.; Janesko, B. G.; Gomperts, R.; Mennucci, B.; Hratchian, H. P.; Ortiz, J. V.; Izmaylov, A. F.; Sonnenberg, J. L.; Williams-Young, D.; Ding, F.; Lipparini, F.; Egidi, F.; Goings, J.; Peng, B.; Petrone, A.; Henderson, T.; Ranasinghe, D.; Zakrzewski, V. G.; Gao, J.; Rega, N.; Zheng, G.; Liang, W.; Hada, M.; Ehara, M.; Toyota, K.; Fukuda, R.; Hasegawa, J.; Ishida, M.; Nakajima, T.; Honda, Y.; Kitao, O.; Nakai, H.; Vreven, T.; Throssell, K.; Montgomery, Jr., J. A.; Peralta, J. E.; Ogliaro, F.; Bearpark, M. J.; Heyd, J. J.; Brothers, E. N.; Kudin, K. N.; Staroverov, V. N.; Keith, T. A.; Kobayashi, R.; Normand, J.; Raghavachari, K.; Rendell, A. P.; Burant, J. C.; Iyengar, S. S.; Tomasi, J.; Cossi, M.; Millam, J. M.; Klene, M.; Adamo, C.; Cammi, R.; Ochterski, J. W.; Martin, R. L.; Morokuma, K.; Farkas, Ö.; Foresman, J. B.; Fox, D. J. (Gaussian, Inc., Wallingford, CT), **2019**, see http://www.gaussian.com.

[5] Perdew, J. P.; Burke, K.; Ernzerhof, M., Generalized Gradient Approximation Made Simple. *Phys. Rev. Lett.* **1996**, *77* (18), 3865–3868.

[6] Perdew, J. P.; Burke, K.; Ernzerhof, M., Erratum: Generalized Gradient Approximation Made Simple [Phys. Rev. Lett. 77, 3865 (1996)]. *Phys. Rev. Lett.* **1997**, *78* (7), 1396–1396.

[7] Grimme, S.; Antony, J.; Ehrlich, S.; Krieg, H., A consistent and accurate *ab initio* parametrization of density functional dispersion correction (DFT-D) for the 94 elements H–Pu. *J. Chem. Phys.* **2010**, *132* (15), 154 104.

[8] Tomasi, J.; Mennucci, B.; Cammi, R., Quantum Mechanical Continuum Solvation Models. *Chem. Rev.* **2005**, *105* (8), 2999–3094.

[9] Weigend, F.; Ahlrichs, R., Balanced basis sets of split valence, triple zeta valence and quadruple zeta valence quality for H to Rn: Design and assessment of accuracy. *Phys. Chem. Chem. Phys.* **2005**, *7* (18), 3297–3305.

[10] Weigend, F., Accurate Coulomb-fitting basis sets for H to Rn. *Phys. Chem. Chem. Phys.* **2006**, *8* (9), 1057–1065.

[11] Perdew, J. P.; Ernzerhof, M.; Burke, K., Rationale for mixing exact exchange with density functional approximations. *J. Chem. Phys.* **1996**, *105* (22), 9982–9985.

[12] Adamo, C.; Barone, V., Toward reliable density functional methods without adjustable parameters: The PBE0 model. *J. Chem. Phys.* **1999**, *110* (13), 6158–6170.

[13] Harvey, J. N.; Himo, F.; Maseras, F.; Perrin, L., Scope and Challenge of Computational Methods for Studying Mechanism and Reactivity in Homogeneous Catalysis. *ACS Catal.* **2019**, *9* (8), 6803–6813.

[14] McCarrick, M. A.; Wu, Y. D.; Houk, K. N., Hetero-Diels-Alder reaction transition structures: reactivity, stereoselectivity, catalysis, solvent effects, and the exo-lone-pair effect. *The Journal of Organic Chemistry* **1993**, *58* (12), 3330–3343.

[15] Grieco, P. A.; Parker, D. T.; Cornwell, M.; Ruckle, R., Retro aza Diels-Alder reactions: acid catalyzed heterocycloreversion of 2-azanorbornenes in water at ambient temperature. *J. Am. Chem. Soc.* **1987**, *109* (19), 5859–5861.

[16] Grieco, P. A.; Clark, J. D., Retro aza Diels-Alder reactions of 2-azanorbornenes: improved methods for the unmasking of primary amines. *The Journal of Organic Chemistry* **1990**, *55* (8), 2271–2272.
